# DUNE: a versatile neuroimaging encoder captures brain complexity across 3 major diseases: cancer, dementia, and schizophrenia

**DOI:** 10.1093/gigascience/giaf116

**Published:** 2025-10-15

**Authors:** Thomas Barba, Bryce A Bagley, Sandra Steyaert, Francisco Carrillo-Perez, Christoph Sadée, Michael Iv, Olivier Gevaert

**Affiliations:** Stanford Center for Biomedical Informatics Research (BMIR), Department of Medicine, Stanford University, Stanford, CA 94305, USA; Department of Internal Medicine, Edouard Herriot Hospital, Lyon 69003, France; Stanford Center for Biomedical Informatics Research (BMIR), Department of Medicine, Stanford University, Stanford, CA 94305, USA; Stanford Center for Biomedical Informatics Research (BMIR), Department of Medicine, Stanford University, Stanford, CA 94305, USA; Stanford Center for Biomedical Informatics Research (BMIR), Department of Medicine, Stanford University, Stanford, CA 94305, USA; Stanford Center for Biomedical Informatics Research (BMIR), Department of Medicine, Stanford University, Stanford, CA 94305, USA; Department of Radiology, Stanford University, Stanford, CA 94305, USA; Stanford Center for Biomedical Informatics Research (BMIR), Department of Medicine, Stanford University, Stanford, CA 94305, USA; Department of Biomedical Data Science, Stanford University, Stanford, CA 94305, USA

**Keywords:** neuroimaging, feature extraction, deep learning, autoencoders

## Abstract

**Background:**

Magnetic resonance imaging (MRI) of the brain contains complex data that pose significant challenges for computational analysis. While models proposed for brain MRI analyses yield encouraging results, the high complexity of neuroimaging data hinders generalizability and clinical application. We introduce *DUNE*, a neuroimaging-oriented workflow that transforms raw brain MRI scans into standardized compact patient-level embeddings through integrated preprocessing and deep feature extraction, thereby enabling their processing by basic machine learning algorithms. A UNet-based autoencoder was trained using 3,814 selected scans of morphologically normal (healthy volunteers) or abnormal (glioma patients) brains, to generate comprehensive compact representations of the full-sized images. To evaluate their quality, these embeddings were utilized to train machine learning models to predict a wide range of clinical variables.

**Results:**

Embeddings were extracted for cohorts used for the model development (21,102 individuals), along with 3 additional independent cohorts (Alzheimer’s disease, schizophrenia, and glioma cohorts, 1,322 individuals), to evaluate the model’s generalization capabilities. The embeddings extracted from healthy volunteers’ scans could predict a broad spectrum of clinical parameters, including volumetry metrics, cardiovascular disease (area under the receiver operating characteristic curve [AUROC] = 0.80) and alcohol consumption (AUROC = 0.99), and more nuanced parameters such as the Alzheimer’s predisposing APOE4 allele (AUROC = 0.67). Embeddings derived from the validation cohorts successfully predicted the diagnoses of Alzheimer’s dementia (AUROC = 0.92) and schizophrenia (AUROC = 0.64). Embeddings extracted from glioma scans successfully predicted survival (C-index = 0.608) and IDH molecular status (AUROC = 0.92), matching the performances of previous task-oriented models.

**Conclusion:**

*DUNE* efficiently represents clinically relevant patterns from full-size brain MRI scans across several disease areas, opening ways for innovative clinical applications in neurology.

## Introduction

The morphology of the human brain is determined by genetic and embryological factors specific to each individual and is constantly remodeled throughout life in response to internal and environmental factors [1]. A broad range of pathological processes shape brain morphology, affecting the brain primarily (e.g., cancer and inflammatory and neurodegenerative diseases) or secondarily in systemic disorders (e.g., cardiovascular and systemic autoimmune diseases) [2–4]. Therefore, the morphology of the brain recapitulates numerous formative events that occur during an individual’s lifetime, providing a snapshot of their health at a particular time. Advances in brain imaging over the past decades have enabled doctors to better characterize and treat a wide range of conditions. In this setting, magnetic resonance imaging (MRI) has established it as a fundamental tool in the diagnosis and surveillance of cancers, neurodegenerative disorders, and inflammatory diseases of the central nervous system [5, 6]. With the ongoing development of new imaging sequences, the quality of brain imaging continues to improve, achieving unprecedented precision in depicting brain morphology. However, while these increasingly complex representations of the brain are of undeniable interest in the management of brain diseases, their full potential has yet to be realized.

MRI datasets are characterized by their computational complexity, stemming from the large number of voxels per image (≥5 million voxels per sequence) and the multisequence nature of clinical protocols (T1, fluid-attenuated inversion recovery [FLAIR], contrast injection sequences, etc.). This high-dimensional space, combined with the subtle morphological patterns that encode clinical information, necessitates sophisticated computational approaches for effective analysis.

In the era of artificial intelligence, authors have proposed deep learning models capable of leveraging these complex imaging data to predict clinical parameters, such as the detection of brain tumors [7, 8] and the diagnosis of neuropsychiatric and neurodegenerative diseases [9–11]. However, these models have been limited in their clinical translation due to several reasons. First, their training requires large datasets and extensive computational resources because of the considerable number of internal parameters that need to be trained. Second, their monothematic nature implicates that they learn to perform only one task at a time (e.g., diagnosis prediction or tumor segmentation). Third, these models are usually trained on a few specific datasets, which affects their performance on externally acquired images, a problem known as overfitting and lack of generalization [12].

General-purpose feature extractors could offer promising solutions to tackle the complexities of processing high-dimensional data. Such models can generate versatile representations that capture essential information from the input data, while reducing computational demands for downstream applications [13, 14].

In this study, we introduce *DUNE* (Deep feature extraction by UNet-based Neuroimaging-oriented autoEncoder), a comprehensive neuroimaging workflow that transforms multisequence raw brain MRI scans into standardized compact patient-level embeddings. The workflow integrates standardized preprocessing, unsupervised feature extraction using a UNet-based autoencoder, and multisequence feature aggregation to generate comprehensive patient-level embeddings [15–18]. To ensure its robustness, the model was trained on MRI scans of morphologically normal and abnormal brains issued from 3 datasets. The quality of the extracted embeddings was evaluated by employing them to infer numerous clinical parameters using simple machine learning models. Different autoencoder architectures were compared in terms of reconstruction capabilities and quality of embeddings. Based on comprehensive evaluations across multiple clinical applications, we identified a UNet architecture without skip connections (U-AE) as the optimal model for *DUNE*. Surprisingly, this architecture, while being the least effective in reconstructing MRI data, produced the most clinically relevant embeddings, underscoring the importance of using appropriate evaluation metrics commensurate with their intended use. To demonstrate its generalizability, *DUNE* was used to extract embeddings of scans of individuals from 3 independent cohorts representing 3 disease areas. These embeddings were used to successfully predict a broad spectrum of clinical parameters, including cognitive dysfunction, psychiatric disorders, genetic traits (APOE4 status), glioma molecular features (IDH status), and patient survival, with results matching or outperforming previously published models directly processing the raw imaging data. Finally, we demonstrate that augmenting the MRI data with synthetic images can enhance the quality of the produced embeddings, resulting in improved performances in downstream clinical inference tasks. In summary, *DUNE* provides efficient general-purpose embeddings of multimodal brain MRI data independent of the downstream use case.

## Data and Methods

### Study overview

**Figure 1:**
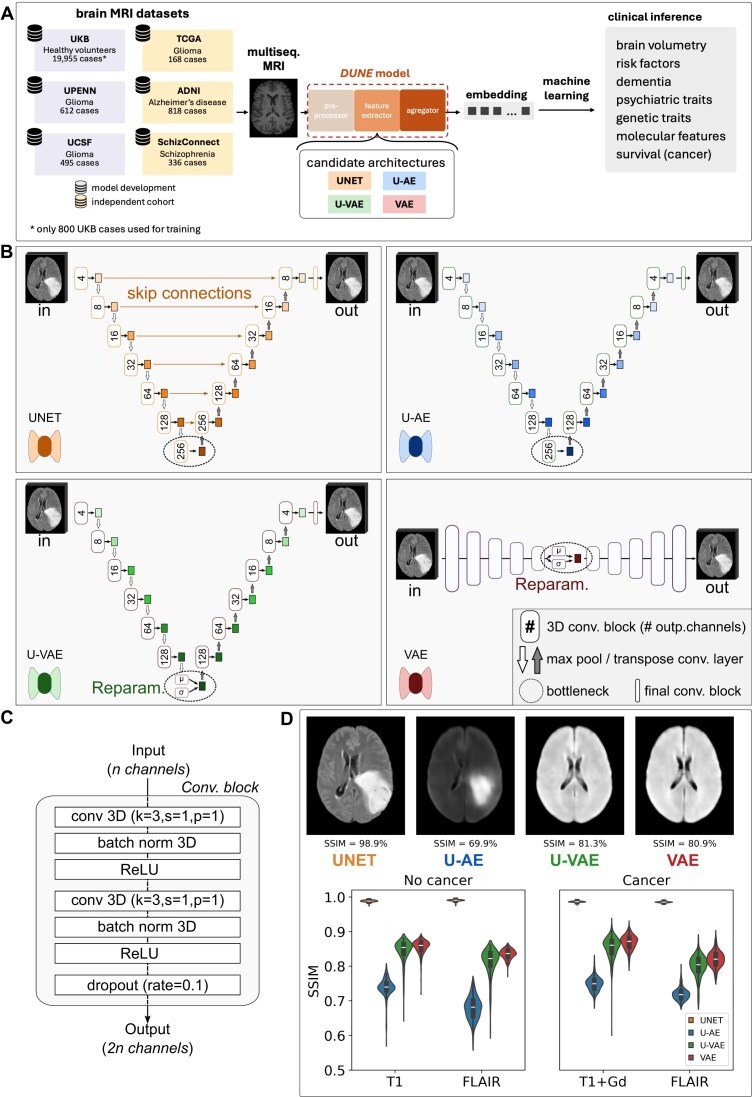
Project overview and model development. (A) Workflow for the development and evaluation of the *DUNE* model. The development of the model involved 3 distinct datasets: UKB, UPENN, and UCSF. The *DUNE* model trained to encode and reconstruct full-sized brain MRI scans, thereby generating embeddings that capture the salient information from the original images. Subsequently, embeddings were generated for scans from the development datasets as well as 3 independent cohorts (TCGA, ADNI, SchizConnect). These compact representations were then utilized as inputs to simple machine learning models for the inference of clinical variables, allowing for an assessment of the embeddings’ quality and their ability to facilitate downstream clinical applications. (B) We benchmarked 4 distinct candidate architectures for *DUNE*: UNET, U-AE, U-VAE, and VAE. Three of the models (UNET, U-AE, and U-VAE) are based on the U-Net architecture, while VAE is a variational autoencoder model adopted from a previous study [18]. Specifically, UNET is the foundational U-Net model, U-AE is a variant without skip connections, and U-VAE is a variational version of U-AE that generates embeddings through a reparameterization procedure. (C) The models are composed of sequential convolutional blocks and max pooling layers that reduce the dimension of brain MRI to generate the embeddings. (D) The base UNET model demonstrated the highest similarity (SSIM) between the output and original images. While the U-AE generated less precise images, it appeared to better identify cancer characteristics compared to variational autoencoders, which failed to reconstruct the tumor.

*DUNE* represents a workflow that transforms multisequence brain MRI data into standardized compact patient-level embeddings through integrated preprocessing, feature extraction, and aggregation, addressing MRI acquisition variability across institutions. An overview of the workflow for development and evaluation is presented in Fig. 1A.

### Datasets

Several datasets served for development and validation of *DUNE* (Fig. 1A, Supplemental Table S1). The model development was made using brain MRI scans from the UKB, UPENN, and UCSF datasets. The UKB (UK Biobank) dataset provides extensive clinical and radiological data for a large cohort of approximately 500,000 healthy individuals. Brain MRI (T1 and FLAIR sequences) and clinical data of 19,955 individuals (39,910 scans) were downloaded and preprocessed as described below. In addition to these healthy brains were included scans (T1 + gadolinium [T1Gd] and FLAIR) from the glioma cohorts UPENN (UPenn-GBM, 612 cases, 1,224 scans) and UCSF (UCSF-PDGM, 495 cases, 990 scans), to make the model equally capable of extracting features from morphologically abnormal brains.

For model evaluation and generalization assessment, 3 independent validation cohorts were utilized, representing distinct neurological conditions:

1. **Glioma validation cohorts**: The previously unseen TCGA-GBM and TCGA-LGG datasets (168 cases combined, 336 scans T1Gd and FLAIR) provided external validation for brain tumor applications.
2. **Alzheimer’s disease cohort**: Data from the ADNI database (818 cases; T1 scans only) enabled evaluation of neurodegenerative disease detection capabilities.
3. **Schizophrenia cohort**: The SchizConnect database, comprising COBRE and MCIC datasets (336 total cases; T1 scans only), allowed assessment of psychiatric disorder classification performance.

### Preprocessing

All the images were standardized through the preprocessing module of *DUNE* depicted in Supplemental Fig. S1, based on the Advanced Normalization Tools (ANTs) [19, 20]. When needed, DICOM files were converted to NIFTI files. Images of each sequence (T1, T1Gd, FLAIR) then underwent a series of transformations. Skull stripping was performed with the ANTS Brain extractor tool using templates and probability masks from the OASIS (Open Access Series of Imaging Studies) datasets [21]. The N4 bias field correction algorithm was applied, and FLAIR images were coregistered to T1. All images were then warped into a common space using the MNI-152 T1 1-mm template (Montreal Neurologic Institute [MNI] space) [22]. Pixel intensities were finally *z*-score normalized. The final dimensions (X, Y, Z) of the preprocessed images were [182, 218, 160] pixels (1 px/mm, 1 grayscale channel). This standardized preprocessing pipeline ensured consistent image quality and spatial alignment across all datasets, enabling effective multi-institutional model training while maintaining compatibility with different acquisition protocols and scanner types.

For the UKB dataset specifically, clinical variables from the whole dataset were collected, excluding those with a missing rate ≥15%. The data were standardized and data imputation was performed using the k-nearest neighbors algorithm (k = 140).

### Feature extractor architecture

Four candidate architectures were systematically benchmarked to identify the optimal feature extractor. Guided by previous research [23], we focused our investigation on unsupervised autoencoders, given their potential for learning efficient and versatile embeddings. The architectures of these competing models are depicted in Fig. 1B, C and detailed below.

We utilized autoencoders based on the UNet architecture, given their previous successes in medical images processing [24, 25]. The UNet framework was adapted for 3-dimensional (3D) image processing to handle volumetric brain MRI data effectively. The 4 architectures evaluated were the following:

1. **UNET**: A standard 3D U-Net autoencoder with skip connections. The first model was a “vanilla” 3D UNet autoencoder (UNET). This architecture maintains the traditional U-Net design with skip connections that allow information to bypass the bottleneck layer during reconstruction.
2. **U-AE**: A U-Net autoencoder without skip connections. Second, we reasoned that the UNet skip connections, while helpful in supervised segmentation tasks [24], could be detrimental to the embedding quality in our application. We therefore derived a version of the UNET model without skip connections (U-AE). This modification forces all information through the bottleneck layer, potentially creating more compressed and informative features.
3. **U-VAE**: A variational version of the U-AE model with reparameterization layer. Third, given published data suggesting that variational autoencoders can be useful for magnetic resonance (MR) feature extraction [26, 27], we designed a variational version of the U-AE model (U-VAE) by including a reparameterization layer in the bottleneck section.
4. **VAE**: A fully connected variational autoencoder. Finally, we used a fully connected variational autoencoder (VAE), which successfully extracted features from computed tomography images of lung lesions in a previous study [18]. This architecture provides a baseline comparison using a non-UNet-based approach.

The encoder and decoder components were built using convolutional blocks, where each block repeated twice the following sequence: a 3D convolution layer (kernel = 3, stride = 1, padding = 1), followed by batch normalization and ReLU activation (Fig. 1C). Between these convolutional blocks, max pooling layers were used in the encoder while transposed convolution layers (kernel = 2, stride = 2, padding = 0) were used in the decoder. As the input images progressed through the encoder, their dimensions were progressively reduced while the number of channels expanded in consecutive blocks (4, 8, 16, 32, 64, and 128). The bottleneck architecture differed between conventional and variational autoencoders: conventional autoencoders used a single convolutional block, while variational autoencoders employed 2 separate convolutional blocks followed by a reparameterization step. Each autoencoder generated 2 outputs: a vector of low-dimensional features at the bottleneck and a reconstructed version of the input image. We set the number of encoder/decoder blocks to 6, which yielded between 1,000 and 5,000 features per input image for conventional autoencoders and exactly 2,048 features for variational autoencoders, dimensions suitable for subsequent machine learning tasks.

### Autoencoder training algorithm

All deep learning models were implemented using the PyTorch library (version 2.0.0).

To prevent overfitting the model to normal healthy brains, only 800 randomly selected cases were finally kept from the UKB cohort for the model development, corresponding to 1,600 scans (T1 and FLAIR sequences). This strategic reduction from the full UKB cohort (19,955 individuals) was crucial to maintain balanced representation between healthy and pathological brain morphologies. Combined with glioma scans from UPENN (1,224 scans) and UCSF (990 scans), the final training dataset comprised 3,814 scans (T1, T1Gd, FLAIR; 80% training, 20% validation).

The training was carried out with a batch size of 14 images. For each batch, we aimed for the autoencoders to encode and reconstruct images as close to the original as possible. Therefore, we opted for a loss function relying on the structural similarity index measure (SSIM) between the input (x) and the output (ŷ) produced by the autoencoders [28], defined as follows (1):


(1) \begin{eqnarray*}
{\mathrm{SSIM}}(x,y) = \frac{{(2{\mu }_x{\mu }_y + {C}_1)(2{\sigma }_{xy} + {C}_2)}}{{\left( {\mu _x^2 + \mu _y^2 + {C}_1} \right)\left( {\sigma _x^2 + \sigma _y^2 + C2} \right)}}
\end{eqnarray*}

with (μx, σx²) and (μŷ, σŷ²) the respective pixel sample mean and variance of the input (x) and output (ŷ) images, σxŷ the covariance of x and ŷ, and C₁ and C₂ two variables to stabilize divisions with weak denominators. As the SSIM ranges from 0 to 1, the autoencoder’s objective is thus to minimize the following loss function (2):


(2) \begin{eqnarray*}
{\mathrm{SSI}}{{\mathrm{M}}}_{{\mathrm{loss}}} = \frac{1}{{\mathrm{n}}}\sum\limits_{{\mathrm{i}} = 1}^{\mathrm{n}} {1 - {\mathrm{SSIM}}({{\mathrm{x}}}_{\mathrm{i}},{{\mathrm{y}}}_{\mathrm{i}})}
\end{eqnarray*}

For variational autoencoders, the Kullback–Leibler divergence (KLD) loss (3) of the embedding distribution—which quantifies its divergence with the Gaussian distribution—was added to the SSIM loss (4), with a weighting β parameter set to 10^−4^ [29, 30]. The ADAM algorithm was used as the optimizer, with a learning rate of 10^–4^.


(3) \begin{eqnarray*}
{\mathrm{KL}}{{\mathrm{D}}}_{{\mathrm{loss}}} = \sum\limits_{{\mathrm{i}} = 1}^{\mathrm{n}} {\sigma _{\mathrm{i}}^2 + \mu _{\mathrm{i}}^2 - {\mathrm{log}}({\sigma }_{\mathrm{i}}) - 1}
\end{eqnarray*}

(4) \begin{eqnarray*}
\mathcal{L} = {\mathrm{SSI}}{{\mathrm{M}}}_{{\mathrm{loss}}} + {\mathrm{\beta KL}}{{\mathrm{D}}}_{{\mathrm{loss}}}
\end{eqnarray*}

Training was performed until convergence, with early stopping implemented to prevent overfitting.

### Patient-level embedding generation

For each input MRI sequence (T1, T1Gd, or FLAIR), numerical characteristics (referred to as “features” throughout this article) were extracted from the autoencoder’s bottleneck layer. These sequence-specific features were then concatenated to generate comprehensive patient-level representations (referred to as “embeddings”), creating unified patient signatures that capture information from multiple MRI sequences while maintaining the distinct characteristics encoded by each sequence type.

To benchmark our approach, we also extracted radiomics features (excluding diagnostic features) from each sequence using the *PyRadiomics* library (v3.1.0) with whole brain masks for segmentation [31]. Radiomics served as an established reference method for medical image feature extraction, providing baseline comparison for evaluating the effectiveness of our deep learning–based approach. The radiomics features (1,132 per scan) were concatenated at the patient level following the same methodology as autoencoder features, enabling direct comparison between both feature extraction methods.

### Statistical analysis and embedding evaluation

The quality and clinical relevance of the extracted embeddings were assessed through a comprehensive evaluation framework combining exploratory analyses, predictive modeling, and statistical comparisons.

#### Exploratory analysis

UMAP analyses were computed on embeddings (2 components, 15 neighbors). Canonical correlations analysis (CCA) was performed between embeddings and clinical variables, which were summarized to 2 main components.

#### Predictive modeling

Machine learning models were trained to predict clinical variables using the different generated embeddings. The model architecture was selected based on the type of clinical variable: ridge regression for quantitative variables, L2-penalized logistic regression for categorical variables, and random survival forests for survival variables.

For each clinical variable, separate models were trained using embeddings from each autoencoder architecture and radiomics. We employed a 5-fold cross-validation procedure, with oversampling to address class imbalance in categorical variables.

#### Performance evaluation

Hyperparameter optimization was performed using GridSearchCV within each fold to ensure optimal model configurations for each type of embedding.

The predictions from all validation folds were concatenated to generate receiver operating characteristic (ROC) curves and compute performance metrics: weighted F1-scores for categorical variables, *R*² scores for quantitative variables, and C-indexes and Integrated Brier Scores for survival variables.

The predictive performance of models using different embeddings was compared using Wilcoxon signed-rank tests with Bonferroni correction for multiple comparisons.

Analyses were carried out with the scikit-learn library.

### Synthetic data generation

To address datasets with limited sequence availability, we implemented a synthetic data enhancement strategy. A dedicated UNet-based model was trained to perform bidirectional synthesis between T1 and FLAIR sequences.

The synthetic data generation model utilized a standard 3D U-Net autoencoder architecture (with skip connections). The model consisted of 6 encoder/decoder blocks with progressive feature expansion (4, 8, 16, 32, 64, and 128 channels), processing single-channel inputs (T1 or FLAIR sequences independently). Each convolutional block comprised 2 sequential 3D convolution layers (kernel = 3, stride = 1, padding = 1), followed by batch normalization, ReLU activation, and dropout (rate = 0.1). Max pooling layers (2 × 2 × 2) were used for downsampling in the encoder, while transposed convolution layers (kernel = 2, stride = 2, padding = 0) performed upsampling in the decoder.

The model was trained on the UKB dataset using 39,910 sequences (T1 and FLAIR pairs) with an 80/20 train/validation split, to establish bidirectional correspondence between T1 and FLAIR sequences, enabling the model to learn both T1→FLAIR and FLAIR→T1 synthesis. The training objective utilized SSIM loss to optimize structural similarity between synthethic and corresponding real images, with the ADAM optimizer and learning rate of 5 × 10^−5^.

## Results

### Model reconstruction performance comparison

We compared the reconstruction capabilities of the 4 candidate architectures for *DUNE*’s feature extraction module. All 4 models trained to encode and reconstruct single-sequence 3D brain MRI scans (Fig. 1D). Reconstruction performance varied significantly across architectures (Fig. 1D). UNET achieved the highest SSIM scores (98.7% ± 4.2%) across all sequences (T1, T1Gd, FLAIR) for both cancer and noncancer images. U-VAE and VAE achieved SSIM scores of 82.6% ± 4.5% and 84.4% ± 2.8% respectively, for general reconstruction but showed poor performance on brain tumor reconstruction. U-AE achieved SSIM scores of 72.2% ± 4.2%.

However, since our primary objective was to generate clinically meaningful embeddings rather than achieve optimal reconstruction, we next evaluated the quality of bottleneck-derived features for downstream clinical prediction tasks.

### MRI embeddings accurately predict clinical phenotypes of healthy individuals

#### Clinical variables and study design

**Figure 2:**
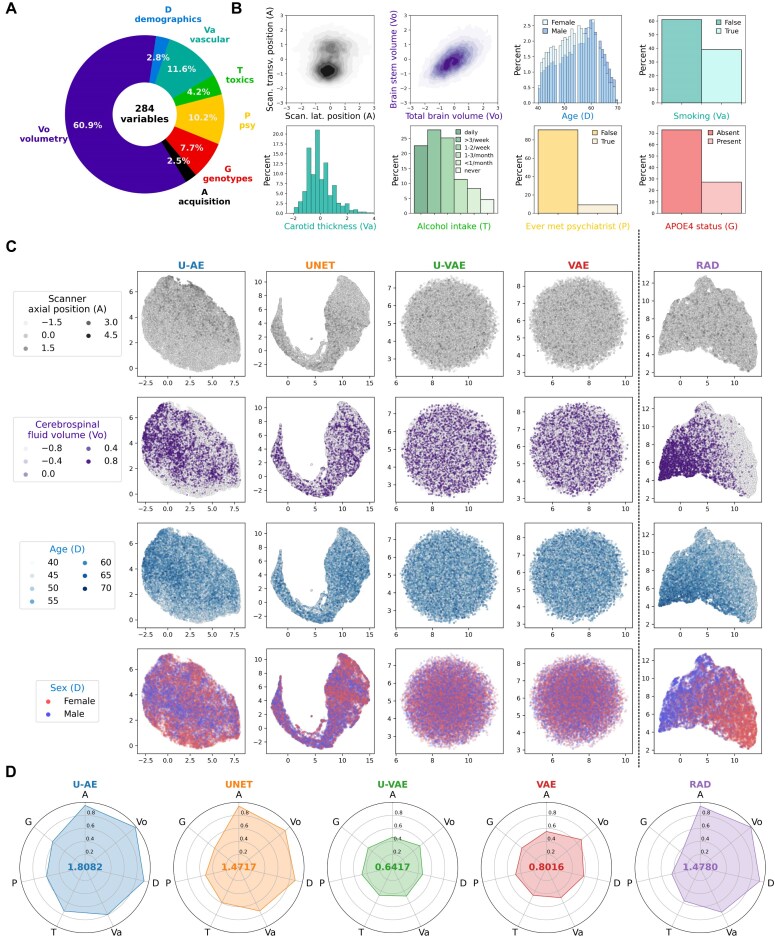
MRI embeddings correlate with clinical phenotypes in healthy individuals. (A) In total, 284 clinical parameters collected in the UKB dataset were divided into 7 categories (Vo: volumetry, A: acquisition protocol, G: genotypes, P: psychiatric traits, T: toxics, Va: vascular conditions, D: demographics). (B) Distribution of illustrative variables from the different categories. (C) UMAP reduction was performed on embeddings extracted from brain MR images by the 4 models and on radiomics features as control. Radiomics-, UNET-, and U-AE–generated embeddings successfully capture variables of acquisition, volumetry, and demographic categories, while those issued by variational autoencoders do not. (D) Canonical correlation analysis (CCA) between embeddings and clinical variables from each category. The radar plots display the correlation (*R*^2^ metric) between the first canonical variate of each category and that of each embedding, showing that U-AE embeddings correlate best with UKB clinical parameters.

To identify the optimal architecture for DUNE among the 4 candidates, we conducted a comprehensive evaluation based on the quality of the embeddings they produce. As a first assessment criterion, we measured how well these embeddings correlated with clinical variables (Fig. 2). We used all 4 candidate models to encode the images from the UKB dataset, with radiomics serving as a control given its established role as a state-of-the-art method for image feature extraction [31]. For each patient, we generated global brain MR embeddings by concatenating features extracted from both T1 and FLAIR sequences.

We analyzed these embeddings against 284 clinical variables hypothesized to be associated with brain morphology. These variables were categorized into 7 distinct groups: acquisition parameters (A), brain volumetry (Vo), demographics (D), vascular risk factors (Va), toxics (T), psychiatric (P), and genetic (G) traits (Fig. 2A, B). The variables showed diverse statistical distributions, with some quantitative variables such as volumetric measurements, age, and carotid thickness following normal distributions (Fig. 2B), while certain qualitative variables, notably APOE4 status, exhibited significant class imbalance. Our correlation analyses revealed strong associations within each category but weak correlations between categories (Supplemental Fig. S2), leading us to analyze each group independently.

#### Exploratory analysis

UMAP analyses of the embeddings revealed distinct patterns (Fig. 2C). The variational autoencoders (U-VAE and VAE) produced normally distributed embeddings, as expected given their underlying statistical constraints. Embeddings from radiomics, U-AE, and, to a lesser extent, UNET showed clear relationships with key clinical parameters, evidenced by distinct color patterns corresponding to acquisition parameters (scanner table position), volumetric measurements (cerebrospinal fluid volume), and demographic features (age and sex). In contrast, the embeddings from VAEs showed no discernible patterns in relation to these clinical variables.

To assess the overall relationship between embeddings and clinical variables, we performed CCA on each clinical variable group. Figure 2D displays the determination coefficients (*R*² scores) between the first canonical variates of the embeddings and that of each subgroup of clinical variables. The total area covered by these coefficients in the radar plot summarizes the overall correlation strength. Using this metric, U-AE embeddings demonstrated the strongest correlation with clinical variables (area = 1.81), outperforming the radiomics features (area = 1.48), which ranked second.

#### Clinical prediction tasks

**Figure 3:**
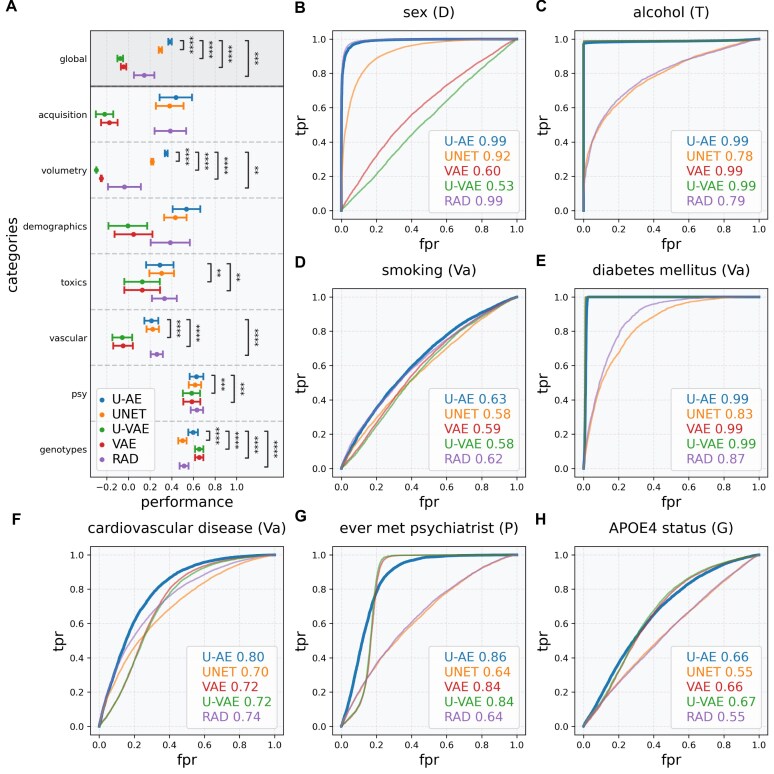
Clinical inference using MRI embeddings of healthy brains. (A) Performance of machine learning models trained to predict clinical variables using model-generated embeddings of the UKB brain MRIs (T1 and FLAIR sequences, 19,955 cases, 5-fold cross-validation). Points and error bars show means and standard errors of prediction scores (*R*^2^ or F1-scores) across variables in each category. Overall, U-AE embeddings yielded the best predictions, except for toxics, genotypes, and vascular conditions that were best predicted by radiomics and variational autoencoders, respectively. (B–H) Individual performance of clinical inference based on either autoencoder embeddings or radiomics.

As a second evaluation criterion, we assessed how well each type of embedding could serve as input features for clinical prediction tasks. To do this, we trained supervised machine learning models to predict each of the 284 clinical variables from the UKB dataset. For each variable, models were trained on embeddings from each architecture (UNET, U-AE, U-VAE, and VAE) or radiomics (RAD), used as control. The performance of clinical prediction was measured by the *R*^2^ score (quantitative variables) or the weighted F1-score (categorical variables). The results are displayed in Fig. 3A (overall performance and per variable subgroups).

Clinical prediction performance varied across embedding types (Fig. 3A). U-AE embeddings achieved prediction scores of 0.384% ± 1.63%, compared to radiomics (0.145% ± 9.7%, *P* < 0.001). UNET, U-VAE, and VAE embeddings achieved lower prediction scores across clinical variables. The performances greatly varied between the different subgroups of clinical variables but showed small variation across variables within each category (Fig. 3A), which tended to be highly intercorrelated (Supplemental Fig. S2). Figure 3B–H thus displays the performance of model predictions for illustrative variables from each category. U-AE embeddings were the most relevant in predicting most clinical variables. Embeddings encoded by variational autoencoders (U-VAE and VAE) were outperformed in all categories, except genetics.

Based on these comprehensive evaluations, U-AE was identified as the optimal feature extraction architecture for DUNE in morphologically normal brains, demonstrating superior embedding quality despite its poor sequence-level reconstruction performance.

### MRI embeddings capture cancer-specific features, including molecular features, and predict patient survival

Having validated our models on healthy brains, we next investigated their generalization capabilities to pathological cases. Specifically, we first focused on glioma patients, whose brain MRIs exhibit significant morphological abnormalities due to tumor presence. For this analysis, we used 2 categories of datasets: the UCSF and UPENN datasets, which were part of the autoencoder training set, along with the previously unseen TCGA-GBM and TCGA-LGG datasets. Once more, we assessed the quality of the embeddings in oncological context by developing survival prediction models, using them as input. For evaluation, we stratified the patients into 2 groups based on their predicted mortality risk (above or below median) and compared their survival curves. The prediction quality was measured by 2 complementary metrics: the Brier score, which measures prediction accuracy (optimal when close to 0), and the concordance index (C-index), which measures ranking accuracy (optimal when close to 1).

**Figure 4:**
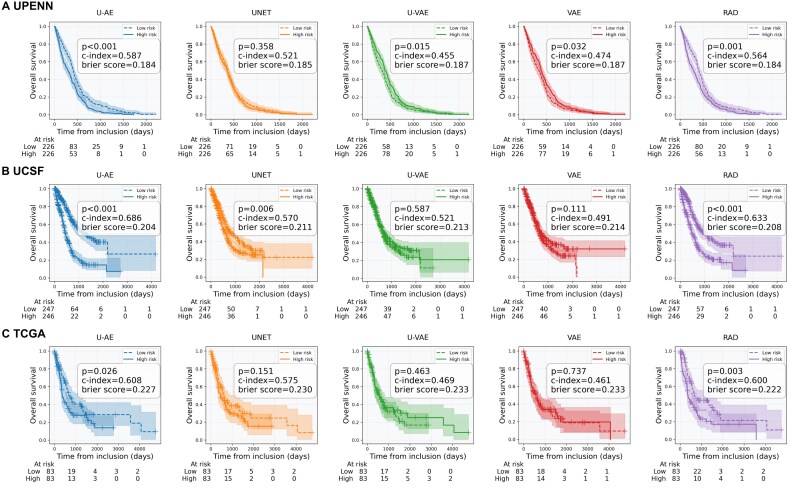
MRI embeddings allow for survival inference in individuals with glioma. Performance of random forest survival models predicting patient global survival based on MR embeddings/radiomics (T1Gd and FLAIR sequences) of 3 glioma datasets (5-fold cross-validation). The overall survival of 2 groups, defined by risk scores of death below (low risk) or above (high risk) the median, was compared (concordance index and log-rank test) for the UPENN (A) and UCSF (B) datasets (which were used as train sets for the autoencoders) and for the external TCGA (C) dataset. Predictions using the U-AE embeddings had the highest concordance index for all 3 datasets.

Models using U-AE encoded embeddings demonstrated superior performance in discriminating between low- and high-risk patients. This superiority was consistent across both internal validation datasets, with C-index = 0.587 and Brier score = 0.184 for UPENN and C-index = 0.686 and Brier score = 0.204 for UCSF, outperforming both radiomics features and alternative encodings from UNET and VAE architectures (Fig. 4A, B). The external validation on the TCGA dataset confirmed these findings, with U-AE embeddings achieving better survival predictions (C-index = 0.608) compared to radiomics (C-index = 0.600) and other autoencoder-based approaches (Fig. 4C).

**Figure 5:**
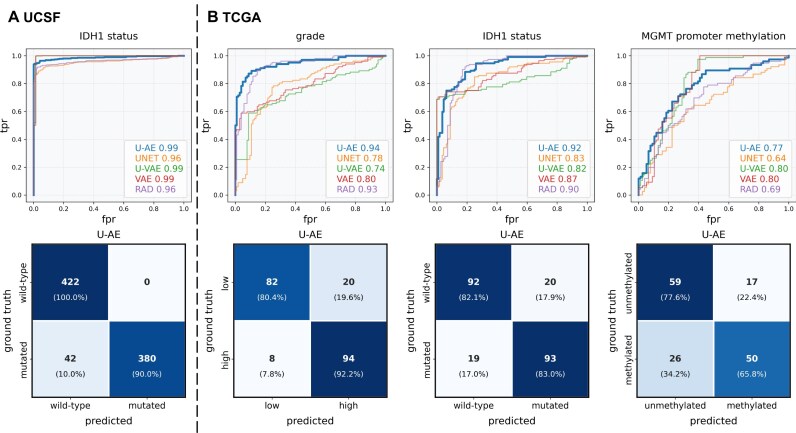
MRI embeddings enable inference of molecular features in patients with glioma. Performance of models predicting molecular features using MR embeddings/radiomics (T1Gd and FLAIR sequences) of the UCSF (A, IDH1 status) and TCGA datasets (B, cancer grade, IDH1 status, MGMT promoter methylation status).

Beyond survival prediction, we evaluated the embeddings’ ability to predict key molecular characteristics of gliomas that guide therapeutic decisions. These included IDH1 mutation status and MGMT promoter methylation status, which are established markers of response to chemoradiation and temozolomide-based chemotherapies (Fig. 5A, B). The U-AE embeddings consistently demonstrated superior predictive performance, achieving high accuracy in detecting IDH1 mutations (area under the receiver operating characteristic curve [AUROC] = 0.99 in UCSF and AUROC = 0.92 in TCGA), MGMT promoter methylation status (AUROC = 0.77), and tumor grade (AUROC = 0.94). Notably, even in these morphologically complex cases, the embeddings maintained their ability to predict general anatomical features such as patient sex (data not shown).

### MRI embeddings predict diagnosis of neurodegenerative and psychiatric disorders

We continued the benchmarking of the different models with 2 additional neurological conditions: neurodegenerative disease and schizophrenia. For this analysis, we leveraged data from the ADNI database for Alzheimer’s disease and the SchizConnect database (comprising COBRE and MCIC datasets). Unlike our previous analyses, these datasets contained only T1-weighted images, allowing us to test our models’ performance with limited imaging sequences.

**Figure 6:**
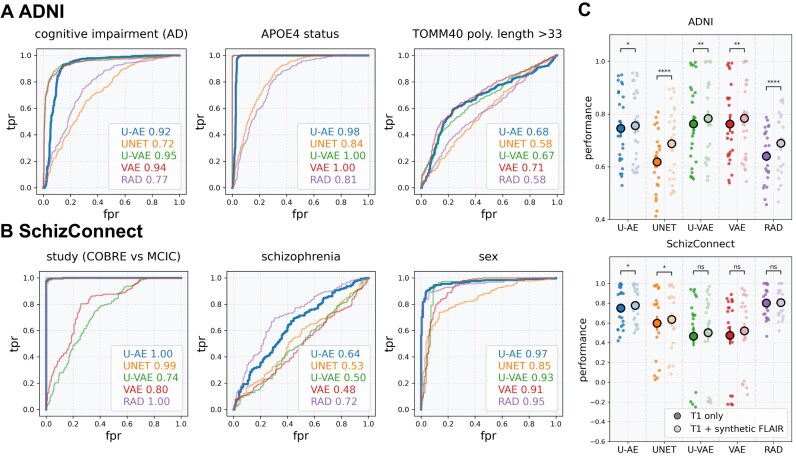
Clinical inference in Alzheimer’s disease and schizophrenia. (A) Performance of models predicting clinical features (cognitive impairment as defined by mini-mental state examination [MMSE >28]) and genotypes (APOE4 status and length of the TOMM40 poly-T polymorphism [>33]) using MR embeddings/radiomics of the ADNI dataset (T1 sequences). (B) Performance of models predicting parameters and clinical variables (cohort of origin [COBRE vs. MCIC], schizophrenia vs. healthy patients, sex and age) using MR embeddings/radiomics of the SchizConnect dataset (T1 sequences). (C) Performance of models predicting clinical parameters from ADNI (top) and SchizConnect (bottom) datasets based on MR embeddings/radiomics of either T1-only images (dark dots) or both T1 + synthetic FLAIR images (light dots).

In the Alzheimer's disease cohort, we first evaluated the models’ ability to predict cognitive impairment severity. The U-AE embeddings demonstrated robust performance with an AUROC of 0.92 (Fig. 6A). We then evaluated the prediction of genetic markers known to influence Alzheimer’s disease progression: the APOE4 allele status and the TOMM40 poly-T polymorphism length (dichotomized at the cohort median of 33). Interestingly, for these genetic predictions, the variational autoencoders performed the best. The U-VAE and VAE embeddings achieved perfect prediction for the APOE4 allele (AUROC = 1) and moderately high accuracy for TOMM40 polymorphism length (AUROC = 0.67 and 0.71, respectively), compared to the U-AE embeddings (AUROC = 0.98 for APOE4 and AUROC = 0.68 for TOMM40) (Fig. 6A).

In the schizophrenia cohort, we assessed the models’ performance across 4 distinct prediction tasks: identification of study origin (COBRE vs. MCIC, which reflected differences in MR acquisition protocols), clinical diagnosis (schizophrenia vs. healthy volunteers), and demographic characteristics (sex and age). The U-AE embeddings maintained their superior performance, perfectly discriminating between studies of origin (AUROC = 1) and accurately predicting patient sex (AUROC = 0.97). For schizophrenia diagnosis, both U-AE embeddings and radiomics features achieved meaningful predictive performance (AUROC = 0.64 and 0.72, respectively) (Fig. 6B).

### MRI embeddings can be improved by synthetic data

To address the limitation of single-sequence availability in the ADNI and SchizConnect datasets, we developed an enhancement strategy using synthetic data generation. We applied a synthetic FLAIR generation model to both the SchizConnect and ADNI datasets, generating FLAIR sequences from their available T1 images. This approach proved highly effective, with synthetic FLAIR images showing strong concordance with real FLAIR images in the UKB validation set (similarity index = 0.90 ± 0.04). We then applied this synthetic data enhancement to both the SchizConnect and ADNI datasets, generating FLAIR sequences from their available T1 images. This strategy enabled us to compare the predictive performance between embeddings derived from T1 images alone and those generated from the combination of T1 and synthetic FLAIR images, using our established clinical parameters.

The integration of synthetic FLAIR images yielded consistent improvements in prediction performance across all analyses. In the ADNI dataset, embeddings combining T1 and synthetic FLAIR sequences demonstrated enhanced predictive capability compared to those derived from T1 sequences alone (0.756 ± 0.14 vs. 0.745 ± 0.14 with U-AE embeddings; Fig. 6C, top). We observed similar improvements in the SchizConnect dataset, where the addition of synthetic FLAIR sequences enhanced the predictive performance of the embeddings (0.777 ± 18.9 vs. 0.750 ± 0.21 with U-AE embeddings; Fig. 6C, bottom). Remarkably, this enhancement in performance was consistent across all autoencoder architectures, suggesting that the benefit of incorporating synthetic FLAIR sequences extends beyond any specific model implementation.

## Discussion

We introduce *DUNE*, a complete workflow that generates versatile and comprehensive embeddings from brain MRI scans for downstream analyses. DUNE’s feature extractor was developed through systematic benchmarking of 4 autoencoder architectures. By evaluating these relatively simple architectures on diverse prediction tasks, we identified that a skip-connection-free UNet autoencoder (U-AE) consistently generates the most informative embeddings from brain MRIs and was therefore selected as the final architecture for DUNE. Surprisingly, this architecture demonstrated the poorest reconstruction capabilities while producing the most clinically relevant embeddings, highlighting a counterintuitive finding that reconstruction performance does not predict embedding quality for clinical applications. We further validated this principle through additional experiments with enhanced loss functions incorporating gradient-based detail preservation constraints, which confirmed improved reconstruction quality at the cost of reduced clinical prediction performance (data not shown). This trade-off represents an important consideration in autoencoder architectures, where the removal of skip connections forces all information through the bottleneck layer, creating more informative representations despite reduced pixel-level accuracy.

The versatility of DUNE’s embeddings was demonstrated across multiple levels of clinical prediction. When used with simple machine learning models, they enabled accurate prediction of basic parameters such as brain volumetry and demographic features (age, sex), while simultaneously capturing complex molecular and genetic signatures. In neuro-oncology, they achieved robust prediction of IDH1 mutation status, matching the performance of sophisticated deep learning models that process whole MR images directly [49–51]. In neurodegenerative diseases, they successfully identified genetic traits such as APOE4 allele status, and in psychiatric disorders, they effectively detected subtle brain alterations associated with disease status.

Among the candidate architectures we evaluated, UNet-based autoencoders were initially selected for their relative simplicity and proven success in medical image processing [52]. We hypothesized that such models could generate embeddings that faithfully capture the fundamental features of brain MRI structures. Unlike conventional applications, we employed unsupervised training through image reconstruction to optimize embedding versatility. Our systematic evaluation revealed that while the standard UNet architecture excelled at image reconstruction, its skip connections, which allow information to bypass the bottleneck layer, resulted in suboptimal embeddings. The removal of these skip connections in our U-AE model forced all information through the bottleneck, producing more informative embeddings despite lower reconstruction quality. We also explored variational autoencoders based on previous work [23, 53–55], but these were outperformed by traditional architectures and even radiomics, despite careful tuning of the Kullback–Leibler divergence term in the loss function.

Recent advances in computer vision have highlighted vision transformers as powerful tools for image analysis [56, 57, 58, 59]. Their self-attention mechanisms enable sophisticated spatial reasoning by weighing the importance of different image regions, which could theoretically benefit brain MRI analysis (1–5). However, several considerations led us to favor autoencoders for this application. First, while vision transformers excel at classification tasks, they are not inherently designed for dimensionality reduction and feature extraction, which was our primary objective. Second, the traditional autoencoder architecture provides an interpretable bottleneck structure that naturally aligns with our goal of generating compact, meaningful embeddings. Third, vision transformers typically require extensive pretraining on large-scale datasets to achieve optimal performance, making them less suitable for specialized medical imaging applications where data availability is limited. For 3D brain MRI analysis specifically, the computational and data requirements of transformer architectures become prohibitive given the high complexity of volumetric data and the relatively modest size of available medical imaging datasets. Finally, autoencoders offer better computational efficiency and have demonstrated superior adaptability to smaller, domain-specific datasets [60]. This latter point was particularly crucial for our application, as it enabled us to train effectively on our relatively modest collection of brain MRI scans while maintaining model stability and generalization capability.

A major challenge in developing MRI analysis models is the heterogeneous nature of image acquisition across different centers. This heterogeneity makes image normalization particularly challenging and often leads to models that overfit to their training dataset and generalize poorly to external data. We addressed this challenge through 2 key strategies. First, we implemented a comprehensive image standardization pipeline, enabling our models to effectively learn from multiple datasets while maintaining robustness to external data. Second, and more importantly, we identified that maintaining a balanced representation of different brain morphologies in the training data was crucial. Initially, training with the complete UKB cohort (≈20,000 cases) alongside the glioma datasets led to a bias toward normal brain morphology, resulting in poor-quality embeddings for tumor cases. We resolved this issue by downsampling the UKB dataset to match the size of the glioma datasets, which improved the quality of tumor embeddings while preserving the model’s performance on normal brain images (data not shown).

The processing of multiple MRI sequences represented another key optimization challenge in our model development. Initially, we explored a multichannel approach where different MR sequences were concatenated and processed simultaneously, similar to how RGB channels are handled in natural image processing. However, our systematic evaluation revealed superior performance with an alternative strategy: processing each MR sequence independently through separate encoding paths and subsequently concatenating their extracted features. This sequential approach not only improved the quality of the extracted features but also provided greater operational flexibility, allowing the model to handle incomplete imaging datasets where certain sequences might be unavailable. Such flexibility is particularly valuable in clinical settings, where the availability of multiple MRI sequences cannot always be guaranteed.

Our exploration of synthetic data enhancement revealed promising opportunities for improving embedding quality. We explored several strategies to enhance the quality of our embeddings through data augmentation. Our initial attempt to combine autoencoder-generated embeddings with radiomics features showed no improvement in downstream clinical predictions compared to either approach alone (data not shown). However, we discovered that incorporating synthetic MRI sequences, generated through deep learning, significantly enhanced embedding quality.

The success of synthetic data enhancement likely stems from the complementary information encoded across MRI sequences. T1-weighted images primarily capture anatomical structures and tissue contrast, while FLAIR sequences enhance visualization of pathological changes and cerebrospinal fluid suppression. By generating the missing modality, the multimodal nature of embeddings was restored, enabling single-sequence datasets to leverage the enhanced representational capacity demonstrated by dual-sequence embeddings in our initial validation.

This finding is particularly relevant in the current context of medical imaging, where there is increasing pressure to optimize MRI protocols by reducing acquisition times and sequences. Our results suggest that synthetic imaging could help maintain comprehensive analyses while limiting the actual scanning time. Moreover, this success with synthetic MRI data opens promising perspectives in the broader context of generative AI. Similar approaches could be developed to generate synthetic data across other modalities such as transcriptomics or pathology images [61–64, 17]. Such synthetic data generation could provide a powerful strategy to enhance the quality of medical imaging embeddings by incorporating complementary information from multiple modalities, even when the original data are not available.

Literature is scarce regarding models that allow unsupervised feature extraction from brain MRI, with rare applications in cancer or psychiatric disorders [65, 66]. Therefore, radiomics—first coined by Lambin et al. [67] as an innovative algorithm, allowing the high-throughput extraction of medical images—has remained the best way to extract features from radiological images thus far. The radiomics workflow includes a segmentation step, followed by the extraction of multiple quantitative features consisting of intensity distribution, spatial relationships between intensity levels, texture patterns, and shape descriptors. The most informative of these features are automatically selected from specific criteria and can further be used in downstream tasks. The robustness of radiomics makes it applicable to multiple imaging modalities in a broad range of applications, particularly in cancer [68–70]. In contrast to radiomics, which extract quantitatively measurable features, deep learning models extract more abstract and higher-level features that are expected to generate more comprehensive embeddings. Indeed, in the current study, the embeddings generated by our model generated better predictions than that based on radiomics.

The potential applications of DUNE extend to the emerging field of data fusion, where features from multiple data sources are combined to enhance prediction accuracy. Recent studies have demonstrated the power of this approach, showing how the integration of pathology and transcriptomics data can improve brain cancer prognosis prediction beyond what either modality can achieve alone [71]. In this context, DUNE could provide a robust framework for incorporating radiological data into such multimodal analyses, potentially further improving predictive performance.

While our results demonstrate DUNE’s broad utility, several limitations should be acknowledged. Currently, the model is optimized for 3 specific MRI sequences: T1, T1Gd, and FLAIR. Although additional sequences, such as diffusion-weighted imaging, could be incorporated given sufficient training data, this represents a current constraint on the model’s applicability. Furthermore, unlike the more versatile radiomics approach, which can analyze any radiological image, DUNE is specifically designed for brain MRI analysis. However, the underlying architecture is fundamentally capable of processing any 3D medical image, suggesting that organ-specific versions could be developed for other clinical applications.

Another potential limitation is the risk for dataset-specific confounding, as certain diseases are primarily represented within specific institutional cohorts, potentially leading to embeddings capturing technical rather than pathological characteristics. However, we implemented strategies to mitigate this risk, and multiple lines of evidence suggest that DUNE embeddings capture biological rather than technical features. First, we employed the ANT-based preprocessing pipeline, which harmonizes images across different acquisition protocols and scanner types, thus reducing technical variability. Additionally, DUNE successfully predicts fundamental biological features (e.g., sex, IDH1 or APOE4 status) across different datasets and demonstrates robust performance on external validation cohorts acquired using different protocols than training datasets (TCGA, ADNI, SchizConnect). If embeddings primarily encoded scanner-specific artifacts, we would expect degraded performance on these cross-institutional predictions, which we do not observe.

In conclusion, DUNE represents a significant advance in medical image analysis, capable of extracting comprehensive compact representations from complex brain MRI scans. These embeddings effectively capture both obvious and subtle imaging features while maintaining clinical relevance. By facilitating more accurate diagnoses, refined prognostic assessments, and better-informed therapeutic decisions, DUNE contributes to the advancement of precision medicine in neurology. The model’s ability to generate meaningful embeddings from standard clinical imaging sequences, combined with its potential for integration into multimodal analysis frameworks, positions it as a valuable tool for developing more personalized and effective patient care strategies.

## Availability of Source Code and Requirements

Project name: DUNE

Project homepage: https://github.com/gevaertlab/DUNE

License: Apache-2.0 license

SciCrunch RRID:SCR_027208

bio.tools ID: GevaertLab_DUNE

System requirements

Operating system: Platform independent

Programming language: Python

Package management: pip

Hardware requirements: 16 GB+ RAM, GPU with 16 GB+ VRAM, CPU multicore processor (8+ cores recommended)

## Supplementary Material

giaf116_Supplemental_Files

giaf116_Authors_Response_To_Reviewer_Comments_Original_Submission

giaf116_GIGA-D-25-00076_Original_Submission

giaf116_GIGA-D-25-00076_Revision_1

giaf116_Reviewer_1_Report_Original_Submission Laura Caquelin -- 4/14/2025

giaf116_Reviewer_2_Report_Original_Submission Huaiqiang Sun -- 4/22/2025

giaf116_Reviewer_2_Report_Revision_1 Huaiqiang Sun -- 9/3/2025

giaf116_Reviewer_3_Report_Original_Submission Piotr M. Szczypinski -- 5/6/2025

giaf116_Reviewer_3_Report_Revision_1 Piotr M. Szczypinski -- 8/9/2025

## Data Availability

Clinical and imaging data (DICOM and NIFTI files) were collected from publicly available datasets: the UKB (data use agreement, Application Number 51,998) healthy volunteer dataset [32, 33], the MCIC and COBRE schizophrenia datasets [34–37], and the ADNI Alzheimer dataset. Glioma datasets were downloaded from The Cancer Imaging Archive [38, 39]: UCSF-PDGM [40], UPenn-GBM [41, 42], TCGA-LGG [43], and TCGA-GBM [44, 45].ADNI data access followed standard protocols through the ADNI database [46]. Glioma datasets from The Cancer Imaging Archive were accessed according to their respective data use policies.The source codes of the different autoencoders presented in this article are available on GitHub. The complete DUNE workflow is registered in WorkflowHub (doi:10.48546/workflowhub.workflow.1809.1), bio.tools (biotools:GevaertLab_DUNE), and SciCrunch (RRID:SCR_027208). All preprocessing scripts, model implementations, and evaluation code necessary to reproduce the main findings of this study are publicly available through the GitHub repository. The data and code required to reproduce the figures are available at the FigShare repository [47]. Processed embeddings and intermediate analysis results are available upon reasonable request to the corresponding author. All additional supporting data are available in the GigaScience repository, GigaDB [48].

## Additional Files

**Supplemental Table S1**. Brain MR datasets used in the study. Images of the UKB, UPENN, and UCSF datasets were used to train the models. HV: healthy volunteers; QA: quality assessment; T1Gd: T1 + gadolinium.

**Supplemental Fig. S1**. Brain MR preprocessing steps.

**Supplemental Fig. S2**. Correlation matrix of UKB clinical variables.

**Supplemental Fig. S3**. Generation of synthetic FLAIR from T1.

## Abbreviations

ADNI: Alzheimer’s Disease Neuroimaging Initiative; ANT: Advanced Normalization Tool; AUROC: area under the receiver operating characteristic curve; CCA: canonical correlations analysis; DUNE: Deep feature extraction by UNet-based Neuroimaging-oriented autoEncoder; FLAIR: fluid-attenuated inversion recovery; KLD: Kullback–Leibler divergence; MR: magnetic resonance; MRI: magnetic resonance imaging; OASIS: Open Access Series of Imaging Studies; ROC: receiver operating characteristic; T1Gd: T1 + gadolinium; UKB: UK Biobank; VAE: variational autoencoder.
