# Supplementary material for: DUNE: a versatile neuroimaging encoder captures brain complexity across 3 major diseases: cancer, dementia, and schizophrenia
Source: Gigascience. 2025 Oct 15;14:giaf116. doi: 10.1093/gigascience/giaf116 (PMC12527335; doi:10.1093/gigascience/giaf116)
Supplement: giaf116_Supplemental_Files [file giaf116_supplemental_files.pdf]

# SUPPLEMENTARY MATERIALS

| Name | Sources/datasets | Population | Sequences | No of cases |
|---|---|---|---|---|
| UKB | UK Biobank | General population | T1, FAIR | 19,955 |
| UPENN | University of Pennsylvania glioblastoma dataset | Glioma (GBM only) | T1Gd, FLAIR | 612 |
| UCSF | UCSF preoperative diffuse glioma MRI dataset | Glioma | T1Gd, FLAIR | 495 |
| TCGA | TCGA-LGG and TCGA GBM datasets | Glioma | T1Gd, FLAIR | 168 |
| ADNI | Alzheimer's Disease Neuroimaging Initiative 1 dataset | Alzheimer and HV | T1 | 818 |
| SchizConnect | SchizConnect database (COBRE and MCIC datasets) | Schizophrenia and HV | T1 | 336 |

**Supplemental Table S1 - Brain MR datasets used in the study.**

Images of the UKB, UPENN and UCSF datasets were used to train the models. HV: healthy volunteers; QA: quality assessment. T1Gd: T1 + gadolinium.

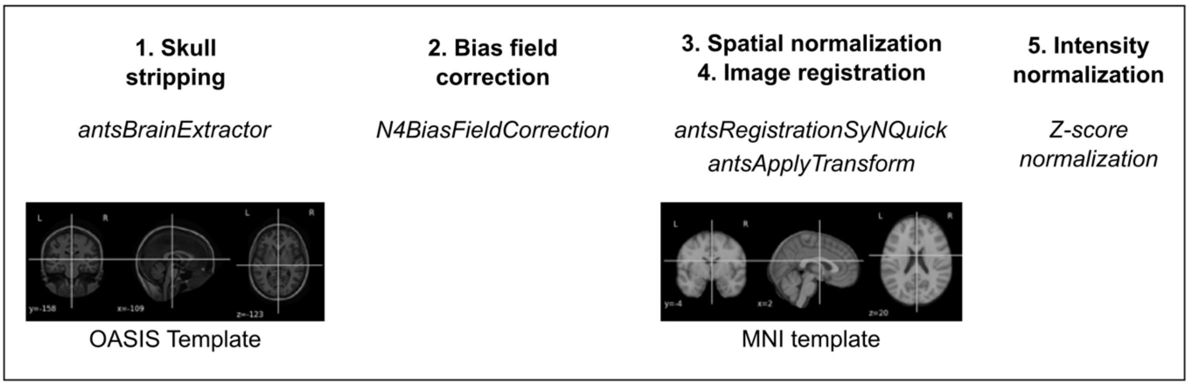


**Supplemental Figure S1 - Brain MR preprocessing steps**

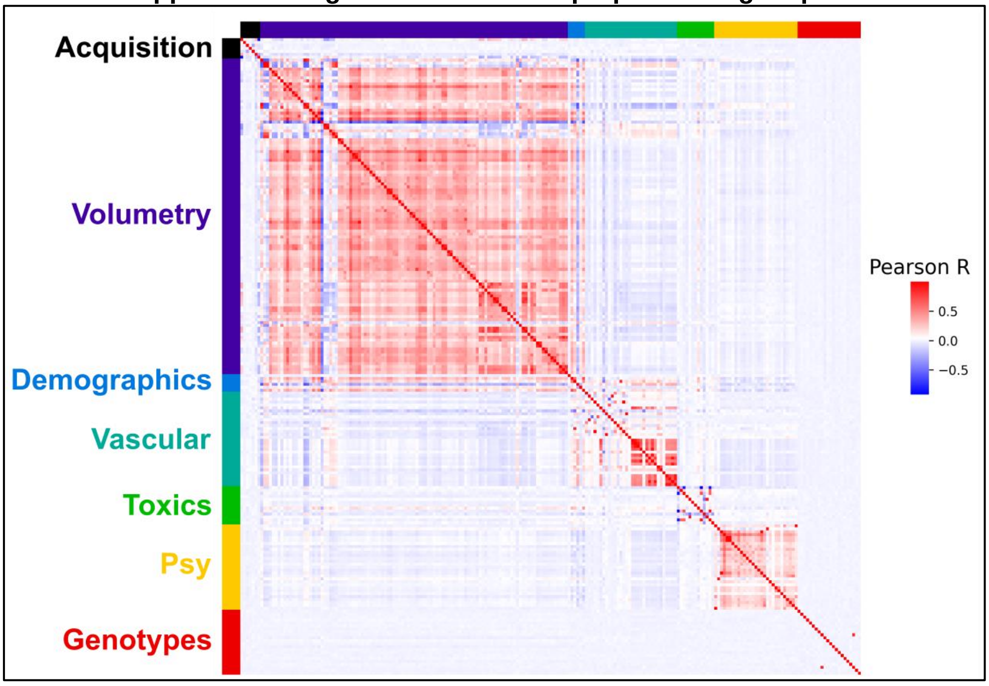


**Supplemental Figure S2 - Correlation matrix of UKB clinical variables**

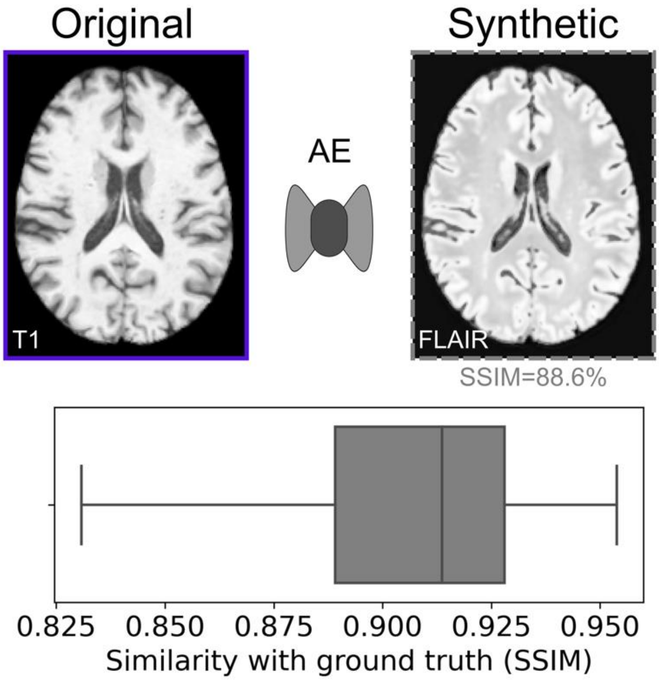


**Supplemental Figure 3 - Generation of synthetic FLAIR from T1**

# REFERENCES


1. Lenroot RK, Giedd JN. The changing impact of genes and environment on brain development during childhood and adolescence: Initial findings from a neuroimaging study of pediatric twins. Dev Psychopathol. 2008; doi: 10.1017/S0954579408000552.
2. Song R, Xu H, Dintica CS, Pan K-Y, Qi X, Buchman AS, et al.. Associations Between Cardiovascular Risk, Structural Brain Changes, and Cognitive Decline. J Am Coll Cardiol. 2020; doi: 10.1016/j.jacc.2020.03.053.
3. Gamal RM, Abozaid HSM, Zidan M, Abdelmegid MA-KF, Abdel-Razek MR, Alsayed SA-R, et al.. Study of MRI brain findings and carotid US features in systemic sclerosis patients, relationship with disease parameters. *Arthritis Research & Therapy*. 2019; doi: 10.1186/s13075-019-1877-z.
4. Kaichi Y, Kakeda S, Moriya J, Ohnari N, Saito K, Tanaka Y, et al.. Brain MR Findings in Patients with Systemic Lupus Erythematosus with and without Antiphospholipid Antibody Syndrome. *AJNR Am J Neuroradiol*. 2014; doi: 10.3174/ajnr.A3645.
5. Hemond CC, Bakshi R. Magnetic Resonance Imaging in Multiple Sclerosis. *Cold Spring Harb Perspect Med*. 2018; doi: 10.1101/cshperspect.a028969.
6. van Oostveen WM, de Lange ECM. Imaging Techniques in Alzheimer's Disease: A Review of Applications in Early Diagnosis and Longitudinal Monitoring. *International Journal of Molecular Sciences*. Multidisciplinary Digital Publishing Institute; 2021; doi: 10.3390/ijms22042110.
7. Gevaert O, Mitchell LA, Achrol AS, Xu J, Echegaray S, Steinberg GK, et al.. Glioblastoma multiforme: exploratory radiogenomic analysis by using quantitative image features. *Radiology*. 2014; doi: 10.1148/radiol.14131731.
8. Itakura H, Achrol AS, Mitchell LA, Loya JJ, Liu T, Westbroek EM, et al.. Magnetic resonance image features identify glioblastoma phenotypic subtypes with distinct molecular pathway activities. *Sci Transl Med*. 2015; doi: 10.1126/scitranslmed.aaa7582.
9. Shin I, Kim H, Ahn SS, Sohn B, Bae S, Park JE, et al.. Development and Validation of a Deep Learning–Based Model to Distinguish Glioblastoma from Solitary Brain Metastasis Using Conventional MR Images. *AJNR Am J Neuroradiol*. 2021; doi: 10.3174/ajnr.A7003.
10. Zhang J, Rao VM, Tian Y, Yang Y, Acosta N, Wan Z, et al.. Detecting schizophrenia with 3D structural brain MRI using deep learning. *Sci Rep*. Nature Publishing Group; 2023; doi: 10.1038/s41598-023-41359-z.
11. Feng X, Provenzano FA, Small SA, for the Alzheimer's Disease Neuroimaging Initiative. A deep learning MRI approach outperforms other biomarkers of prodromal Alzheimer's disease. *Alzheimer's Research & Therapy*. 2022; doi: 10.1186/s13195-022-00985-x.
