# Supplementary material for: DUNE: a versatile neuroimaging encoder captures brain complexity across 3 major diseases: cancer, dementia, and schizophrenia
Source: Gigascience. 2025 Oct 15;14:giaf116. doi: 10.1093/gigascience/giaf116 (PMC12527335; doi:10.1093/gigascience/giaf116)
Supplement: giaf116_GIGA-D-25-00076_Revision_1 [file giaf116_giga-d-25-00076_revision_1.pdf]

GigaScience

DUNE: a versatile neuroimaging encoder captures brain complexity across three major diseases: cancer, dementia and schizophrenia

--Manuscript Draft--

Manuscript Number:	GIGA-D-25-00076R1
Full Title:	DUNE: a versatile neuroimaging encoder captures brain complexity across three major diseases: cancer, dementia and schizophrenia
Article Type:	Research
Funding Information:	National Cancer Institute (R01 CA260271) — Pr Olivier Gevaert
Abstract:	Magnetic resonance images (MRI) of the brain contain complex data that pose significant challenges for computational analysis. While models proposed for brain MRIs analyses yield encouraging results, the high complexity of neuroimaging data hinders generalizability and clinical application. We introduce DUNE, a neuroimaging-oriented workflow that transforms raw brain MRI scans into standardized compact patient-level embeddings through integrated preprocessing and deep feature extraction, thereby enabling their processing by basic machine learning algorithms. A UNet-based autoencoder was trained using 3,814 selected scans of morphologically normal (healthy volunteers) or abnormal (glioma patients) brains, to generate comprehensive compact representations of the full-sized images. To evaluate their quality, these embeddings were utilized to train machine learning models to predict a wide range of clinical variables. Embeddings were extracted for cohorts used for the model development (21,102 individuals), along with 3 additional independent cohorts (Alzheimer's disease, schizophrenia and glioma cohorts, 1,322 individuals), to evaluate the model's generalization capabilities. The embeddings extracted from healthy volunteers' scans could predict a broad spectrum of clinical parameters, including volumetry metrics, cardiovascular disease (AUROC=0.80) and alcohol consumption (AUROC=0.99), and more nuanced parameters such as the Alzheimer's predisposing APOE4 allele (AUROC=0.67). Embeddings derived from the validation cohorts successfully predicted the diagnoses of Alzheimer's dementia (AUROC=0.92) and schizophrenia (AUROC=0.64). Embeddings extracted from glioma scans successfully predicted survival (C-index=0.608) and IDH molecular status (AUROC=0.92), matching the performances of previous task-oriented models. DUNE efficiently represents clinically relevant patterns from full-size brain MRI scans across several disease areas, opening ways for innovative clinical applications in neurology.
Corresponding Author:	Olivier Gevaert<br><br>UNITED STATES
Corresponding Author Secondary Information:	
Corresponding Author's Institution:	
Corresponding Author's Secondary Institution:	
First Author:	Thomas Barba
First Author Secondary Information:	
Order of Authors:	Thomas Barba<br>Bryce A. Bagley<br>Sandra Steyaert<br>Francisco Carrillo-Perez<br>Christoph Sadée<br>Michael Iv

	Olivier Gevaert
**Order of Authors Secondary Information:**	
**Response to Reviewers:**	See attached document and here:<br><br>Reviewer reports<br><br>The authors' responses are marked in blue.<br>Changes in the manuscript are marked in orange.<br><br>Reviewer #1: Reproducibility report for: DUNE: a versatile neuroimaging encoder captures brain complexity across three major diseases: cancer, dementia and schizophrenia<br>Journal: Gigascience<br>ID number/DOI: GIGA-D-25-00076<br>Reviewer(s): Laura Caquelin, Department of Clinical Neuroscience, Karolinska Institutet, Sweden [Wrote the report and reproduced the results]<br>Gustav Nilsonne, Department of Clinical Neuroscience, Karolinska Institutet, Sweden [Reviewed the final report]<br>---<br><br>1. Summary of the Study<br>The manuscript introduces DUNE , a neuroimaging-oriented (UNet-based) encoder designed to extract deep features from multisequence brain MRIs, enabling their analysis with machine learning models. It was trained on scans of healthy and glioma-affected brains to generate low-dimensional representations. These embeddings were then used to train models predicting various clinical variables and tested on independent cohorts to assess generalization.<br><br>---<br><br>2. Scope of reproducibility<br><br>According to our assessment the primary objective is: "to test prediction on MRI embeddings accurately predict clinical phenotypes of healthy individuals." (page 12)<br><br>- Outcome: the predictive performance of machine learning models trained on different MRI-derived embeddings for clinical phenotype prediction.<br>- Analysis method outcome: "The predictions from all validation folds were concatenated to generate ROC curves and compute performance metrics: weighted F1-scores for categorical variables, R^2 scores for quantitative variables, and C-indexes and Integrated Brier Scores for survival variables. The predictive performance of models using different embeddings was compared using Wilcoxon signed-rank tests with Bonferroni correction for multiple comparisons." (page 26)<br>- Main result: "Overall, the predictions were best with U-AE embeddings (0.384±0.27%), which significantly outperformed radiomics (0.144±1.58, $p<0.001$, Fig 3a). The embeddings encoded by other models (UNET, U-VAE, VAE) resulted in weaker predictions. The performances greatly varied between the different subgroups of clinical variables but showed small variation across variables within each category (Fig 3a), which tended to be highly intercorrelated (Supplemental Figure S2)." (page 12)<br><br>---<br><br>3. Availability of Materials<br>a. Data<br>- Data availability: Not open, not yet publicly available, shared on the FTP server<br>- Data completeness: Complete = all data necessary to reproduce main results are available<br>- Access Method: Private journal dropbox<br>- Repository: -<br>- Data quality: The data files have been shared and appear sufficient for running the analyses. However, no metadata is provided to describe the content, structure, or

origin of the files which limits interpretability and reusability.
b. Code
- Code availability: The code for the DUNE tools is publicly available, while the code required to reproduce the main results of the manuscript is private.
- Programming Language(s): Python
- Repository link: https://github.com/gevaertlab/BrainMR_AE_datafusion/ The code to reproduce the results is hosted on a private FTP server.
- License: Apache 2.0
- Repository status: Public for DUNE tools, Private for result reproduction code.
- Documentation: No documentation shared

----

4. Computational environment of reproduction analysis

- Operating system for reproduction: MacOS 14.7.4
- Programming Language(s): Python
- Code implementation approach: Using shared code
- Version environment for reproduction: Python 3.13.3

----

5. Results

5.1 Original study results
- Results 1: Results presented in figure 3A in the manuscript.

5.2 Steps for reproduction

-> Identify the numerical values of inferential statistical results in relation to the conclusions of Figure 3A.
- Issue 1: Numerical p-values are not provided, only indicated with asterisks in Figure 3A.
-- Resolved: The figure presents significance markers but does not report the exact p-values used for model comparisons, same for the means and standard errors across variables.
After request, the authors provided the values in files shared on private FTP servers.
- Issue 2: The raw data necessary to reproduce Figure 3A are not available.
-- Resolved: The dataset containing prediction scores (R^2 or F1-scores) across variables in each category for each model is not shared in the GitHub repository.
After request, the authors provided the raw data, and the script shared on the private FTP servers.
-> Run the Python script provided to reproduce the results of Figure 3A.
- Issue 3: Missing package installations required to run the code.
-- Resolved: There was no README file specifying the required dependencies. As a result, I had to manually identify and install the necessary packages. Some dependencies were sub-modules, so I had to search for the full package to install. For instance, sklearn.metrics is part of the larger scikit-learn package, sksurv.metrics part of scikit-survival package, and statannotations.Annotator part of statannotations package. Also statsmodels was also needed for certain statistical functions.
- Issue 4: Fixed paths that don't work on other computers.
-- Resolved: In the original code, there were fixed file paths (/Users/tom/drive/...) that only worked on one specific computer. This caused problems when running the code on other computers. To fix this, I changed the code to use relative paths, which are based on where the script is located. This way, the code can be run on any computer without needing to change the paths each time.

------------------ Start of script ------------------
```
# Configuration
# [Modification by LC]BEGIN------------
#os.chdir("/Users/tom/drive/2-postdoc/1-projects/brain_autoencoder/MS")
os.getcwd()
```

```
OUTPUT_DIR = "./GigaScience_review"
# [Modification by LC]END------------
FONTSIZE = 16
UKB_FONTSIZE = 16
UKB_FONTWEIGHT = 'regular'
SHADOW = False

# Paths
# [Modification by LC]BEGIN------------
#DATA_PATHS = {
    #"METADATA_FILE": "/Users/tom/drive/2-postdoc/1-projects/brain_autoencoder/MS/GigaScience/variable_metadata.csv",
    #"PREDICTIONS_DIR": "/Users/tom/drive/2-postdoc/1-projects/brain_autoencoder/MS/GigaScience",
#}
SCRIPT_DIR = os.path.dirname(os.path.abspath(__file__))

DATA_PATHS = {
    "METADATA_FILE": os.path.join(SCRIPT_DIR, "variable_metadata.csv"),
    "PREDICTIONS_DIR": os.path.join(SCRIPT_DIR),
}
# [Modification by LC]END------------
```
------------------- End of script -------------------

5.3 Statistical comparison Original vs Reproduced results
- Results: From the python script shared, Figure 3, the category performance_stats and the statistical_comparison_pvalues were obtained.

Comparison between the category_performance_stats and the statistical_comparison_pvalues files from the original results and the reproduced ones was conducted using R (script attached to the review).

- Comments: No particular comments.
- Errors detected: Corresponding to the sentence "Overall, the predictions were best with U-AE embeddings (0.384±0.27%), which significantly outperformed radiomics (0.144±1.58, p<0.001, Fig 3a)." (page 13):
    -- The reported value for radiomics (0.144) should be rounded to 0.145 based on the given data (0.144650928571429). No rounding error is present for the U-AE value, which is correctly reported as 0.384 based on the given data (0.384448357142857).
    -- There seems to be an inconsistency in the reported standard errors for both the U-AE and radiomics models. The paper reports a standard error of "0.384 ± 0.27%" for U-AE and "0.144 ± 1.58%" for Radiomics. Based on the provided data, the actual standard errors for these models are much higher: 1.63% (0.0162718529517984) for U-AE and 9.47% (0.0947012592300183) for radiomics.
- Statistical Consistency:
-- Graphically, the reproduced Figure 3A appears to correspond to the original one.
-- For the data in 0-category_performance_stats.csv, no differences were observed between the original results and the reproduced values.
-- For the data in 0-statistical_comparison_pvalues.csv, only one minor numerical difference was found: the p-value for category "psy", model "U-AE/RAD", differed slightly between the original (8.983406e-01) and the reproduced (8.967698e-01) results. All other values matched exactly.

We thank the reviewer for carefully identifying these numerical inconsistencies in our reported values. We have corrected these errors in the revised manuscript.
Changes made:
Results, page 9 line 40: Clinical prediction performance varied across embedding types (Fig 3a). U-AE embeddings achieved prediction scores of 0.384±1.63%, compared to radiomics (0.145±9.7%, p<0.001). UNET, U-VAE, and VAE embeddings achieved lower prediction scores across clinical variables.

Powered by Editorial Manager® and ProduXion Manager® from Aries Systems Corporation

6. Conclusion
- Summary of the computational reproducibility review
The computational reproducibility review confirmed that the results in the original study related to the main objective defined according to our assessment were reproducible using the data and code provided by the authors. The graphical representation in Figure 3A was successfully reproduced, and no differences were found between the original and reproduced values for the category_performance_stats dataset.
However, a minor numerical difference was detected in the statistical_comparison_pvalues dataset for the "psy" category, U-AE/RAD model, where the p-value slightly differed between the original (8.983406e-01) and the reproduced (8.967698e-01) results.
In addition, there are inconsistencies with rounding and discrepancies in the reported standard errors, which need to be resolved.
In summary, the results were almost fully reproducible using the original code, with only a minor discrepancy.

- Recommendations for authors
We were able to reproduce the study because we had access to the data and code provided on the private journal FTP server. To ensure that the study remains fully reproducible in the future, the following recommendations are strongly advised:

Make the code and data to reproduce all the paper openly available to the public to allow anyone to reproduce the figures and analysis of the paper.
Provide a README file or requirements.txt file to list all dependencies and ensure that the code can be run without issues. This is particularly important when using sub-modules and non-standard libraries. Clear instructions should also be included regarding how to set up the environment and run the analysis, we suggest:
oA brief description of each script or analysis pipeline.
oAn indication of which figure, table, or result in the manuscript each script corresponds to
oClear instructions on how to execute the analyses in the correct order, if applicable.
Metadata: For the datasets used or generated by the scripts, it would be helpful to include accompanying metadata files that explain:
oThe definition of each variable name.
oThe origin of each dataset (raw, processed, etc).
oAny preprocessing steps applied before analysis.
It is also recommended to document any software versions used in the analysis, as this can help avoid issues with version mismatches in future reproductions.
These improvements would enhance the reproducibility and transparency of the work.

We thank the reviewer for these recommendations to enhance the reproducibility and transparency of our work. We have addressed the suggested improvements. All code necessary to execute the complete DUNE pipeline is publicly available on our GitHub repository at https://github.com/gevaertlab/DUNE. All data (save for the UKB data, subject to data agreement) and code (jupyter notebook including code and notice) required to reproduce the figures presented in the manuscript have been deposited on Figshare and are publicly accessible at: https://figshare.com/s/d0591f66088691014bf0.

Reviewer #2: This work introduces DUNE, a versatile deep-learning model designed to extract low-dimensional embeddings from brain MRIs. The model's ability to generalize across multiple diseases, including glioma, Alzheimer's, and schizophrenia, is a significant strength, with broad potential clinical applications. However, there are areas where the manuscript can be improved:

1. While the manuscript compares DUNE to radiomics, a traditional feature extraction method, it would benefit from a comparison with more end-to-end deep learning models or architectures, such as Convolutional Neural Networks (CNNs) or Vision Transformers (ViTs). These models might perform similarly or better for the tasks at hand. This comparison would help establish the superiority or uniqueness of DUNE in terms of its capabilities and performance.
We thank the reviewer for this constructive suggestion regarding comparison with end-to-end deep learning models such as CNNs and Vision Transformers (ViTs). We acknowledge that such comparisons would provide valuable insights into the relative performance of DUNE compared to other deep learning approaches. However, several

Powered by Editorial Manager® and ProduXion Manager® from Aries Systems Corporation

practical considerations led us to focus on unsupervised feature extraction rather than end-to-end supervised models.
Vision Transformers (ViTs) present significant challenges when applied to 3D medical imaging data. ViTs typically require extensive datasets for training and lack of inductive biases compared to CNNs. For 3D brain MRI analysis, this data requirement become prohibitive, given the limited availability of medical imaging datasets compared to natural image datasets.
Regarding end-to-end supervised models, a fundamental challenge for our application lies in the heterogeneous nature of our training data. Our model development utilized three distinct datasets (UKB, UPENN, and UCSF) representing fundamentally different populations: healthy volunteers (UKB) and glioma patients (UPENN, UCSF). These cohorts have different clinical characteristics and available labels. Establishing common supervised learning targets across these disparate datasets would be challenging and could introduce significant domain-specific biases.
Despite these challenges, we conducted preliminary experiments to evaluate end-to-end CNN performance using sex prediction as a common variable across datasets. We trained a 3D resnet model on the same training dataset used for DUNE development and tested them on an external dataset (SchizConnect). These models were trained for 150 epochs but exhibited significant overfitting. The results on the external test set showed no discriminative capacity, with performance at chance level.

The unsupervised nature of DUNE offers several advantages over end-to-end supervised approaches: (i) it can leverage heterogeneous datasets without requiring common labels, (ii) it generates versatile embeddings applicable to multiple downstream tasks, (iii) it avoids overfitting to specific prediction tasks, and (iv) it provides better generalization across different clinical applications and populations.
Changes made :
Discussion, Page 15 – line 29: For 3D brain MRI analysis specifically, the computational and data requirements of transformer architectures become prohibitive given the high complexity of volumetric data and the relatively modest size of available medical imaging datasets.

2. The manuscript mentions the use of synthetic data to augment the MRI data, with noted improvements in performance. However, more details on the synthetic data generation process are needed to strengthen the manuscript. A more in-depth explanation of how the synthetic data was created and how it contributes to the model's performance would provide greater clarity.

We thank the reviewer for this suggestion. We agree that the initial description of synthetic data generation in our manuscript was insufficient. We have significantly expanded the Methods section to provide details.
Changes made :
Methods, Page 6 line 45 : To address datasets with limited sequence availability, we implemented a synthetic data enhancement strategy. A dedicated UNet-based model was trained to perform bidirectional synthesis between T1 and FLAIR sequences.
The synthetic data generation model utilized a standard 3D U-Net autoencoder architecture (with skip connections). The model consisted of 6 encoder/decoder blocks with progressive feature expansion (4, 8, 16, 32, 64, and 128 channels), processing single-channel inputs (T1 or FLAIR sequences independently). Each convolutional block comprised two sequential 3D convolution layers (kernel=3, stride=1, padding=1) followed by batch normalization, ReLU activation, and dropout (rate=0.1). Max pooling layers (2x2x2) were used for downsampling in the encoder, while transposed convolution layers (kernel=2, stride=2, padding=0) performed upsampling in the decoder.
The model was trained on the UKB dataset using 39,910 sequences (T1 and FLAIR pairs) with an 80/20 train/validation split, to establish bidirectional correspondence between T1 and FLAIR sequences, enabling the model to learn both T1→FLAIR and FLAIR→T1 synthesis. The training objective utilized SSIM loss to optimize structural similarity between synthethic and corresponding real images, with the ADAM optimizer and learning rate of $5x10^{-5}$.

3. Due to the absence of skip connections, the images reconstructed by the U-AE model inevitably suffer from a loss of details. It would be helpful to explore whether

constraints can be imposed on the loss function to encourage the network to preserve image details. For instance, incorporating distance function constraints (as in Sun et al., 2020, 10.1002/hbm.26098) could be an approach to ensure better retention of details for segmented tissue map.
We thank the reviewer for this insightful suggestion regarding the preservation of image details in our U-AE model architecture. Following the reviewer's recommendation to explore distance function constraints as described in Sun et al. (2020), we conducted additional experiments to investigate this trade-off.
We implemented an improved loss function that combines SSIM reconstruction loss with gradient-based constraints to preserve fine-grained details:
$L\_improved = \alpha \times L\_SSIM + \beta \times L\_gradient$
where L_gradient penalizes differences in spatial gradients across all three dimensions, encouraging the network to maintain local image details despite the absence of skip connections.

We trained a modified U-AE model ("constraintUAE", $\alpha=1$, $\beta=0.1$) using this enhanced loss function and evaluated its performance on clinical prediction on three independent cohorts (TCGA, SCHIZO, and ADNI datasets).
• The constraintUAE achieved significantly improved SSIM scores (~80%) compared to the standard U-AE :

However, when evaluating the extracted embeddings for clinical phenotype prediction, the constraintUAE consistently underperformed compared to the standard U-AE across all three external validation datasets:

This experiment further illustrates the trade-off between reconstruction quality and embedding informativeness.

Changes made :
Discussion, Page 14 line 25: Surprisingly, this architecture demonstrated the poorest reconstruction capabilities while producing the most clinically relevant embeddings, highlighting a counter-intuitive finding that reconstruction performance does not predict embedding quality for clinical applications. We further validated this principle through additional experiments with enhanced loss functions incorporating gradient-based detail preservation constraints, which confirmed improved reconstruction quality at the cost of reduced clinical prediction performance (data not shown). This trade-off represents an important consideration in autoencoder architectures, where the removal of skip connections forces all information through the bottleneck layer, creating more informative representations despite reduced pixel-level accuracy.
Reviewer #3: The article addresses the problem of feature extraction from MRI brain images, aiming to support the diagnosis of various brain diseases and pathological conditions. Given the nature of the task, supervised classification networks (such as AlexNet, ResNet, etc.) would typically be considered the primary approach, as these architectures are explicitly designed to extract features enabling optimal discrimination between classes - different pathological brain states.
The authors, however, hypothesize that encoder-decoder neural network architectures (e.g., U-Net and its variants) can effectively extract embedding features relevant to diagnosis. They further propose using unsupervised learning methods, based on the assumption that the reconstructed image should closely resemble the input, to train these networks. The feature extraction capabilities of four encoder-decoder-based models are compared, followed by an analysis of the correlation between the extracted features and diagnostic categories or known medical/radiological markers.
Demonstrating that unsupervised encoder-decoder networks can extract diagnostically meaningful features appears to be the central contribution of this study. The authors also claim to have introduced a novel methodology for analyzing MRI images, which they name DUNE.
The input data for the study were sourced from six datasets: UK, TCGA, UPENN, ADNI, UCSF, and SchixConnect. However, the descriptions of how the training data were prepared are scattered across various parts of the paper and lack coherence. They appear in the abstract, introduction, Figure 1a, results section, and the Materials and methods section. Multiple, sometimes contradictory numbers of training examples are mentioned: 21,102 individuals, 3,814 MRI scans, 1,322 individuals, 500,000

Powered by Editorial Manager® and ProduXion Manager® from Aries Systems Corporation

healthy individuals, etc. In the Results section, it is mentioned that 800 cases were selected from 19,955, yet another statement refers to n=1,600 scans, and Figure 3 caption mentions the use of all 19,955 cases.
The manuscript states: "To prevent overfitting the model to normal healthy brains, only 800 randomly selected cases were finally kept for the model development (n=1,600 T1 and FLAIR scans)." This justification is questionable, as reducing training set size is generally counterproductive in preventing overfitting; rather, increasing data volume is a solution to this problem. It seems the reduction was motivated more by a desire to balance the dataset than by overfitting concerns.

We thank the reviewer for this observation regarding the clarity and consistency of our dataset descriptions and training methodology. We have clarified this information in the revised manuscript to improve comprehensibility.
To clarify the numerical discrepancies highlighted by the reviewer:
Datasets for autoencoder training (development):
UKB: 800 cases selected from 19,955 available (1,600 scans: T1 + FLAIR)
UPENN: 612 cases (1,224 scans: T1Gd + FLAIR)
UCSF: 495 cases (990 scans: T1Gd + FLAIR)
Total training dataset: 1,907 cases, 3,814 scans (80% training, 20% validation)
Datasets for clinical evaluation of the embeddings:
Internal evaluation (cohorts used for model development)
oUKB (full cohort): 19,955 cases (39,910 scans: T1 + FLAIR) - used for clinical prediction tasks
oUPENN: 612 cases (1,224 scans: T1Gd + FLAIR)
oUCSF: 495 cases (990 scans: T1Gd + FLAIR)
External evaluation (unseen cohorts)
oTCGA: 168 cases (336 scans: T1Gd + FLAIR) - external glioma validation
oADNI: 818 cases (818 scans: T1 only) - Alzheimer's disease validation
oSchizConnect: 336 cases (336 scans: T1 only) - schizophrenia validation
Total evaluation datasets: 21,277 cases, 41,400 scans
Summary of key numbers mentioned in manuscript:
21,102 individuals: Refers to UKB evaluation cohort (19,955) + other datasets (1,147) used for embedding evaluation
3,814 MRI scans: Specific number of scan sequences used for autoencoder training
1,322 individuals: Participants in the three independent validation cohorts (TCGA + ADNI + SchizConnect)
The reviewer correctly points out that our initial explanation regarding "overfitting to normal healthy brains" was imprecise. We appreciate this feedback and provide a clearer rationale for this methodological choice.
The reduction from 19,955 to 800 UKB cases was primarily motivated by the need to maintain balanced representation between healthy and pathological brain morphologies in our training dataset. Our initial experiments using the complete UKB cohort (≈20,000 cases) alongside the glioma datasets (≈1,100 cases) resulted in a severe class imbalance that biased the model toward normal brain morphology. This imbalance led to poor-quality embeddings for tumor cases while maintaining good performance on healthy brains. By downsampling the UKB dataset to match the size of the glioma datasets, we achieved improved quality of tumor embeddings while preserving the model's performance on normal brain images. This strategic balancing was crucial for developing a versatile feature extractor capable of handling both healthy and pathological brain morphologies effectively.
Additionally, we have validation results demonstrating that DUNE's performance generalizes well beyond the training cohort, suggesting that our training dataset size was sufficient for learning robust feature representations.
Changes made:
Methods, Page 4 line 8: new Datasets section : Several datasets served for development and validation of DUNE (Fig 1a, Supplemental Table S1). The model development was made using brain MRI scans from the UKB, UPENN and UCSF datasets. The UKB (UK Biobank) dataset provides extensive clinical and radiological data for a large cohort of approximately 500,000 healthy individuals. Brain MRI (T1 and FLAIR sequences), and clinical data of 19,955 individuals (39,910 scans) were downloaded and preprocessed as described below. In addition to these healthy brains were included scans (T1+gadolinium [T1Gd] and FLAIR) from the glioma cohorts UPENN (UPenn-GBM, 612 cases, 1,224 scans) and UCSF (UCSF-PDGM, 495 cases, 990 scans), to make the model equally capable of extracting features from

morphologically abnormal brains.
For model evaluation and generalization assessment, three independent validation cohorts were utilized, representing distinct neurological conditions:
1.Glioma validation cohorts: The previously unseen TCGA-GBM and TCGA-LGG datasets (168 cases combined, 336 scans T1Gd and FLAIR) provided external validation for brain tumor applications.
2.Alzheimer's disease cohort: Data from the ADNI database (818 cases; T1 scans only) enabled evaluation of neurodegenerative disease detection capabilities.
3.Schizophrenia cohort: The SchizConnect database, comprising COBRE and MCIC datasets (336 total cases; T1 scans only), allowed assessment of psychiatric disorder classification performance.
Methods, Page 5 line 30 : new Autoencoder training algorithm section : To prevent overfitting the model to normal healthy brains, only 800 randomly selected cases were finally kept from the UKB cohort for the model development, corresponding to 1,600 scans (T1 and FLAIR sequences). This strategic reduction from the full UKB cohort (19,955 individuals) was crucial to maintain balanced representation between healthy and pathological brain morphologies. Combined with glioma scans from UPENN (1,224 scans) and UCSF (990 scans), the final training dataset comprised 3,814 scans (T1,T1Gd, FLAIR; 80% training, 20% validation).

Further confusion arises from inconsistent statements about validation method. The manuscript mentions an 80/20 train-validation split, suggesting a hold-out validation approach. Elsewhere, a "5-fold cross-validation procedure with oversampling" is metioned, indicating a different evaluation strategy. What is more, the use of oversampling contradicts the earlier decision to reduce the dataset size (from 19,955 to 800), raising questions about the methodological consistency. It is also problematic that the parameter n is used with different meanings and values throughout the manuscript, which is inappropriate from a formal scientific perspective. Overall, the description of data preparation for training procedures is imprecise, inconsistent, and confusing. Scattering this critical information across multiple sections hinders comprehension. The authors should consolidate all information regarding training data preparation into a single, clearly structured section. They must ensure consistency and precision in terminology and methodological description, particularly with regard to data volumes and evaluation protocols.

We thank the reviewer for highlighting this particular point. We acknowledge that the scattered presentation of evaluation strategies across different sections created confusion and apparent contradictions. We have now consolidated and clarified our evaluation methodology in the revised manuscript:
Two distinct validation phases with different strategies:
Autoencoder training validation: We used an 80/20 train-validation split on the development dataset (3,814 scans from UKB, UPENN, and UCSF). This hold-out validation approach was used exclusively for monitoring autoencoder training convergence and preventing overfitting during the unsupervised feature learning phase.
Clinical prediction evaluation: We used 5-fold cross-validation with oversampling for evaluating the quality of extracted embeddings in downstream clinical prediction tasks. This cross-validation was applied separately to each clinical evaluation dataset (UKB for phenotype prediction, and independent cohorts for disease-specific validation).
The reviewer correctly identified an apparent contradiction between dataset reduction (19,955 → 800 UKB cases) and subsequent oversampling. We clarify that these operations serve different purposes and occur at different stages:
Dataset reduction (19,955 → 800): Applied during autoencoder training to balance healthy vs. pathological brain morphologies in the development phase
Oversampling: Applied during clinical prediction evaluation to address class imbalance in specific prediction tasks (e.g., rare genetic variants, disease vs. control classification)
These strategies are complementary rather than contradictory: the initial reduction ensures balanced representation for feature learning, while oversampling addresses prediction-specific class imbalances during evaluation.
We also acknowledge the inappropriate use of parameter "n" with different meanings throughout the manuscript. We have standardized our terminology in the revised version:
"Participants" or "cases" for individual subjects

"Scans" or "sequences" for individual MRI acquisitions
"Samples" only when referring to individual data points in machine learning contexts
Consistent numerical references with clear context for each usage
Changes made:
Methods, Page 5 line 30 : new Autoencoder training algorithm section
Methods, Page 6 line 7 : new Patient-level embedding generation section
Methods, Page 6 line 19 : new Statistical analysis and embedding evaluation section

The manuscript deviates from the standard IMRAD (Introduction, Methods, Results, and Discussion) structure. Specifically, the Materials and Methods section is positioned after the description of the conducted experiments and the Results section. This significantly hampers readability, as readers are presented with experimental outcomes before being given a clear understanding of the methods used to obtain them. Fragmentary details about the preparation of training and validation datasets appear in the premature Results section. These methodological descriptions should be consolidated into a coherent and precise Materials and Methods section placed before the Results section. Moreover, the Results section includes interpretative statements and conclusions - such as "the UNET model outperformed the other autoencoders" and "Variational autoencoders showed similar results" - that precede the presentation of the corresponding quantitative experimental results. Such conclusions should be reserved for the Discussion section and must be explicitly supported by quantitative results presented beforehand in the Results. The current structure and order of the content make the article difficult to follow and give it a disorganized feel.
We thank the reviewer for this important feedback regarding manuscript structure and organization. We have completely reorganized the manuscript to follow the standard IMRAD structure. The Materials and Methods section has been moved to precede the Results section, ensuring that readers have a clear understanding of our methodology before encountering experimental outcomes.
We have consolidated all methodological descriptions that were previously scattered across multiple sections into a comprehensive Materials and Methods section. This includes:
Complete dataset descriptions with clear numerical references
Detailed preprocessing pipeline
Autoencoder architecture specifications
Training algorithms and hyperparameters
Statistical analysis and evaluation frameworks
Validation strategies for both autoencoder training and clinical prediction
The reviewer correctly identified that our Results section contained interpretative statements and conclusions that should be reserved for the Discussion. We have revised the Results section to present quantitative findings objectively, moving all interpretative statements and comparative conclusions to the Discussion section where they are properly supported by the previously presented quantitative results.

Additionally, some of the terminology used throughout the manuscript is vague, inconsistently applied, or insufficiently defined. The authors frequently use the term embeddings in reference to features extracted by neural networks. While a partial explanation is offered in the Feature Extraction subsection, the manuscript alternates between the terms features and embeddings, which may confuse readers. Furthermore, the terms radiomic, genetic and diagnostic embeddings/features are introduced without adequate explanation.
We thank the reviewer for highlighting these important terminological inconsistencies. We acknowledge that our inconsistent use of terminology, particularly the alternation between "features" and "embeddings," created confusion and reduced manuscript clarity. We have systematically revised the manuscript to establish clear and consistent definitions for all technical terms. The following standardized terminology is now used throughout:
"Features": Low-level numerical characteristics extracted from individual MRI sequences at the autoencoder bottleneck layer (e.g., 1,000-5,000 values per sequence)
"Embeddings": High-level patient-specific representations created by concatenating features from multiple MRI sequences to generate comprehensive patient signatures (e.g., combined T1 + FLAIR features)
"Radiomics": Traditional quantitative imaging features extracted using established mathematical algorithms (texture, shape, intensity distributions)

Powered by Editorial Manager® and ProduXion Manager® from Aries Systems Corporation

The authors also use the expressions high-dimensional and low-dimensional embeddings, which are not precisely defined and appear to be used in a loosely conceptual sense. It seems they are referring to types of data commonly described in machine learning as unstructured, poorly-structured, or well-structured. This imprecise terminology introduces ambiguity into the manuscript. For instance, the very first sentence of the abstract states: "Magnetic resonance images (MRI) of the brain exhibit high dimensionality that pose significant challenges for computational analysis." This phrase is problematic: MRI data can be 2D, 3D, or 4D (e.g., including temporal dynamics). In this meaning of the term dimensionality, the statement is misleading and may be misunderstood. As currently written, it confuses dimensionality in the mathematical sense with data structure complexity.
We thank the reviewer for this observation regarding our imprecise use of dimensionality terminology. We agree that our terminology inappropriately conflated mathematical dimensionality with data complexity, creating confusion and ambiguity. The reviewer correctly identified the misleading opening sentence of our abstract as a prime example of this confusion. We have revised this and similar statements throughout the manuscript to distinguish clearly between spatial dimensionality and data complexity.
Changes made:
Abstract, page 2 line 9: "Magnetic resonance images (MRI) of the brain contain complex data that pose significant challenges for computational analysis."
Introduction, page 3 line 16: "MRI datasets are characterized by their computational complexity, stemming from the large number of voxels per image (≥5 million voxels per sequence) and the multisequence nature of clinical protocols (T1, FLAIR, contrast injection sequences, etc.). This high-dimensional space, combined with the subtle morphological patterns that encode clinical information, necessitates sophisticated computational approaches for effective analysis."
Throughout the manuscript: We have standardized our terminology to use:
"High-dimensional data" when referring to the mathematical concept of many variables/features (e.g., millions of voxels)
"Complex data" or "computationally complex" when referring to the challenging nature of processing and analyzing neuroimaging data
"Low-dimensional embeddings" when referring to the reduced feature space (1,000-5,000 features) generated by the autoencoders

The authors state that they "introduce DUNE, a neuroimaging-oriented encoder designed to extract deep features from multisequence brain MRIs." However, the manuscript primarily presents comparisons between existing and modified versions of well-known encoder-decoder neural network architectures, such as U-Net. The preprocessing methods applied to the MRI data are described only superficially and without sufficient justification for their use. Therefore, it remains unclear what exactly DUNE is, how it differs from the established architectures presented, and what is its novel contribution.

We thank the reviewer for this important critique regarding the clarity of DUNE's contribution and novelty.
We acknowledge that our presentation created confusion about what DUNE actually represents. DUNE is not a single novel neural network architecture, but rather a comprehensive workflow that integrates three core components to transform raw brain MRI scans into standardized, clinically-relevant patient-level embeddings: (1) a standardized preprocessing pipeline using ANTs that ensures consistent image quality and spatial alignment across different acquisition protocols and scanner types, (2) feature extraction using the U-AE architecture (UNet without skip connections) identified through systematic benchmarking as optimal for clinical embedding generation, and (3) multi-sequence feature aggregation through sequential processing and concatenation, providing operational flexibility for incomplete imaging datasets. DUNE's primary novel contributions are: the counter-intuitive finding that reconstruction quality does not predict embedding quality for clinical applications (U-AE demonstrated poorest reconstruction but best clinical performance), the demonstration of robust performance across multiple disease areas and institutions, and the discovery that synthetic sequence generation can enhance embedding quality when sequences are missing. Through comprehensive validation across healthy volunteers, glioma patients, Alzheimer's disease, and schizophrenia cohorts, we demonstrate that this integrated

Powered by Editorial Manager® and ProduXion Manager® from Aries Systems Corporation

workflow generates embeddings with superior clinical relevance compared to traditional radiomics approaches and individual autoencoder architectures evaluated in isolation.
Changes made:
Abstract, page 2 line 22: "We introduce DUNE, a neuroimaging-oriented workflow that transforms raw brain MRI scans into standardized compact patient-level embeddings through integrated preprocessing and deep feature extraction"
Introduction, page 3 line 35: “In this study, we introduce DUNE (Deep feature extraction by UNet-based Neuroimaging-oriented autoEncoder), a comprehensive neuroimaging workflow that transforms multisequence raw brain MRI scans into standardized compact patient-level embeddings. The workflow integrates standardized preprocessing, unsupervised feature extraction using a UNet-based autoencoder, and multi-sequence feature aggregation to generate comprehensive patient-level embeddings”.
Results section, page 4 line 3:
new "Study overview" subsection: “DUNE represents a workflow that transforms multisequence brain MRI data into standardized compact patient-level embeddings through integrated preprocessing, feature extraction and agregation, addressing MRI acquisition variability across institutions. An overview of the workflow for development and evaluation is presented in Fig 1a.”
Methods section, page 6 line 7: new "Patient-level embedding generation" subsection

The analysis may be confounded by the way pathological categories are mapped to different source datasets. Since certain brain disorders are primarily represented within specific databases, the observed embeddings may inadvertently capture dataset-specific characteristics - such as imaging protocols, scanner types, or demographic biases - rather than pathologically meaningful features. The manuscript does not address this potential source of bias.
We thank the reviewer for raising this important concern about potential confounding between pathological categories and dataset-specific characteristics. This is indeed a critical consideration in multi-institutional neuroimaging studies.
We acknowledge that certain brain disorders are primarily represented within specific databases in our study design. However, our methodological approach tends to mitigate this potential source of bias. First, the preprocessing pipeline using ANTs was specifically designed to harmonize images across different acquisition protocols and scanner types, effectively reducing technical variability that could confound pathological signals.
Second, multiple lines of evidence suggest that DUNE embeddings capture biological rather than technical characteristics. Our embeddings successfully predict fundamental biological features (sex, IDH1 or APOE4 status) across different datasets, suggesting that the model captures fundamental biological patterns rather than dataset-specific artifacts. Moreover, DUNE demonstrates robust performance on external validation cohorts acquired using different protocols than training datasets (TCGA, ADNI, SchizConnect). If embeddings were primarily capturing scanner or protocol differences, we would expect degraded performance on these cross-institutional predictions, which we do not observe.
Furthermore, the foundation model paradigm in machine learning increasingly relies on training with heterogeneous, multi-institutional data without extensive harmonization to remove technical variations. This approach enhances model robustness by learning representations that generalize across diverse acquisition conditions. Our validation demonstrates that DUNE embeddings maintain clinical relevance across independent cohorts with different imaging protocols, supporting their biological rather than technical basis.
Changes made:
Discussion, Page 16 line 42: Another potential limitation is the risk for dataset-specific confounding, as certain diseases are primarily represented within specific institutional cohorts, potentially leading to embeddings capturing technical rather than pathological characteristics. However, we implemented strategies to mitigate this risk, and multiple lines of evidence suggest that DUNE embeddings capture biological rather than technical features. First, we employed the ANTs-based preprocessing pipeline, which harmonizes images across different acquisition protocols and scanner types, thus reducing technical variability. Additionally, DUNE successfully predicts fundamental biological features (for example sex, IDH1 or APOE4 status) across different datasets, and demonstrates robust performance on external validation cohorts acquired using

	different protocols than training datasets (TCGA, ADNI, SchizConnect). If embeddings primarily encoded scanner-specific artifacts, we would expect degraded performance on these cross-institutional predictions, which we do not observe.<br><br>While the authors claim to introduce a novel tool/methodology - DUNE - for extracting deep features from multisequence brain MRIs, the manuscript does not clearly distinguish this work from previously published studies, including their own earlier preprint available on medRxiv (https://www.medrxiv.org/content/10.1101/2025.02.24.25322787v1).<br><br>We thank the reviewer for bringing attention to the medRxiv preprint. We clarify that the medRxiv preprint is the same work as the current manuscript submitted to GigaScience, not a separate previous publication.<br>The medRxiv preprint represents an earlier version of this manuscript that was posted to enable early sharing of our findings with the scientific community during the peer review process.<br><br>At present, the article is not suitable for publication. The authors need to revise the manuscript thoroughly: restructuring it according to standard scientific conventions, improving logical flow and clarity, and using precise language when describing the data, their properties, and the applied methods. Furthermore, the claimed contributions - particularly the nature and novelty of DUNE - must be clearly defined and substantiated.<br>Despite the concerns outlined above, the manuscript addresses an important and timely problem in the field of medical diagnosis and machine learning. The idea of leveraging unsupervised encoder-decoder networks to extract diagnostically relevant features from multisequence MRI is promising, and with appropriate clarification, restructuring, and methodological precision, this work has the potential to make a meaningful contribution. I encourage the authors to revise the manuscript thoroughly, with particular attention to methodological transparency, structural organization, and clearer articulation of the novel aspects. I look forward to seeing a more refined version of this study in a future submission.
**Additional Information:**	
**Question**	**Response**
Are you submitting this manuscript to a special series or article collection?	No
**Experimental design and statistics**<br><br>Full details of the experimental design and statistical methods used should be given in the Methods section, as detailed in our Minimum Standards Reporting Checklist. Information essential to interpreting the data presented should be made available in the figure legends.<br><br>Have you included all the information requested in your manuscript?	Yes
**Resources**	Yes

A description of all resources used, including antibodies, cell lines, animals and software tools, with enough information to allow them to be uniquely identified, should be included in the Methods section. Authors are strongly encouraged to cite Research Resource Identifiers (RRIDs) for antibodies, model organisms and tools, where possible.<br><br>Have you included the information requested as detailed in our Minimum Standards Reporting Checklist?	
**Availability of data and materials**<br><br>All datasets and code on which the conclusions of the paper rely must be either included in your submission or deposited in publicly available repositories (where available and ethically appropriate), referencing such data using a unique identifier in the references and in the "Availability of Data and Materials" section of your manuscript.<br><br>Have you have met the above requirement as detailed in our Minimum Standards Reporting Checklist?	Yes
GigaScience has policies and guidelines in place for the use of generative AI-writing tools such as ChatGPT. If you have used such writing tools to assist with writing the manuscript this must be declared and cited in the text. Authors should not list AI-writing tools and other AI-assisted technologies as an author or co-author and should acknowledge that they are fully responsible for text generated or refined by AI-writing tools.<p><br><br>A summary of use (particularly in the introduction or among methods) needs to be included at the end of the paper, and the outputs should also be included as a supplementary file hosted in GigaDB or other open repositories. Please read our guidelines for more information. <p>	No

By submitting to GigaScience, you are aware of the journal's AI-writing tools policy, and if you have declared use of such tools below, you have acknowledged this where appropriate in your manuscript and have made a summary of use and outputs available. <p> <b>AI-assisted writing tools have been used in the preparation of this manuscript?	

Manuscript

Click here to access/download;Manuscript;2024-03-19-BrainAE_V13clean_final.docx

DUNE: a versatile neuroimaging encoder captures brain complexity across three major diseases: cancer, dementia and schizophrenia

Thomas Barba[1,2], Bryce A. Bagley[1], Sandra Steyaert[1], Francisco Carrillo-Perez[1], Christoph Sadée[1], Michael Iv[3], Olivier Gevaert[1,4*]

Affiliations:

1. Stanford Center for Biomedical Informatics Research (BMIR), Department of Medicine, Stanford University; Stanford, CA 94305, USA
2. Department of Internal Medicine, Edouard Herriot Hospital; Lyon, France
3. Department of Radiology, Stanford University; Stanford, CA 94305, USA
4. Department of Biomedical Data Science, Stanford University; Stanford, CA 94305, USA

***Corresponding authors:**

1) Prof Olivier Gevaert, PhD
Associate Professor of Medicine and Biomedical Data Science
Stanford University
(650) 721-2378
ogevaert@stanford.edu

2) Dr Thomas Barba, MD, PhD
Assistant Professor of Internal Medicine
Department of Internal Medicine
Lyon University Hospital, France
thomas.barba@chu-lyon.fr

Thomas Barba [0000-0003-1281-0433]; Bryce A Bagley [0000-0001-9702-1681]; Sandra Steyaert [0000-0002-0996-1364]; Francisco Carrillo-Perez [0000-0003-0974-4092]; Christoph Sadée [0000-0001-7416-1470]; Michael Iv [0000-0001-6794-7213]; Olivier Gevaert [0000-0002-9965-5466];

ABSTRACT

Magnetic resonance images (MRI) of the brain contain complex data that pose significant challenges for computational analysis. While models proposed for brain MRIs analyses yield encouraging results, the high complexity of neuroimaging data hinders generalizability and clinical application. We introduce *DUNE*, a neuroimaging-oriented workflow that transforms raw brain MRI scans into standardized compact patient-level embeddings through integrated preprocessing and deep feature extraction, thereby enabling their processing by basic machine learning algorithms. A UNet-based autoencoder was trained using 3,814 selected scans of morphologically normal (healthy volunteers) or abnormal (glioma patients) brains, to generate comprehensive compact representations of the full-sized images. To evaluate their quality, these embeddings were utilized to train machine learning models to predict a wide range of clinical variables. Embeddings were extracted for cohorts used for the model development (21,102 individuals), along with 3 additional independent cohorts (Alzheimer's disease, schizophrenia and glioma cohorts, 1,322 individuals), to evaluate the model's generalization capabilities. The embeddings extracted from healthy volunteers' scans could predict a broad spectrum of clinical parameters, including volumetry metrics, cardiovascular disease (AUROC=0.80) and alcohol consumption (AUROC=0.99), and more nuanced parameters such as the Alzheimer's predisposing APOE4 allele (AUROC=0.67). Embeddings derived from the validation cohorts successfully predicted the diagnoses of Alzheimer's dementia (AUROC=0.92) and schizophrenia (AUROC=0.64). Embeddings extracted from glioma scans successfully predicted survival (C-index=0.608) and IDH molecular status (AUROC=0.92), matching the performances of previous task-oriented models. DUNE efficiently represents clinically relevant patterns from full-size brain MRI scans across several disease areas, opening ways for innovative clinical applications in neurology.

One Sentence Summary

We propose a brain MRI-specialized encoder, which extracts versatile and compact embeddings from full-size scans.

INTRODUCTION

The morphology of the human brain is determined by genetic and embryological factors specific to each individual, and is constantly remodeled throughout life in response to internal and environmental factors[1]. A broad range of pathological processes shape brain morphology, either affecting the brain primarily (e.g., cancer and inflammatory and neurodegenerative diseases) or secondarily in systemic disorders (e.g., cardiovascular and systemic autoimmune diseases)[2–4]. Therefore, the morphology of the brain recapitulates numerous formative events that occur during an individual's lifetime, providing a snapshot of their health at a particular time. Advances in brain imaging over the last decades have enabled doctors to better characterize and treat a wide range of conditions. In this setting, magnetic resonance imaging (MRI) have established it as a fundamental tool in the diagnosis and surveillance of cancers, neurodegenerative disorders, and inflammatory diseases of the central nervous system[5,6]. With the ongoing development of new imaging sequences, the quality of brain imaging continues to improve, achieving unprecedented precision in depicting brain morphology. However, while these increasingly complex representations of the brain are of undeniable interest in the management of brain diseases, their full potential has yet to be realized.

MRI datasets are characterized by their computational complexity, stemming from the large number of voxels per image (≥5 million voxels per sequence) and the multisequence nature of clinical protocols (T1, FLAIR, contrast injection sequences, etc.). This high-dimensional space, combined with the subtle morphological patterns that encode clinical information, necessitates sophisticated computational approaches for effective analysis.

In the era of artificial intelligence, authors have proposed deep-learning models capable of leveraging these complex imaging data to predict clinical parameters such as detection of brain tumors[7,8], and diagnosis of neuropsychiatric and neurodegenerative diseases [9–11]. However, these models have been limited in their clinical translation due to several reasons. First, their training requires large datasets and extensive computational resources because of the considerable number of internal parameters that need to be trained. Second, their monothematic nature implicates that they learn to perform only one task at a time (e.g. diagnosis prediction or tumor segmentation). Third, these models are usually trained on a few specific datasets, which affects their performance on externally acquired images, a problem known as overfitting and lack of generalization[12].

General-purpose feature extractors could offer promising solutions to tackle the complexities of processing high-dimensional data. Such models can generate versatile representations that capture essential information from the input data, while reducing computational demands for downstream applications [13,14].

In this study, we introduce ***DUNE*** (**D**eep feature extraction by **U**Net-based **N**euroimaging-oriented auto**E**ncoder), a comprehensive neuroimaging workflow that transforms multisequence raw brain MRI scans into standardized compact patient-level embeddings. The workflow integrates standardized preprocessing, unsupervised feature extraction using a UNet-based autoencoder, and multi-sequence feature aggregation to generate comprehensive patient-level embeddings [15–18]. To ensure its robustness, the model was trained on MRI scans of morphologically normal and abnormal brains issued from three datasets. The quality of the extracted embeddings was evaluated by employing them to infer numerous clinical parameters using simple machine learning models. Different autoencoder architectures were compared in terms of reconstruction capabilities and quality of embeddings. Based on comprehensive evaluations across multiple clinical applications, we identified a UNet architecture without skip connections (U-AE) as the optimal model for *DUNE*. Surprisingly, this architecture, while being the least effective in reconstructing MRI data, produced the most clinically relevant embeddings, underscoring the importance of using appropriate evaluation metrics commensurate with their intended use. To demonstrate its generalizability, *DUNE* was used to extract embeddings of scans of individuals from three independent cohorts representing three disease areas. These embeddings were used to successfully predict a broad spectrum of clinical parameters, including cognitive dysfunction, psychiatric disorders, genetic traits (APOE4 status), glioma molecular features (IDH status) and patient survival, with results matching or outperforming previously published models directly processing the raw imaging data. Finally, we demonstrate that augmenting the MRI data with synthetic images can enhance the quality of the produced embeddings, resulting in improved performances in downstream clinical inference tasks. In summary, *DUNE* provides efficient general-purpose embeddings of multi-modal brain MRI data independent of the downstream use case.

DATA AND METHODS

Study overview

DUNE represents a workflow that transforms multisequence brain MRI data into standardized compact patient-level embeddings through integrated preprocessing, feature extraction and aggregation, addressing MRI acquisition variability across institutions. An overview of the workflow for development and evaluation is presented in Fig 1a.

Datasets

Several datasets served for development and validation of *DUNE* (Fig 1a, Supplemental Table S1). The model development was made using brain MRI scans from the UKB, UPENN and UCSF datasets. The UKB (UK Biobank) dataset provides extensive clinical and radiological data for a large cohort of approximately 500,000 healthy individuals. Brain MRI (T1 and FLAIR sequences), and clinical data of 19,955 individuals (39,910 scans) were downloaded and preprocessed as described below. In addition to these healthy brains were included scans (T1+gadolinium [T1Gd] and FLAIR) from the glioma cohorts UPENN (UPenn-GBM, 612 cases, 1,224 scans) and UCSF (UCSF-PDGM, 495 cases, 990 scans), to make the model equally capable of extracting features from morphologically abnormal brains.

For model evaluation and generalization assessment, three independent validation cohorts were utilized, representing distinct neurological conditions:

1. **Glioma validation cohorts**: The previously unseen TCGA-GBM and TCGA-LGG datasets (168 cases combined, 336 scans T1Gd and FLAIR) provided external validation for brain tumor applications.
2. **Alzheimer's disease cohort**: Data from the ADNI database (818 cases; T1 scans only) enabled evaluation of neurodegenerative disease detection capabilities.
3. **Schizophrenia cohort**: The SchizConnect database, comprising COBRE and MCIC datasets (336 total cases; T1 scans only), allowed assessment of psychiatric disorder classification performance.

Preprocessing

All the images were standardized through the preprocessing module of *DUNE* depicted in Supplemental Figure S1, based on the Advanced Normalization Tools (ANTs) [19,20]. When needed, DICOM files were converted to NIFTI files. Images of each sequence (T1, T1Gd, FLAIR) then underwent a series of transformations. Skull stripping was performed with the ANTS Brain extractor tool using templates and probability masks from the OASIS (Open Access Series of Imaging Studies) datasets *[21]*. The N4 bias field correction algorithm was applied and FLAIR images were co-registered to T1. All images were then warped into a common space using the MNI-152 T1 1mm template ("Montreal Neurologic Institute [MNI] space") *[22]*. Pixel intensities were finally Z-score normalized. The final dimensions (X, Y, Z) of the pre-processed images were [182, 218, 160] pixels (1px/mm, 1 grayscale channel). This standardized preprocessing pipeline ensured consistent image quality and spatial alignment across all datasets, enabling effective multi-institutional model training while maintaining compatibility with different acquisition protocols and scanner types.

For the UKB dataset specifically, clinical variables from the whole dataset were collected, excluding those with a missing rate ≥15%. The data were standardized and data imputation was performed using the k-Nearest Neighbors algorithm (k=140).

Feature extractor architecture

Four candidate architectures were systematically benchmarked to identify the optimal feature extractor. Guided by previous research *[23]*, we focused our investigation on unsupervised autoencoders, given their potential for learning efficient and versatile embeddings. The architectures of these competing models are detailed in the Methods section and depicted in Fig 1b-c.

We utilized autoencoders based on the UNet architecture, given their previous successes in medical images processing *[24,25]*. The UNet framework was adapted for 3D image processing to handle volumetric brain MRI data effectively. The four architectures evaluated were:

1. **UNET**: A standard 3D U-Net autoencoder with skip connections. The first model was a "vanilla" 3D UNet autoencoder (UNET). This architecture maintains the traditional U-Net design with skip connections that allow information to bypass the bottleneck layer during reconstruction.
2. **U-AE**: A U-Net autoencoder without skip connections. Second, we reasoned that the UNet skip connections, while helpful in supervised segmentation tasks *[24]*, could be detrimental to the embedding quality in our application. We therefore derived a version of the UNET model without

skip connections (U-AE). This modification forces all information through the bottleneck layer, potentially creating more compressed and informative features.

3. **U-VAE**: A variational version of the U-AE model with reparameterization layer. Third, given published data suggesting that variational autoencoders can be useful for MR feature extraction *[26,27]*, we designed a variational version of the U-AE model (U-VAE), by including a reparameterization layer in the bottleneck section.
4. **VAE**: A fully connected variational autoencoder. Finally, we used a fully connected variational autoencoder (VAE) which successfully extracted features from CT images of lung lesions in a previous study *[18]*. This architecture provides a baseline comparison using a non-UNet based approach.

The encoder and decoder components were built using convolutional blocks, where each block repeated twice the following sequence: a 3D convolution layer (kernel=3, stride=1, padding=1), followed by batch normalization and ReLU activation (Fig 1c). Between these convolutional blocks, max pooling layers were used in the encoder while transposed convolution layers (kernel=2, stride=2, padding=0) were used in the decoder. As the input images progressed through the encoder, their dimensions were progressively reduced while the number of channels expanded in consecutive blocks (4, 8, 16, 32, 64 and 128). The bottleneck architecture differed between conventional and variational autoencoders: conventional autoencoders used a single convolutional block, while variational autoencoders employed two separate convolutional blocks followed by a reparameterization step. Each autoencoder generated two outputs: a vector of low-dimensional features at the bottleneck and a reconstructed version of the input image. We set the number of encoder/decoder blocks to 6, which yielded between 1,000 and 5,000 features per input image for conventional autoencoders, and exactly 2,048 features for variational autoencoders, dimensions suitable for subsequent machine learning tasks.

Autoencoder training algorithm

All deep learning models were implemented using the PyTorch library (version 2.0.0).

To prevent overfitting the model to normal healthy brains, only 800 randomly selected cases were finally kept from the UKB cohort for the model development, corresponding to 1,600 scans (T1 and FLAIR sequences). This strategic reduction from the full UKB cohort (19,955 individuals) was crucial to maintain balanced representation between healthy and pathological brain morphologies. Combined with glioma scans from UPENN (1,224 scans) and UCSF (990 scans), the final training dataset comprised 3,814 scans (T1,T1Gd, FLAIR; 80% training, 20% validation).

The training was carried out with a batch size of 14 images. For each batch, we aimed for the autoencoders to encode and reconstruct images as close to the original as possible. Therefore, we opted for a loss function relying on the structural similarity index measure between the input (x) and the output (ŷ) produced by the autoencoders *[28]* defined as follows (1):

$$(1)\ \ \mathrm{SSIM}(x,y)=\frac{\left(2\mu_x\mu_y+C_1\right)\left(2\sigma_{xy}+C_2\right)}{\left(\mu_x^2+\mu_y^2+C_1\right)\left(\sigma_x^2+\sigma_y^2+C2\right)}$$

with (μx, σx^2) and ($\mu \hat{y}$, $\sigma \hat{y}^2$) the respective pixel sample mean and variance of the input (x) and output (ŷ) images, $\sigma x\hat{y}$ the covariance of x and ŷ, and C_1 and C_2 two variables to stabilize divisions with weak denominators. As the SSIM ranges from 0 to 1, the autoencoders objective is thus to minimize the following loss function (2):

$$(2)\ \ \mathrm{SSIM_{loss}}=\frac{1}{\mathrm{n}}\sum_{\mathrm{i=1}}^{\mathrm{n}}1-\mathrm{SSIM}(\mathrm{x_i},\mathrm{y_i})$$

For variational autoencoders, the Kullback–Leibler divergence (KLD) loss (3) of the embedding distribution – that quantifies its divergence with the gaussian distribution – was added to the SSIM loss (4), with a weighting β parameter set to 10^{-4} *[29,30]*. The ADAM algorithm was used as the optimizer, with a learning rate of 10^{-4}.

$$(3)\ \mathrm{KLD_{loss}}=\sum_{\mathrm{i=1}}^{\mathrm{n}}\sigma_{\mathrm{i}}^2+\mu_{\mathrm{i}}^2-\log(\sigma_{\mathrm{i}})-1$$

$$(4)\ \ \mathcal{L}=\mathrm{SSIM_{loss}}+\beta\mathrm{KLD_{loss}}$$

Training was performed until convergence, with early stopping implemented to prevent overfitting.

Patient-level embedding generation

For each input MRI sequence (T1, T1Gd, or FLAIR), numerical characteristics (referred to as "features" throughout this manuscript) were extracted from the autoencoder's bottleneck layer. These sequence-specific features were then concatenated to generate comprehensive patient-level representations (referred to as "embeddings"), creating unified patient signatures that capture information from multiple MRI sequences while maintaining the distinct characteristics encoded by each sequence type.

To benchmark our approach, we also extracted radiomics features (excluding diagnostic features) from each sequence using the *PyRadiomics* library (v3.1.0) with whole brain masks for segmentation [31]. Radiomics served as an established reference method for medical image feature extraction, providing baseline comparison for evaluating the effectiveness of our deep learning-based approach. The radiomics features (1,132 per scan) were concatenated at the patient level following the same methodology as autoencoder features, enabling direct comparison between both feature extraction methods.

Statistical analysis and embedding evaluation

The quality and clinical relevance of the extracted embeddings were assessed through a comprehensive evaluation framework combining exploratory analyses, predictive modeling, and statistical comparisons.

Exploratory Analysis

UMAP analyses were computed on embeddings (2 components, 15 neighbors). Canonical correlations analysis (CCA) were performed between embeddings and clinical variables, which were summarized to 2 main components.

Predictive Modeling

Machine learning models were trained to predict clinical variables using the different generated embeddings. The model architecture was selected based on the type of clinical variable: Ridge regression for quantitative variables, L2-penalized logistic regression for categorical variables, and random survival forests for survival variables.

For each clinical variable, separate models were trained using embeddings from each autoencoder architecture and radiomics. We employed a 5-fold cross-validation procedure, with oversampling to address class imbalance in categorical variables.

Performance Evaluation

Hyperparameter optimization was performed using GridSearchCV within each fold to ensure optimal model configurations for each type of embedding.

The predictions from all validation folds were concatenated to generate ROC curves and compute performance metrics: weighted F1-scores for categorical variables, R^2 scores for quantitative variables, and C-indexes and Integrated Brier Scores for survival variables.

The predictive performance of models using different embeddings were compared using Wilcoxon signed-rank tests with Bonferroni correction for multiple comparisons.

Analyses were carried out with the scikit-learn library.

Synthetic data generation

To address datasets with limited sequence availability, we implemented a synthetic data enhancement strategy. A dedicated UNet-based model was trained to perform bidirectional synthesis between T1 and FLAIR sequences.

The synthetic data generation model utilized a standard 3D U-Net autoencoder architecture (with skip connections). The model consisted of 6 encoder/decoder blocks with progressive feature expansion (4, 8, 16, 32, 64, and 128 channels), processing single-channel inputs (T1 or FLAIR sequences independently). Each convolutional block comprised two sequential 3D convolution layers (kernel=3, stride=1, padding=1) followed by batch normalization, ReLU activation, and dropout (rate=0.1). Max pooling layers (2x2x2) were used for downsampling in the encoder, while transposed convolution layers (kernel=2, stride=2, padding=0) performed upsampling in the decoder.

The model was trained on the UKB dataset using 39,910 sequences (T1 and FLAIR pairs) with an 80/20 train/validation split, to establish bidirectional correspondence between T1 and FLAIR sequences, enabling the model to learn both T1→FLAIR and FLAIR→T1 synthesis. The training objective utilized SSIM loss to optimize structural similarity between synthethic and corresponding real images, with the ADAM optimizer and learning rate of $5x10^{-5}$.

Data availability

Clinical and imaging data (DICOM and NIFTI files) were collected from publicly available datasets: the UKB (data use agreement, Application Number 51998) healthy volunteer dataset [32,33], the MCIC and COBRE schizophrenia datasets [34–37], and the ADNI Alzheimer dataset. Glioma datasets were downloaded from The Cancer Imaging Archive [38,39]: UCSF-PDGM *[40]*, UPenn-GBM [41,42], TCGA-LGG[43] and TCGA-GBM [44,45].

ADNI data access followed standard protocols through the ADNI database [46]. Glioma datasets from The Cancer Imaging Archive were accessed according to their respective data use policies.

The source codes of the different autoencoders presented in this manuscript are available on GitHub. The complete DUNE workflow is registered in WorkflowHub (doi:10.48546/workflowhub.workflow.1809.1), bio.tools (biotools:GevaertLab_DUNE), and SciCrunch (RRID:SCR_027208). All preprocessing scripts, model implementations, and evaluation code necessary to reproduce the main findings of this study are publicly available through the GitHub repository. The data and code required to reproduce the figures are available at FigShare repository [47]. Processed embeddings and intermediate analysis results are available upon reasonable request to the corresponding author. All additional supporting data is available in the GigaScience repository, GigaDB [48].

Availability of Source Code and Requirements

Project name: DUNE
Project homepage: https://github.com/gevaertlab/DUNE
License: Apache-2.0 license
SciCrunch RRID:SCR_027208
bio.tools ID: GevaertLab_DUNE

System requirements
Operating system: Platform independent
Programming language: Python
Package management: pip
Hardware requirements: 16GB+ RAM, GPU with 16GB+ VRAM, CPU multicore processor (8+ cores recommended)

RESULTS

Model reconstruction performance comparison

We compared the reconstruction capabilities of the four candidate architectures for *DUNE's* feature extraction module. All four models trained to encode and reconstruct single-sequence 3D brain MRI scans (Fig 1d). Reconstruction performance varied significantly across architectures (Fig 1d). UNET achieved the highest SSIM scores (98.7 ±4.2%) across all sequences (T1, T1Gd, FLAIR) for both cancer and non-cancer images. U-VAE and VAE achieved SSIM scores of 82.6 ±4.5% and 84.4 ±2.8% respectively for general reconstruction but showed poor performance on brain tumor reconstruction. U-AE achieved SSIM scores of 72.2 ±4.2%.

However, since our primary objective was to generate clinically meaningful embeddings rather than achieve optimal reconstruction, we next evaluated the quality of bottleneck-derived features for downstream clinical prediction tasks.

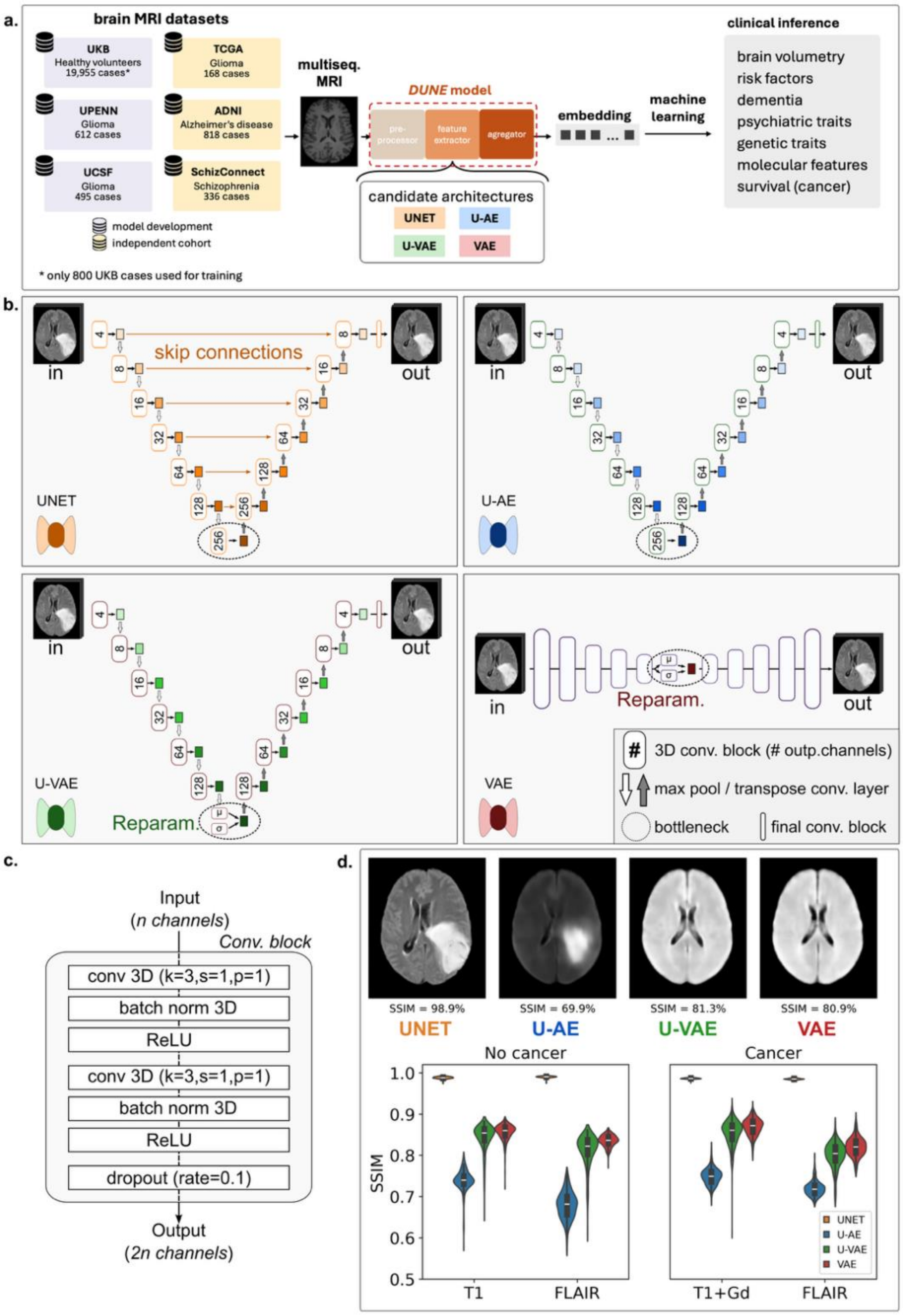

Figure 1 – Project overview and model development.

a) Workflow for the development and evaluation of the DUNE model. The development of the model involved three distinct datasets: UKB, UPENN, and UCSF. The DUNE model trained to encode and reconstruct full-sized brain MRI scans, thereby generating embeddings that capture the salient information from the original images. Subsequently, embeddings were generated for scans from the development datasets as well as three independent cohorts (TCGA, ADNI, SchizConnect). These compact representations were then utilized as inputs to simple machine learning models for the inference of clinical variables, allowing for an assessment of the embeddings' quality and their ability to facilitate downstream clinical applications.

b) We benchmarked four distinct candidate architectures for DUNE: UNET, U-AE, U-VAE, and VAE. Three of the models (UNET, U-AE, and U-VAE) are based on the U-Net architecture, while VAE is a variational autoencoder model adopted from a previous study[18]. Specifically, UNET is the foundational U-Net model, U-AE is a variant without skip connections, and U-VAE is a variational version of U-AE that generates embeddings through a reparameterization procedure.

c) The models are composed of sequential convolutional blocks and max pooling layers that reduce the dimension of brain MRI to generate the embeddings.

d) The base UNET model demonstrated the highest similarity (SSIM) between the output and original images. While the U-AE generated less precise images, it appeared to better identify cancer characteristics compared to variational autoencoders, which failed to reconstruct the tumor.

MRI embeddings accurately predict clinical phenotypes of healthy individuals

Clinical variables and study design

To identify the optimal architecture for DUNE among the four candidates, we conducted a comprehensive evaluation based on the quality of the embeddings they produce. As a first assessment criterion, we measured how well these embeddings correlated with clinical variables (Fig 2). We used all four candidate models to encode the images from the UKB dataset, with radiomics serving as a control given its established role as a state-of-the-art method for image feature extraction*[31]*. For each patient, we generated global brain MR embeddings by concatenating features extracted from both T1 and FLAIR sequences.

We analyzed these embeddings against 284 clinical variables hypothesized to be associated with brain morphology. These variables were categorized into seven distinct groups: acquisition parameters (A), brain volumetry (Vo), demographics (D), vascular risk factors (Va), toxics (T), psychiatric (P) and genetic (G) traits (Fig 2a and b). The variables showed diverse statistical distributions, with some quantitative variables such as volumetric measurements, age, and carotid thickness following normal distributions (Fig 2b), while certain qualitative variables, notably APOE4 status, exhibited significant class imbalance. Our correlation analyses revealed strong associations within each category but weak correlations between categories (Supplemental Figure S2), leading us to analyze each group independently.

Exploratory analysis

UMAP analyses of the embeddings revealed distinct patterns (Fig 2c). The variational autoencoders (U-VAE and VAE) produced normally distributed embeddings, as expected given their underlying statistical constraints. Embeddings from radiomics, U-AE, and to a lesser extent UNET, showed clear relationships with key clinical parameters, evidenced by distinct color patterns corresponding to acquisition parameters (scanner table position), volumetric measurements (cerebrospinal fluid volume), and demographic features (age and sex). In contrast, the embeddings from variational autoencoders (VAEs) showed no discernible patterns in relation to these clinical variables.

To assess the overall relationship between embeddings and clinical variables, we performed CCA on each clinical variable group. Figure 2d displays the determination coefficients (R^2 scores) between the first canonical variates of the embeddings and that of each subgroup of clinical variables. The total area covered by these coefficients in the radar plot summarizes the overall correlation strength. Using this metric, U-AE embeddings demonstrated the strongest correlation with clinical variables (area=1.81), outperforming the radiomics features (area=1.48), which ranked second.

Clinical prediction tasks

As a second evaluation criterion, we assessed how well each type of embedding could serve as input features for clinical prediction tasks. To do this, we trained supervised machine learning models to predict each of the 284 clinical variables from the UKB dataset. For each variable, models were trained on embeddings from each architecture (UNET, U-AE, U-VAE and VAE) or radiomics (RAD), used as control. The performance of clinical prediction was measured by the R^2 score (quantitative variables) or the weighted F1-score (categorical variables). The results are displayed in Fig 3a (overall performance, and per variable subgroups).

Clinical prediction performance varied across embedding types (Fig 3a). U-AE embeddings achieved prediction scores of 0.384±1.63%, compared to radiomics (0.145±9.7%, $p<0.001$). UNET, U-VAE, and VAE embeddings achieved lower prediction scores across clinical variables. The performances greatly varied between the different subgroups of clinical variables but showed small variation across variables within each category (Fig 3a), which tended to be highly intercorrelated (Supplemental Figure S2). Figure 3b to 3h thus display the performance of model predictions for illustrative variables from each category. U-AE embeddings were the most relevant in predicting most clinical variables. Embeddings encoded by variational autoencoders (U-VAE and VAE) were outperformed in all categories, except genetics.

Based on these comprehensive evaluations, U-AE was identified as the optimal feature extraction architecture for DUNE in morphologically normal brains, demonstrating superior embedding quality despite its poor sequence-level reconstruction performance.

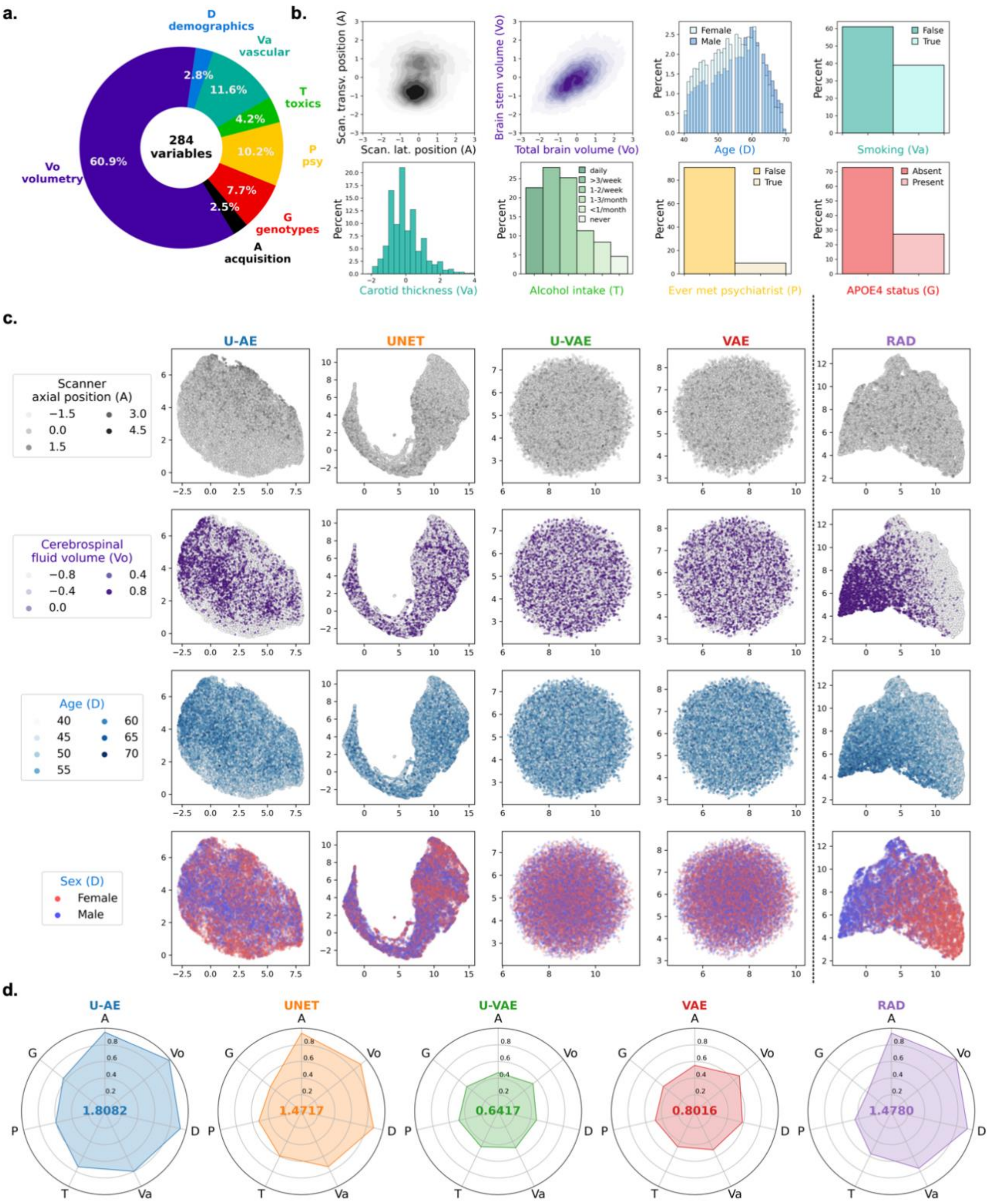

Figure 2 – MRI embeddings correlate with clinical phenotypes in healthy individuals

a) 284 clinical parameters collected in the UKB dataset were divided into 7 categories (Vo: volumetry, A: acquisition protocol, G: genotypes, P: psychiatric traits, T: toxics, Va: vascular conditions, D: demographics).

b) Distribution of illustrative variables from the different categories.

c) UMAP reduction was performed on embeddings extracted from brain MR images by the 4 models and on radiomics features as control. Radiomics, UNET and U-AE generated embeddings successfully capture variables of acquisition, volumetry and demographic categories, while those issued by variational autoencoders do not.

d) Canonical correlation analysis (CCA) between embeddings and clinical variables from each category. The radar plots display the correlation (R^2 metric) between the first canonical variate of each category and that of each embedding, showing that U-AE embeddings correlate best with UKB clinical parameters.

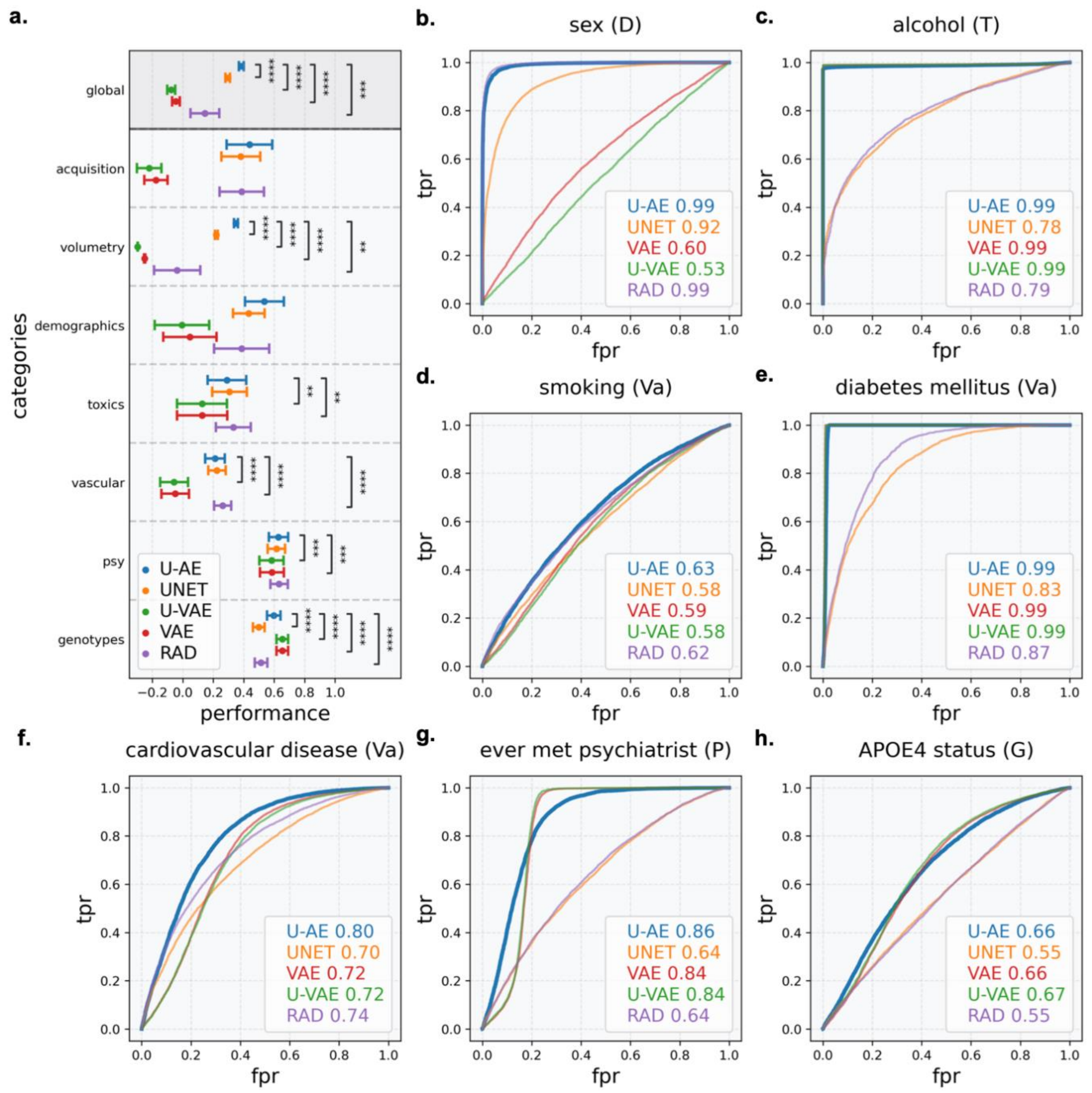

Figure 3 - Clinical inference using MRI embeddings of healthy brains.

a) Performance of machine learning models trained to predict clinical variables using model-generated embeddings of the UKB brain MRIs (T1 and FLAIR sequences, 19,955 cases, 5-fold cross validation). Points and error bars show means and standard errors of prediction scores (R^2 or F1-scores) across variables in each category. Overall, U-AE embeddings yielded the best predictions, except for toxics, genotypes and vascular conditions that were best predicted by radiomics and variational autoencoders respectively

b) to h) Individual performance of clinical inference based on either autoencoder embeddings or radiomics.

MRI embeddings capture cancer-specific features including molecular features and predict patient survival

Having validated our models on healthy brains, we next investigated their generalization capabilities to pathological cases. Specifically, we first focused on glioma patients, whose brain MRIs exhibit significant morphological abnormalities due to tumor presence. For this analysis, we used two categories of datasets: the UCSF and UPENN datasets, which were part of the autoencoder training set, along with the previously unseen TCGA-GBM and TCGA-LGG datasets. Once more, we assessed the quality of the embeddings in oncological context, by developing survival prediction models using them as input. For evaluation, we stratified the patients into two groups based on their predicted mortality risk (above or below median) and compared their survival curves. The prediction quality was measured by two complementary metrics: the Brier score, which measures prediction accuracy (optimal when close to 0), and the concordance index (C-index), which measures ranking accuracy (optimal when close to 1).

Models using U-AE encoded embeddings demonstrated superior performance in discriminating between low and high-risk patients. This superiority was consistent across both internal validation datasets, with C-index=0.587 and Brier score=0.184 for UPENN, and C-index=0.686 and Brier score=0.204 for UCSF, outperforming both radiomics features and alternative encodings from UNET and VAE architectures (Fig 4a and b). The external validation on the TCGA dataset confirmed these findings, with U-AE embeddings achieving better survival predictions (C-index=0.608) compared to radiomics (C-index=0.600) and other autoencoder-based approaches (Fig 4c).

Beyond survival prediction, we evaluated the embeddings' ability to predict key molecular characteristics of gliomas that guide therapeutic decisions. These included IDH1 mutation status and MGMT promoter methylation status, which are established markers of response to chemoradiation and temozolomide-based chemotherapies (Fig 5a and b). The U-AE embeddings consistently demonstrated superior predictive performance, achieving high accuracy in detecting IDH1 mutations (AUROC=0.99 in UCSF and AUROC=0.92 in TCGA), MGMT promoter methylation status (AUROC=0.77), and tumor grade (AUROC=0.94). Notably, even in these morphologically complex cases, the embeddings maintained their ability to predict general anatomical features such as patient sex (data not shown).

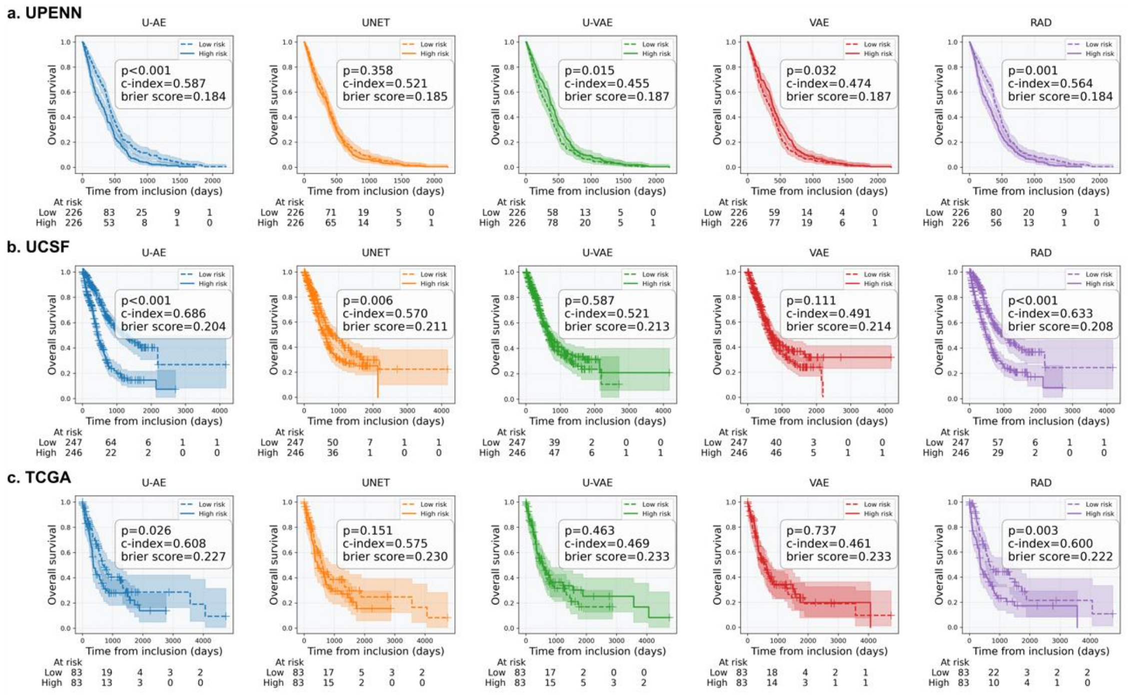

Figure 4 – MRI embeddings allow for survival inference in individuals with glioma

Performance of random forest survival models predicting patient global survival based on MR embeddings/radiomics (T1Gd and FLAIR sequences) of 3 glioma datasets (5-fold cross validation). The overall survival of two groups, defined by risk scores of death below (low risk) or above (high risk) median, was compared (concordance index and log-rank test), for the UPENN (a) and UCSF (b) datasets (which were used as train sets for the autoencoders), and for the external TCGA (c) dataset. Predictions using the U-AE embeddings had the highest concordance index for all three datasets.

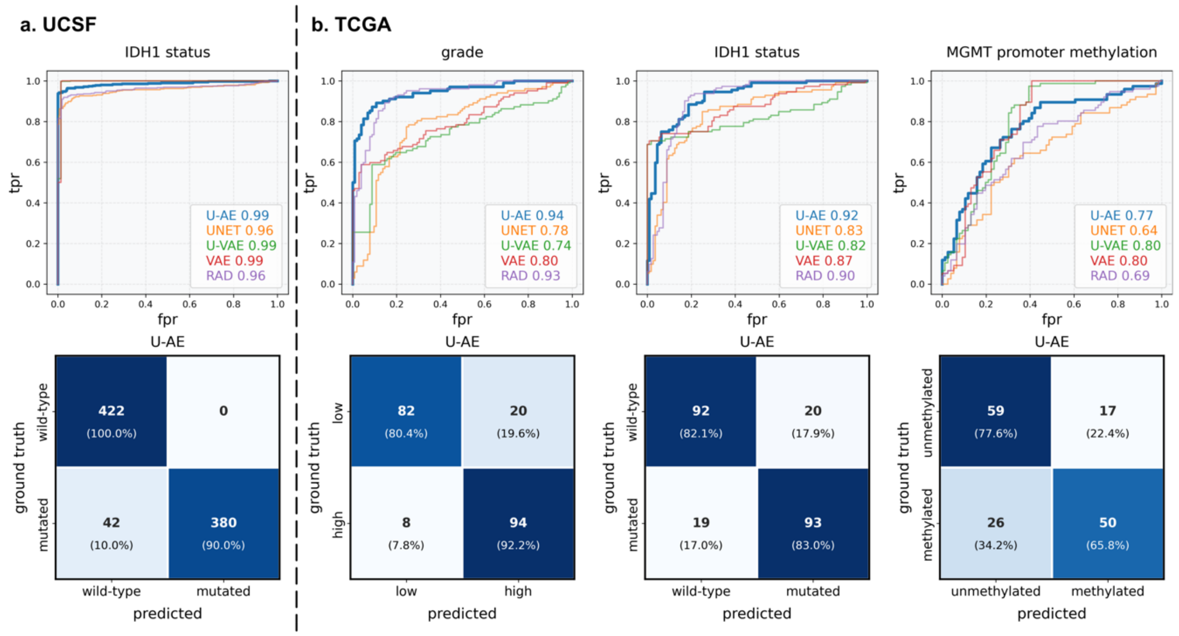

Figure 5 – MRI embeddings enable inference of molecular features in patients with glioma. Performance of models predicting molecular features using MR embeddings/radiomics (T1Gd and FLAIR sequences) of the UCSF (a, IDH1 status) and TCGA datasets (b, cancer grade, IDH1 status, MGMT promoter methylation status).

MRI embeddings predict diagnosis of neurodegenerative and psychiatric disorders

We continued the benchmarking of the different models with two additional neurological conditions: neurodegenerative disease and schizophrenia. For this analysis, we leveraged data from the ADNI database for Alzheimer's disease and the SchizConnect database (comprising COBRE and MCIC datasets). Unlike our previous analyses, these datasets contained only T1-weighted images, allowing us to test our models' performance with limited imaging sequences.

In the Alzheimer's disease cohort, we first evaluated the models' ability to predict cognitive impairment severity. The U-AE embeddings demonstrated robust performance with an AUROC of 0.92 (Fig 6a). We then evaluated the prediction of genetic markers known to influence Alzheimer's disease progression: the APOE4 allele status and the TOMM40 poly-T polymorphism length (dichotomized at the cohort median of 33). Interestingly, for these genetic predictions, the variational autoencoders performed the best. The U-VAE and VAE embeddings achieved perfect prediction for the APOE4 allele (AUROC=1) and moderately high accuracy for TOMM40 polymorphism length (AUROC=0.67 and 0.71, respectively), compared to the U-AE embeddings (AUROC=0.98 for APOE4 and AUROC=0.68 for TOMM40) (Fig 6a).

In the schizophrenia cohort, we assessed the models' performance across four distinct prediction tasks: identification of study origin (COBRE vs MCIC, which reflected differences in MR acquisition protocols), clinical diagnosis (schizophrenia vs healthy volunteers), and demographic characteristics (sex and age). The U-AE embeddings maintained their superior performance, perfectly discriminating between studies of origin (AUROC=1) and accurately predicting patient sex (AUROC=0.97). For schizophrenia diagnosis, both U-AE embeddings and radiomics features achieved meaningful predictive performance (AUROC=0.64 and 0.72 respectively) (Fig 6b).

MRI embeddings can be improved by synthetic data

To address the limitation of single-sequence availability in the ADNI and SchizConnect datasets, we developed an enhancement strategy using synthetic data generation. We applied a synthetic FLAIR generation model to both the SchizConnect and ADNI datasets, generating FLAIR sequences from their available T1 images. This approach proved highly effective, with synthetic FLAIR images showing strong concordance with real FLAIR images in the UKB validation set (similarity index = 0.90 ± 0.04). We then applied this synthetic data enhancement to both the SchizConnect and ADNI datasets, generating FLAIR sequences from their available T1 images. This strategy enabled us to compare the predictive performance between embeddings derived from T1 images alone and those generated from the combination of T1 and synthetic FLAIR images, using our established clinical parameters.

The integration of synthetic FLAIR images yielded consistent improvements in prediction performance across all analyses. In the ADNI dataset, embeddings combining T1 and synthetic FLAIR

sequences demonstrated enhanced predictive capability compared to those derived from T1 sequences alone (0.756±0.14 vs 74.5±0.14 with U-AE embeddings; Fig 6c top). We observed similar improvements in the SchizConnect dataset, where the addition of synthetic FLAIR sequences enhanced the predictive performance of the embeddings (0.777±18.9% vs 0.750±0.21 with U-AE embeddings; Fig 6c bottom). Remarkably, this enhancement in performance was consistent across all autoencoder architectures, suggesting that the benefit of incorporating synthetic FLAIR sequences extends beyond any specific model implementation.

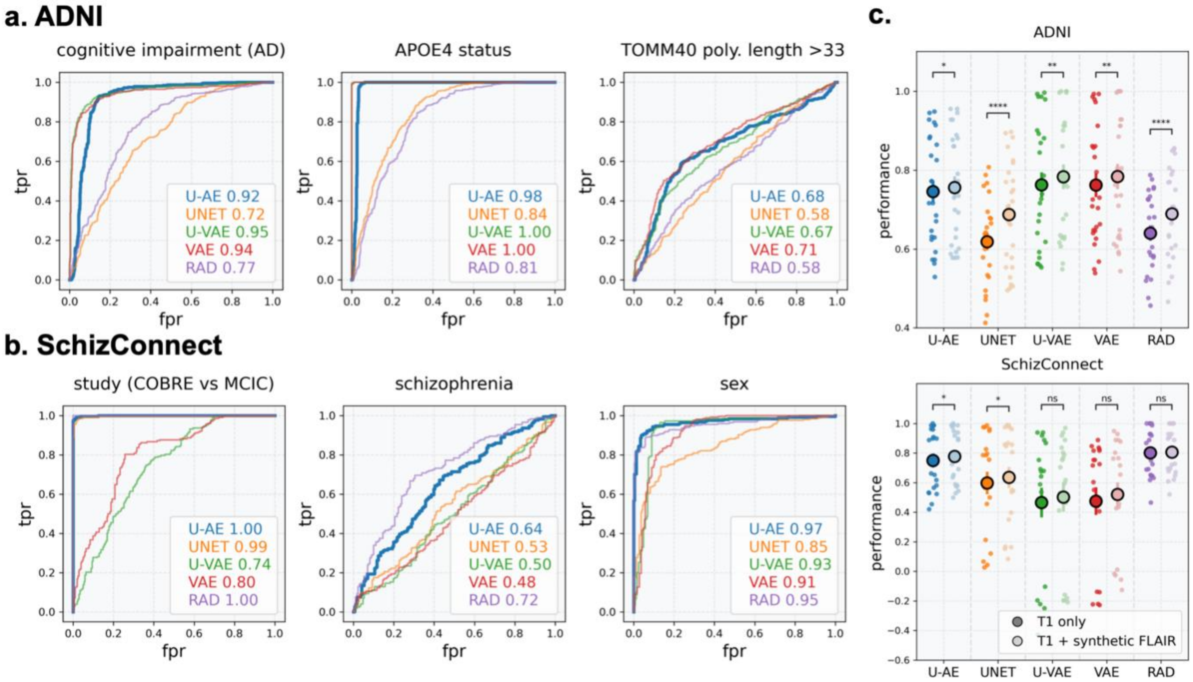

Figure 6 - Clinical inference in Alzheimer's disease and schizophrenia.

a) Performance of models predicting clinical features (cognitive impairment [MMSE>28]) and genotypes (APOE4 status and length of the TOMM40 poly-T polymorphism [>33]) using MR embeddings/radiomics of the ADNI dataset (T1 sequences).

b) Performance of models predicting parameters and clinical variables (cohort of origin [COBRE vs MCIC], schizophrenia vs healthy patients, sex and age) using MR embeddings/radiomics of the SchizConnect dataset (T1 sequences).

c) Performance of models predicting clinical parameters from ADNI (top) and SchizConnect (bottom) datasets based on MR embeddings/radiomics of either T1 only images (dark dots) or both T1 + synthetic FLAIR images (light dots).

DISCUSSION

We introduce *DUNE*, a complete workflow that generates versatile and comprehensive embeddings from brain MRI scans for downstream analyses. DUNE's feature extractor was developed through systematic benchmarking of four autoencoder architectures. By evaluating these relatively simple architectures on diverse prediction tasks, we identified that a skip-connection-free UNet autoencoder (U-AE) consistently generates the most informative embeddings from brain MRIs, and was therefore selected as the final architecture for DUNE. Surprisingly, this architecture demonstrated the poorest reconstruction capabilities while producing the most clinically relevant embeddings, highlighting a counter-intuitive finding that reconstruction performance does not predict embedding quality for clinical applications. We further validated this principle through additional experiments with enhanced loss functions incorporating gradient-based detail preservation constraints, which confirmed improved reconstruction quality at the cost of reduced clinical prediction performance (data not shown). This trade-off represents an important consideration in autoencoder architectures, where the removal of skip connections forces all information through the bottleneck layer, creating more informative representations despite reduced pixel-level accuracy.

The versatility of DUNE's embeddings was demonstrated across multiple levels of clinical prediction. When used with simple machine learning models, they enabled accurate prediction of basic parameters such as brain volumetry and demographic features (age, sex), while simultaneously capturing complex molecular and genetic signatures. In neuro-oncology, they achieved robust prediction of IDH1 mutation status, matching the performance of sophisticated deep learning models that process whole MR images

directly [49–51]. In neurodegenerative diseases, they successfully identified genetic traits such as APOE4 allele status, and in psychiatric disorders, they effectively detected subtle brain alterations associated with disease status.

Among the candidate architectures we evaluated, UNet-based autoencoders were initially selected for their relative simplicity and proven success in medical image processing *[52]*. We hypothesized that such models could generate embeddings that faithfully capture the fundamental features of brain MRI structures. Unlike conventional applications, we employed unsupervised training through image reconstruction to optimize embedding versatility. Our systematic evaluation revealed that while the standard UNet architecture excelled at image reconstruction, its skip connections, which allow information to bypass the bottleneck layer, resulted in suboptimal embeddings. The removal of these skip connections in our U-AE model forced all information through the bottleneck, producing more informative embeddings despite lower reconstruction quality. We also explored variational autoencoders based on previous work [23,53–55], but these were outperformed by traditional architectures and even radiomics, despite careful tuning of the Kullback-Leibler divergence term in the loss function.

Recent advances in computer vision have highlighted vision transformers as powerful tools for image analysis *[56,57]*. Their self-attention mechanisms enable sophisticated spatial reasoning by weighing the importance of different image regions, which could theoretically benefit brain MRI analysis (1–5). However, several considerations led us to favor autoencoders for this application. First, while vision transformers excel at classification tasks, they are not inherently designed for dimensionality reduction and feature extraction, which was our primary objective. Second, the traditional autoencoder architecture provides an interpretable bottleneck structure that naturally aligns with our goal of generating compact, meaningful embeddings. Third, vision transformers typically require extensive pre-training on large-scale datasets to achieve optimal performance, making them less suitable for specialized medical imaging applications where data availability is limited. For 3D brain MRI analysis specifically, the computational and data requirements of transformer architectures become prohibitive given the high complexity of volumetric data and the relatively modest size of available medical imaging datasets. Finally, autoencoders offer better computational efficiency and have demonstrated superior adaptability to smaller, domain-specific datasets *[60]*. This latter point was particularly crucial for our application, as it enabled us to train effectively on our relatively modest collection of brain MRI scans while maintaining model stability and generalization capability.

A major challenge in developing MRI analysis models is the heterogeneous nature of image acquisition across different centers. This heterogeneity makes image normalization particularly challenging and often leads to models that overfit to their training dataset and generalize poorly to external data. We addressed this challenge through two key strategies. First, we implemented a comprehensive image standardization pipeline, enabling our models to effectively learn from multiple datasets while maintaining robustness to external data. Second, and more importantly, we identified that maintaining a balanced representation of different brain morphologies in the training data was crucial. Initially, training with the complete UKB cohort (≈20,000 cases) alongside the glioma datasets led to a bias towards normal brain morphology, resulting in poor-quality embeddings for tumor cases. We resolved this issue by downsampling the UKB dataset to match the size of the glioma datasets, which improved the quality of tumor embeddings while preserving the model's performance on normal brain images (data not shown).

The processing of multiple MRI sequences represented another key optimization challenge in our model development. Initially, we explored a multi-channel approach where different MR sequences were concatenated and processed simultaneously, similar to how RGB channels are handled in natural image processing. However, our systematic evaluation revealed superior performance with an alternative strategy: processing each MR sequence independently through separate encoding paths and subsequently concatenating their extracted features. This sequential approach not only improved the quality of the extracted features but also provided greater operational flexibility, allowing the model to handle incomplete imaging datasets where certain sequences might be unavailable. Such flexibility is particularly valuable in clinical settings, where the availability of multiple MRI sequences cannot always be guaranteed.

Our exploration of synthetic data enhancement revealed promising opportunities for improving embedding quality. We explored several strategies to enhance the quality of our embeddings through data augmentation. Our initial attempt to combine autoencoder-generated embeddings with radiomics features showed no improvement in downstream clinical predictions compared to either approach alone (data not shown). However, we discovered that incorporating synthetic MRI sequences, generated through deep learning, significantly enhanced embedding quality.

The success of synthetic data enhancement likely stems from the complementary information encoded across MRI sequences. T1-weighted images primarily capture anatomical structures and tissue contrast, while FLAIR sequences enhance visualization of pathological changes and cerebrospinal fluid suppression. By generating the missing modality, the multimodal nature of embeddings was restored, enabling single-sequence datasets to leverage the enhanced representational capacity demonstrated by dual-sequence embeddings in our initial validation.

This finding is particularly relevant in the current context of medical imaging, where there is increasing pressure to optimize MRI protocols by reducing acquisition times and sequences. Our results suggest that synthetic imaging could help maintain comprehensive analyses while limiting the actual scanning time. Moreover, this success with synthetic MRI data opens promising perspectives in the broader context of generative AI. Similar approaches could be developed to generate synthetic data across other modalities such as transcriptomics or pathology images [61–64,17]. Such synthetic data generation could provide a powerful strategy to enhance the quality of medical imaging embeddings by incorporating complementary information from multiple modalities, even when the original data is not available.

Literature is scarce regarding models that allow unsupervised feature extraction from brain MRI, with rare applications in cancer or psychiatric disorders *[65,66]*. Therefore, radiomics – first coined by Lambin et al. as an innovative algorithm allowing the high-throughput extraction of medical images *[67]* – has remained the best way to extract features from radiological images thus far. The radiomics workflow includes a segmentation step followed by the extraction of multiple quantitative features consisting in intensity distribution, spatial relationships between intensity levels, texture patterns and shape descriptors. The most informative of these features are automatically selected from specific criteria, and can further be used in downstream tasks. The robustness of radiomics makes it applicable to multiple imaging modalities in a broad range of applications, particularly in cancer [68–70]. In contrast to radiomics, which extract quantitatively measurable features, deep learning models extract more abstract and higher-level features that are expected to generate more comprehensive embeddings. Indeed, in the current study, the embeddings generated by our model generated better predictions than that based on radiomics.

The potential applications of DUNE extend to the emerging field of data fusion, where features from multiple data sources are combined to enhance prediction accuracy. Recent studies have demonstrated the power of this approach, showing how the integration of pathology and transcriptomics data can improve brain cancer prognosis prediction beyond what either modality can achieve alone *[71]*. In this context, DUNE could provide a robust framework for incorporating radiological data into such multi-modal analyses, potentially further improving predictive performance.

While our results demonstrate DUNE's broad utility, several limitations should be acknowledged. Currently, the model is optimized for three specific MRI sequences: T1, T1Gd, and FLAIR. Although additional sequences, such as diffusion-weighted imaging, could be incorporated given sufficient training data, this represents a current constraint on the model's applicability. Furthermore, unlike the more versatile radiomics approach which can analyze any radiological image, DUNE is specifically designed for brain MRI analysis. However, the underlying architecture is fundamentally capable of processing any 3D medical image, suggesting that organ-specific versions could be developed for other clinical applications.

Another potential limitation is the risk for dataset-specific confounding, as certain diseases are primarily represented within specific institutional cohorts, potentially leading to embeddings capturing technical rather than pathological characteristics. However, we implemented strategies to mitigate this risk, and multiple lines of evidence suggest that DUNE embeddings capture biological rather than technical features. First, we employed the ANTs-based preprocessing pipeline, which harmonizes images across different acquisition protocols and scanner types, thus reducing technical variability. Additionally, DUNE successfully predicts fundamental biological features (for example sex, IDH1 or APOE4 status) across different datasets, and demonstrates robust performance on external validation cohorts acquired using different protocols than training datasets (TCGA, ADNI, SchizConnect). If embeddings primarily encoded scanner-specific artifacts, we would expect degraded performance on these cross-institutional predictions, which we do not observe.

In conclusion, DUNE represents a significant advance in medical image analysis, capable of extracting comprehensive compact representations from complex brain MRI scans. These embeddings effectively capture both obvious and subtle imaging features while maintaining clinical relevance. By facilitating more accurate diagnoses, refined prognostic assessments, and better-informed therapeutic decisions, DUNE contributes to the advancement of precision medicine in neurology. The model's ability to generate meaningful embeddings from standard clinical imaging sequences, combined with its

potential for integration into multi-modal analysis frameworks, positions it as a valuable tool for developing more personalized and effective patient care strategies.

SUPPLEMENTARY MATERIALS

Name	Sources/datasets	Population	Sequences	No of cases
UKB	UK Biobank	General population	T1, FAIR	19,955
UPENN	University of Pennsylvania glioblastoma dataset	Glioma (GBM only)	T1Gd, FLAIR	612
UCSF	UCSF preoperative diffuse glioma MRI dataset	Glioma	T1Gd, FLAIR	495
TCGA	TCGA-LGG and TCGA GBM datasets	Glioma	T1Gd, FLAIR	168
ADNI	Alzheimer's Disease Neuroimaging Initiative 1 dataset	Alzheimer and HV	T1	818
SchizConnect	SchizConnect database (COBRE and MCIC datasets)	Schizophrenia and HV	T1	336

Supplemental Table S1 - Brain MR datasets used in the study.
Images of the UKB, UPENN and UCSF datasets were used to train the models. HV: healthy volunteers; QA: quality assessment. T1Gd: T1 + gadolinium.

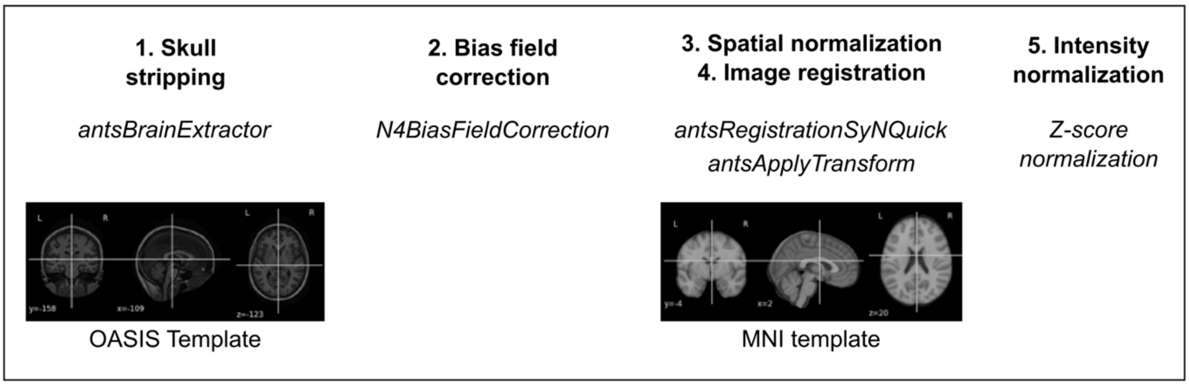

Supplemental Figure S1 - Brain MR preprocessing steps

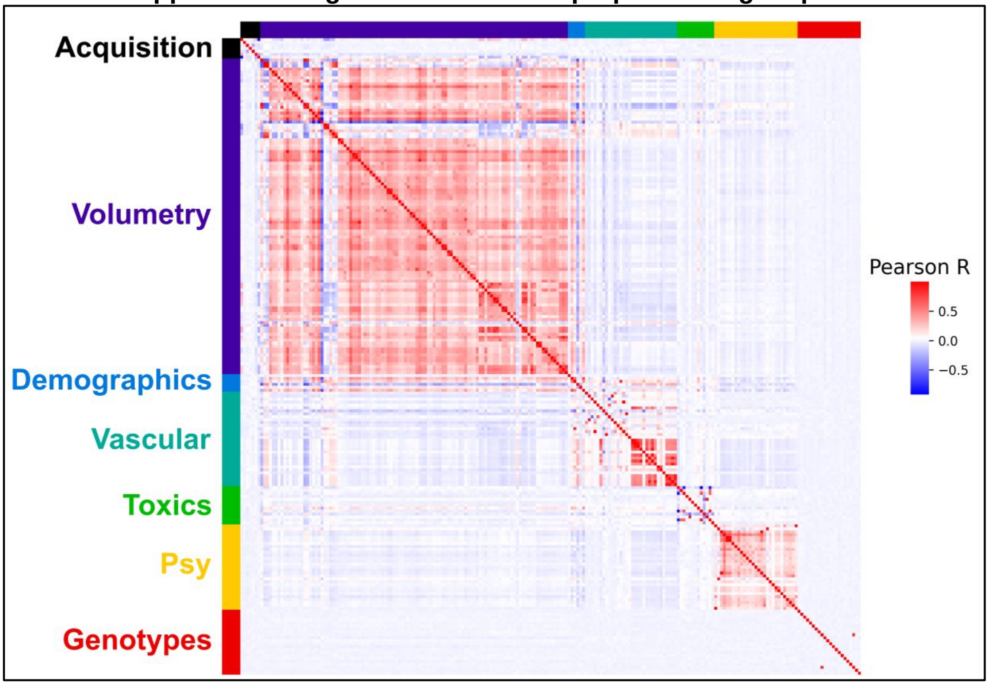

Supplemental Figure S2 - Correlation matrix of UKB clinical variables

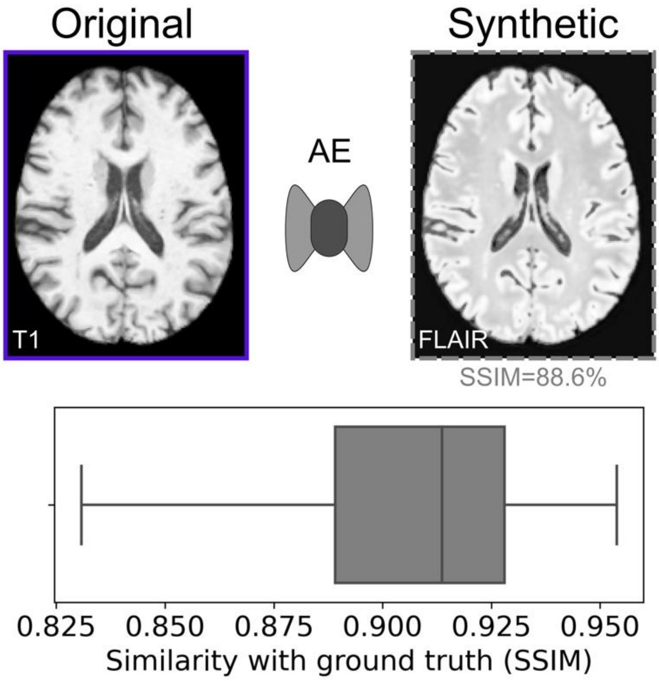

Supplemental Figure 3 - Generation of synthetic FLAIR from T1

REFERENCES

1. Lenroot RK, Giedd JN. The changing impact of genes and environment on brain development during childhood and adolescence: Initial findings from a neuroimaging study of pediatric twins. Dev Psychopathol. 2008; doi: 10.1017/S0954579408000552.
2. Song R, Xu H, Dintica CS, Pan K-Y, Qi X, Buchman AS, et al.. Associations Between Cardiovascular Risk, Structural Brain Changes, and Cognitive Decline. J Am Coll Cardiol. 2020; doi: 10.1016/j.jacc.2020.03.053.
3. Gamal RM, Abozaid HSM, Zidan M, Abdelmegid MA-KF, Abdel-Razek MR, Alsayed SA-R, et al.. Study of MRI brain findings and carotid US features in systemic sclerosis patients, relationship with disease parameters. *Arthritis Research & Therapy*. 2019; doi: 10.1186/s13075-019-1877-z.
4. Kaichi Y, Kakeda S, Moriya J, Ohnari N, Saito K, Tanaka Y, et al.. Brain MR Findings in Patients with Systemic Lupus Erythematosus with and without Antiphospholipid Antibody Syndrome. *AJNR Am J Neuroradiol*. 2014; doi: 10.3174/ajnr.A3645.
5. Hemond CC, Bakshi R. Magnetic Resonance Imaging in Multiple Sclerosis. *Cold Spring Harb Perspect Med*. 2018; doi: 10.1101/cshperspect.a028969.
6. van Oostveen WM, de Lange ECM. Imaging Techniques in Alzheimer’s Disease: A Review of Applications in Early Diagnosis and Longitudinal Monitoring. *International Journal of Molecular Sciences*. Multidisciplinary Digital Publishing Institute; 2021; doi: 10.3390/ijms22042110.
7. Gevaert O, Mitchell LA, Achrol AS, Xu J, Echegaray S, Steinberg GK, et al.. Glioblastoma multiforme: exploratory radiogenomic analysis by using quantitative image features. *Radiology*. 2014; doi: 10.1148/radiol.14131731.
8. Itakura H, Achrol AS, Mitchell LA, Loya JJ, Liu T, Westbroek EM, et al.. Magnetic resonance image features identify glioblastoma phenotypic subtypes with distinct molecular pathway activities. *Sci Transl Med*. 2015; doi: 10.1126/scitranslmed.aaa7582.
9. Shin I, Kim H, Ahn SS, Sohn B, Bae S, Park JE, et al.. Development and Validation of a Deep Learning–Based Model to Distinguish Glioblastoma from Solitary Brain Metastasis Using Conventional MR Images. *AJNR Am J Neuroradiol*. 2021; doi: 10.3174/ajnr.A7003.
10. Zhang J, Rao VM, Tian Y, Yang Y, Acosta N, Wan Z, et al.. Detecting schizophrenia with 3D structural brain MRI using deep learning. *Sci Rep*. Nature Publishing Group; 2023; doi: 10.1038/s41598-023-41359-z.
11. Feng X, Provenzano FA, Small SA, for the Alzheimer’s Disease Neuroimaging Initiative. A deep learning MRI approach outperforms other biomarkers of prodromal Alzheimer’s disease. *Alzheimer’s Research & Therapy*. 2022; doi: 10.1186/s13195-022-00985-x.

12. Cawley GC, Talbot NLC. On Over-fitting in Model Selection and Subsequent Selection Bias in Performance Evaluation. *J Mach Learn Res*. 11:2079–1072010;

13. Bommasani R, Hudson DA, Adeli E, Altman R, Arora S, von Arx S, et al.. On the Opportunities and Risks of Foundation Models. arXiv; 2021; doi: 10.48550/arXiv.2108.07258

14. Zhou C, Li Q, Li C, Yu J, Liu Y, Wang G, et al.. A Comprehensive Survey on Pretrained Foundation Models: A History from BERT to ChatGPT. arXiv; 2023; doi: 10.48550/arXiv.2302.09419

15. Hinton GE, Salakhutdinov RR. Reducing the Dimensionality of Data with Neural Networks. *Science*. American Association for the Advancement of Science; 2006; doi: 10.1126/science.1127647.

16. Qiu YL, Zheng H, Gevaert O. Genomic data imputation with variational auto-encoders. *Gigascience*. 2020; doi: 10.1093/gigascience/giaa082.

17. Carrillo-Perez F, Pizurica M, Ozawa MG, Vogel H, West RB, Kong CS, et al.. Synthetic whole-slide image tile generation with gene expression profile-infused deep generative models. *Cell Rep Methods*. 2023; doi: 10.1016/j.crmeth.2023.100534.

18. Li Y, Sadée CY, Carrillo-Perez F, Selby HM, Thieme AH, Gevaert O. A 3D lung lesion variational autoencoder. *Cell Reports Methods*. 2024; doi: 10.1016/j.crmeth.2024.100695.

19. ANTsX. Advanced Normalization Tools (ANTs), https://github.com/ANTsX/ANTs.

20. Tustison NJ, Cook PA, Klein A, Song G, Das SR, Duda JT, et al.. Large-scale evaluation of ANTs and FreeSurfer cortical thickness measurements. *Neuroimage*. 2014; doi: 10.1016/j.neuroimage.2014.05.044.

21. Marcus DS, Wang TH, Parker J, Csernansky JG, Morris JC, Buckner RL. Open Access Series of Imaging Studies (OASIS): Cross-sectional MRI Data in Young, Middle Aged, Nondemented, and Demented Older Adults. *Journal of Cognitive Neuroscience*. 2007; doi: 10.1162/jocn.2007.19.9.1498.

22. Evans AC, Janke AL, Collins DL, Baillet S. Brain templates and atlases. *NeuroImage*. 2012; doi: 10.1016/j.neuroimage.2012.01.024.

23. Li Y, Sadee C, Carrillo-Perez F, Selby H, Thieme A, Gevaert O. A 3D Lung Lesion Variational Auto-Encoder. Rochester, NY;

24. Ronneberger O, Fischer P, Brox T. U-Net: Convolutional Networks for Biomedical Image Segmentation. Medical Image Computing and Computer-Assisted Intervention – MICCAI 2015. MICCAI 2015. Lecture Notes in Computer Science, vol 9351. Springer, Cham. https://doi.org/10.1007/978-3-319-24574-4_28

25. Çiçek Ö, Abdulkadir A, Lienkamp SS, Brox T, Ronneberger O. 3D U-Net: Learning Dense Volumetric Segmentation from Sparse Annotation. Medical Image Computing and Computer-Assisted Intervention – MICCAI 2016. MICCAI 2016. Lecture Notes in Computer Science, vol 9901. Springer, Cham. https://doi.org/10.1007/978-3-319-46723-8_49

26. Hjelm RD, Plis SM, Calhoun VC. Variational Autoencoders for Feature Detection of Magnetic Resonance Imaging Data. arXiv; 2016; doi: 10.48550/arXiv.1603.06624

27. Tsutsumi M, Saito N, Koyabu D, Furusawa C. A deep learning approach for morphological feature extraction based on variational auto-encoder: an application to mandible shape. *npj Syst Biol Appl*. Nature Publishing Group; 2023; doi: 10.1038/s41540-023-00293-6.

28. Wang Z, Simoncelli EP, Bovik AC. Multiscale structural similarity for image quality assessment. *The Thrity-Seventh Asilomar Conference on Signals, Systems & Computers, 2003*. doi: 10.1109/ACSSC.2003.1292216

29. Asperti A, Trentin M. Balancing Reconstruction Error and Kullback-Leibler Divergence in Variational Autoencoders. *IEEE Access*. 2020; doi: 10.1109/ACCESS.2020.3034828.

30. Prokhorov V, Shareghi E, Li Y, Pilehvar MT, Collier N. On the Importance of the Kullback-Leibler Divergence Term in Variational Autoencoders for Text Generation. arXiv; 2019; doi: 10.48550/arXiv.1909.13668

31. van Griethuysen JJM, Fedorov A, Parmar C, Hosny A, Aucoin N, Narayan V, et al.. Computational Radiomics System to Decode the Radiographic Phenotype. *Cancer Res*. 2017; doi: 10.1158/0008-5472.CAN-17-0339.

32. The UK Biobank: https://www.ukbiobank.ac.uk/.

33. Sudlow C, Gallacher J, Allen N, Beral V, Burton P, Danesh J, et al.. UK biobank: an open access resource for identifying the causes of a wide range of complex diseases of middle and old age. *PLoS Med*. 2015; doi: 10.1371/journal.pmed.1001779.

34. The Center for Biomedical Research Excellence (COBRE): https://fcon_1000.projects.nitrc.org/indi/retro/cobre.html.

35. NITRC: MCIC- schizophrenic and matched control data: https://www.nitrc.org/projects/mcic/.

36. Gollub RL, Shoemaker JM, King MD, White T, Ehrlich S, Sponheim SR, et al.. The MCIC collection: a shared repository of multi-modal, multi-site brain image data from a clinical investigation of schizophrenia. *Neuroinformatics*. 2013; doi: 10.1007/s12021-013-9184-3.

37. Aine CJ, Bockholt HJ, Bustillo JR, Cañive JM, Caprihan A, Gasparovic C, et al.. Multimodal Neuroimaging in Schizophrenia: Description and Dissemination. *Neuroinformatics*. 2017; doi: 10.1007/s12021-017-9338-9.

38. The Cancer Imaging Archive (TCIA): UCSF-PDGM, https://www.cancerimagingarchive.net/collection/ucsf-pdgm/.

39. Clark K, Vendt B, Smith K, Freymann J, Kirby J, Koppel P, et al.. The Cancer Imaging Archive (TCIA): Maintaining and Operating a Public Information Repository. *J Digit Imaging*. 2013; doi: 10.1007/s10278-013-9622-7.

40. Calabrese E, Villanueva-Meyer JE, Rudie JD, Rauschecker AM, Baid U, Bakas S, et al.. The University of California San Francisco Preoperative Diffuse Glioma MRI Dataset. *Radiology: Artificial Intelligence*. Radiological Society of North America; 2022; doi: 10.1148/ryai.220058.

41. The Cancer Imaging Archive (TCIA): UPENN-GBM, https://www.cancerimagingarchive.net/collection/upenn-gbm/.

42. Bakas S, Sako C, Akbari H, Bilello M, Sotiras A, Shukla G, et al.. The University of Pennsylvania glioblastoma (UPenn-GBM) cohort: advanced MRI, clinical, genomics, & radiomics. *Sci Data*. Nature Publishing Group; 2022; doi: 10.1038/s41597-022-01560-7.

43. The Cancer Imaging Archive (TCIA): TCGA-LGG, portal.gdc.cancer.gov/projects/TCGA-LGG.

44. The Cancer Imaging Archive (TCIA): TCGA-GBM, portal.gdc.cancer.gov/projects/TCGA-GBM.

45. McLendon R, Friedman A, Bigner D, Van Meir EG, Brat DJ, M. Mastrogianakis G, et al.. Comprehensive genomic characterization defines human glioblastoma genes and core pathways. *Nature*. Nature Publishing Group; 2008; doi: 10.1038/nature07385.

46. Alzheimer's Disease Neuroimaging Initiative (ADNI): https://adni.loni.usc.edu/data-samples/adni-data/neuroimaging/mri/mri-image-data-sets/.

47. Barba T. DUNE: a versatile neuroimaging encoder captures brain complexity across three major diseases: cancer, dementia and schizophrenia. *FigShare*. 2025; doi: 10.6084/m9.figshare.29538347.v1.

48. Barba T, Bagley BA, Steyaert S, Carrillo-Perez F, Sadée CY, Iv M, et al.. Supporting data for "DUNE: a versatile neuroimaging encoder captures brain complexity across three major diseases: cancer, dementia and schizophrenia." *GigaScience Database*. 2025; https://doi.org/10.5524/102757

49. Chang K, Bai HX, Zhou H, Su C, Bi WL, Agbodza E, et al.. Residual Convolutional Neural Network for the Determination of IDH Status in Low- and High-Grade Gliomas from MR Imaging. *Clin Cancer Res*. 2018; doi: 10.1158/1078-0432.CCR-17-2236.

50. Li Z, Wang Y, Yu J, Guo Y, Cao W. Deep Learning based Radiomics (DLR) and its usage in noninvasive IDH1 prediction for low grade glioma. *Sci Rep*. Nature Publishing Group; 2017; doi: 10.1038/s41598-017-05848-2.

51. Nalawade S, Murugesan G, Vejdani-Jahromi M, Fisicaro RA, Yogananda CGB, Wagner B, et al.. Classification of Brain Tumor IDH Status using MRI and Deep Learning. bioRxiv; 2019; doi: https://doi.org/10.1101/757344

52. Azad R, Aghdam EK, Rauland A, Jia Y, Avval AH, Bozorgpour A, et al.. Medical Image Segmentation Review: The success of U-Net. IEEE Transactions on Pattern Analysis and Machine Intelligence, vol. 46, no. 12, pp. 10076-10095, Dec. 2024, doi: 10.1109/TPAMI.2024.3435571.

53. Ma R, Xie R, Wang Y, Meng J, Wei Y, Xi W, et al.. Autism Spectrum Disorder Classification in Children based on Structural MRI Features Extracted using Contrastive Variational Autoencoder. arXiv; 2025; https://doi.org/10.48550/arXiv.2307.00976

54. Lambert B, Louis M, Doyle S, Forbes F, Dojat M, Tucholka A. Leveraging 3D Information in Unsupervised Brain MRI Segmentation. 2021 IEEE 18th International Symposium on Biomedical Imaging (ISBI), Nice, France, 2021, pp. 187-190, doi: 10.1109/ISBI48211.2021.9433894.

55. Volokitin A, Erdil E, Karani N, Tezcan KC, Chen X, Van Gool L, et al.. Modelling the Distribution of 3D Brain MRI using a 2D Slice VAE. Medical Image Computing and Computer Assisted Intervention – MICCAI 2020. MICCAI 2020. Lecture Notes in Computer Science, vol 12267. Springer, Cham. https://doi.org/10.1007/978-3-030-59728-3_64

56. Hoang GM, Kim U-H, Kim JG. Vision transformers for the prediction of mild cognitive impairment to Alzheimer's disease progression using mid-sagittal sMRI. *Front Aging Neurosci*. 2023; doi: 10.3389/fnagi.2023.1102869.

57. Asiri AA, Shaf A, Ali T, Shakeel U, Irfan M, Mehdar KM, et al.. Exploring the Power of Deep Learning: Fine-Tuned Vision Transformer for Accurate and Efficient Brain Tumor Detection in MRI Scans. *Diagnostics (Basel)*. 2023; doi: 10.3390/diagnostics13122094.

58. Vaswani A, Shazeer N, Parmar N, Uszkoreit J, Jones L, Gomez AN, et al.. Attention Is All You Need. arXiv; 2023; https://doi.org/10.48550/arXiv.1706.03762

59. Dosovitskiy A, Beyer L, Kolesnikov A, Weissenborn D, Zhai X, Unterthiner T, et al.. An Image is Worth 16x16 Words: Transformers for Image Recognition at Scale. arXiv; 2021; https://doi.org/10.48550/arXiv.2010.11929

60. Raghu M, Unterthiner T, Kornblith S, Zhang C, Dosovitskiy A. Do Vision Transformers See Like Convolutional Neural Networks? arXiv; 2022; https://doi.org/10.48550/arXiv.2108.08810

61. Teixeira B, Singh V, Chen T, Ma K, Tamersoy B, Wu Y, et al.. Generating Synthetic X-ray Images of a Person from the Surface Geometry. IEEE/CVF Conference on Computer Vision and Pattern Recognition, Salt Lake City, UT, USA, 2018, pp. 9059-9067, doi: 10.1109/CVPR.2018.00944.

62. Mahmood F, Borders D, Chen RJ, Mckay GN, Salimian KJ, Baras A, et al.. Deep Adversarial Training for Multi-Organ Nuclei Segmentation in Histopathology Images. *IEEE Transactions on Medical Imaging*. 2020; doi: 10.1109/TMI.2019.2927182.

63. Chen RJ, Lu MY, Chen TY, Williamson DFK, Mahmood F. Synthetic data in machine learning for medicine and healthcare. *Nat Biomed Eng*. Nature Publishing Group; 2021; doi: 10.1038/s41551-021-00751-8.

64. Gao C, Killeen BD, Hu Y, Grupp RB, Taylor RH, Armand M, et al.. Synthetic data accelerates the development of generalizable learning-based algorithms for X-ray image analysis. *Nat Mach Intell*. Nature Publishing Group; 2023; doi: 10.1038/s42256-023-00629-1.

65. Kobayashi K, Miyake M, Takahashi M, Hamamoto R. Observing deep radiomics for the classification of glioma grades. *Sci Rep*. Nature Publishing Group; 2021; doi: 10.1038/s41598-021-90555-2.

66. Yamaguchi H, Hashimoto Y, Sugihara G, Miyata J, Murai T, Takahashi H, et al.. Three-Dimensional Convolutional Autoencoder Extracts Features of Structural Brain Images With a “Diagnostic Label-Free” Approach: Application to Schizophrenia Datasets. *Frontiers in Neuroscience*. 152021;

67. Lambin P, Rios-Velazquez E, Leijenaar R, Carvalho S, van Stiphout RGPM, Granton P, et al.. Radiomics: extracting more information from medical images using advanced feature analysis. *Eur J Cancer*. 2012; doi: 10.1016/j.ejca.2011.11.036.

68. Huang Y, Liu Z, He L, Chen X, Pan D, Ma Z, et al.. Radiomics Signature: A Potential Biomarker for the Prediction of Disease-Free Survival in Early-Stage (I or II) Non-Small Cell Lung Cancer. *Radiology*. 2016; doi: 10.1148/radiol.2016152234.

69. Li Y, Ammari S, Lawrance L, Quillent A, Assi T, Lassau N, et al.. Radiomics-Based Method for Predicting the Glioma Subtype as Defined by Tumor Grade, IDH Mutation, and 1p/19q Codeletion. *Cancers*. Multidisciplinary Digital Publishing Institute; 2022; doi: 10.3390/cancers14071778.

70. Khorrami M, Viswanathan VS, Reddy P, Braman N, Kunte S, Gupta A, et al.. Radiomic predicts early response to CDK4/6 inhibitors in hormone receptor positive metastatic breast cancer. *NPJ Breast Cancer*. 2023; doi: 10.1038/s41523-023-00574-7.

71. Steyaert S, Qiu YL, Zheng Y, Mukherjee P, Vogel H, Gevaert O. Multimodal deep learning to predict prognosis in adult and pediatric brain tumors. *Commun Med (Lond)*. 2023; doi: 10.1038/s43856-023-00276-y.

ACKNOWLEDGEMENTS

This research has been conducted using the UK Biobank Resource under Application Number 51998.

The results shown here are in whole or part based upon data generated by the TCGA Research Network: http://cancergenome.nih.gov

Data was downloaded from the COllaborative Informatics and Neuroimaging Suite Data Exchange tool (COINS; http://coins.mrn.org/dx) and data collection was performed at the Mind Research Network (www.mrn.org) and funded by a Center of Biomedical Research Excellence (COBRE) grant 5P20RR021938/P20GM103472 from the NIH to Dr. Vince Calhoun. The MCIC project was supported by the Department of Energy under Award Number DE-FG02-08ER64581. MCIC is the result of efforts of co-investigators from University of Iowa, University of Minnesota, University of New Mexico, Massachusetts General Hospital.

Data collection and sharing for this project were funded by the Alzheimer’s Disease Neuroimaging Initiative (ADNI) (National Institutes of Health Grant U01 AG024904) and DOD ADNI (Department of Defense award number W81XWH-12-2-0012). ADNI is funded by the National Institute on Aging, the National Institute of Biomedical Imaging and Bioengineering, and through generous contributions from the following: AbbVie, Alzheimer’s Association; Alzheimer’s Drug Discovery Foundation; Araclon Biotech; BioClinica, Inc.; Biogen; Bristol-Myers Squibb Company; CereSpir, Inc.; Cogstate; Eisai Inc.; Elan Pharmaceuticals, Inc.; Eli Lilly and Company; EuroImmun; F. Hoffmann-La Roche Ltd. and its affiliated company Genentech, Inc.; Fujirebio; GE Healthcare; IXICO Ltd.; Janssen Alzheimer Immunotherapy Research & Development, LLC.; Johnson & Johnson Pharmaceutical Research & Development LLC.; Lumosity; Lundbeck; Merck & Co., Inc.; Meso Scale Diagnostics, LLC.; NeuroRx Research; Neurotrack Technologies; Novartis Pharmaceuticals Corporation; Pfizer Inc.; Piramal Imaging; Servier; Takeda Pharmaceutical Company; and Transition Therapeutics. The Canadian Institutes of Health Research is providing funds to support ADNI clinical sites in Canada. Private sector contributions are facilitated by the Foundation for the National Institutes of Health (http://www.fnih.org). The grantee organization is the Northern California Institute for Research and Education, and the study is coordinated by the Alzheimer’s Therapeutic Research Institute at the University of Southern California. ADNI data are disseminated by the Laboratory for Neuroimaging at the University of Southern California. Data used in the preparation of this article were obtained from the Alzheimer’s Disease Neuroimaging Initiative (ADNI) database (http://adni.loni.usc.edu). As such, the investigators within the ADNI contributed to the design and implementation of ADNI and/or provided data but did not participate in the analysis or writing of this report. A complete listing of ADNI investigators can be found at: https://adni.loni.usc.edu/wp-content/uploads/how_to_apply/ADNI_Acknowledgement_List.pdf.

Research reported here was further supported by the National Cancer Institute (NCI) under awards: R01 CA260271. The content is solely the responsibility of the authors and does not necessarily represent the official views of the National Institutes of Health.

AUTHOR CONTRIBUTIONS

Conceptualization: OG, TB
Methodology: OG, TB, SS, BB, MI
Investigation: TB, MI
Visualization: TB
Funding acquisition: OG
Project administration: OG
Supervision: OG
Writing – original draft: TB
Writing – review & editing: OG, FP, CS, SS

COMPETING INTERESTS

Authors declare that they have no competing interests.

Figure 1 Click here to access/download;Figure;Fig1.pdf

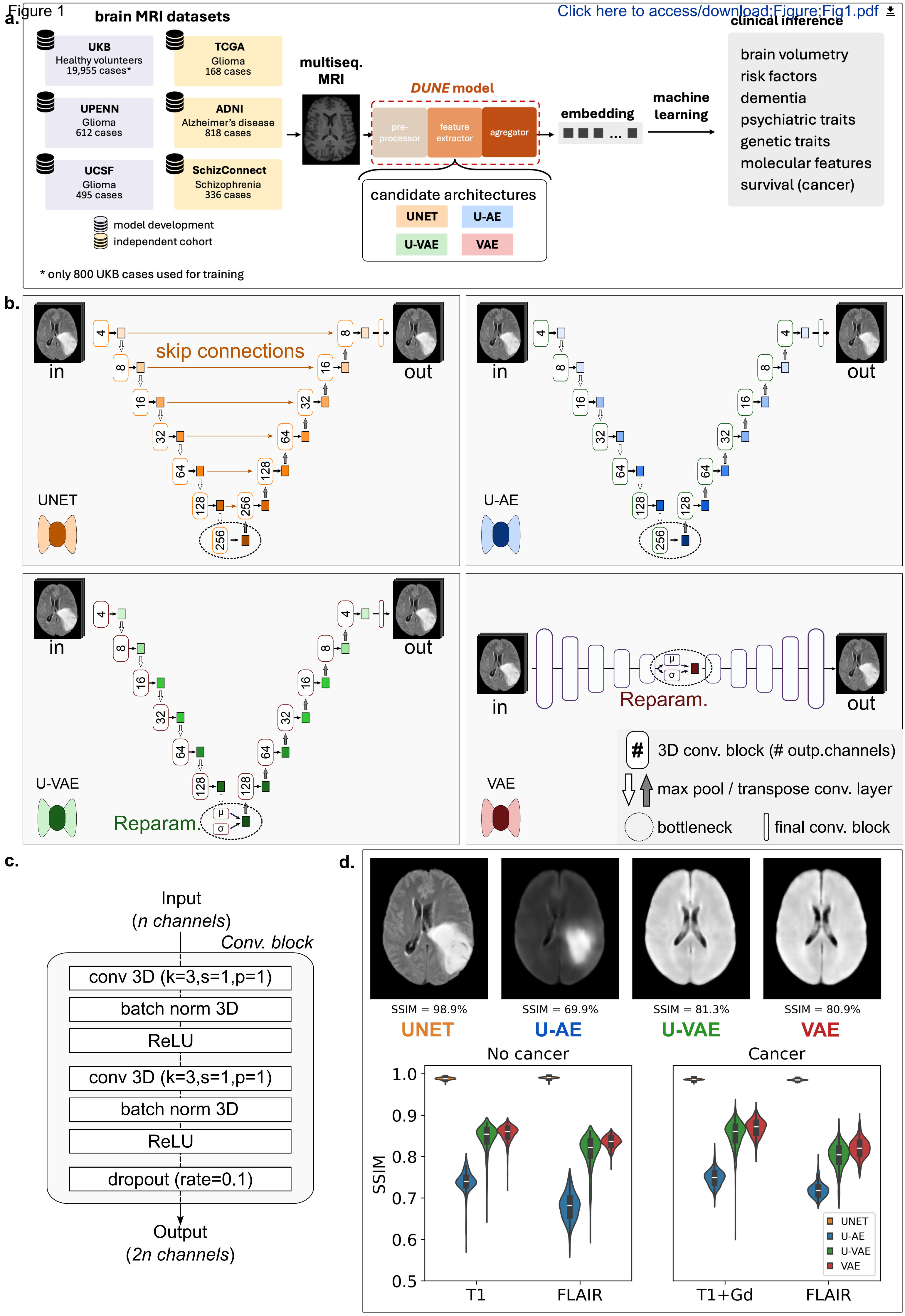

Figure 2

Click here to access/download;Figure;Fig2.pdf

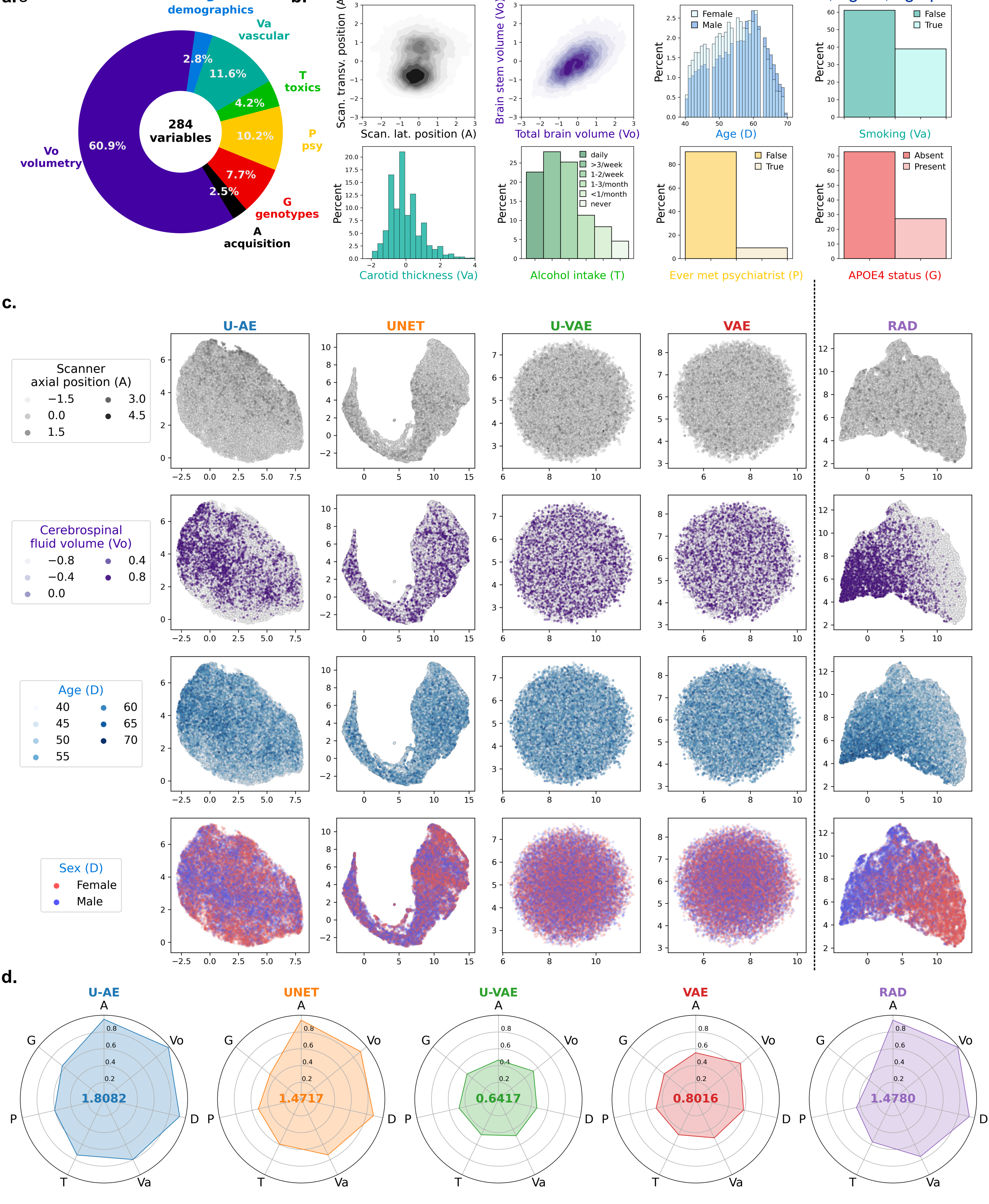

Figure 3

Click here to access/download;Figure;Fig3.pdf

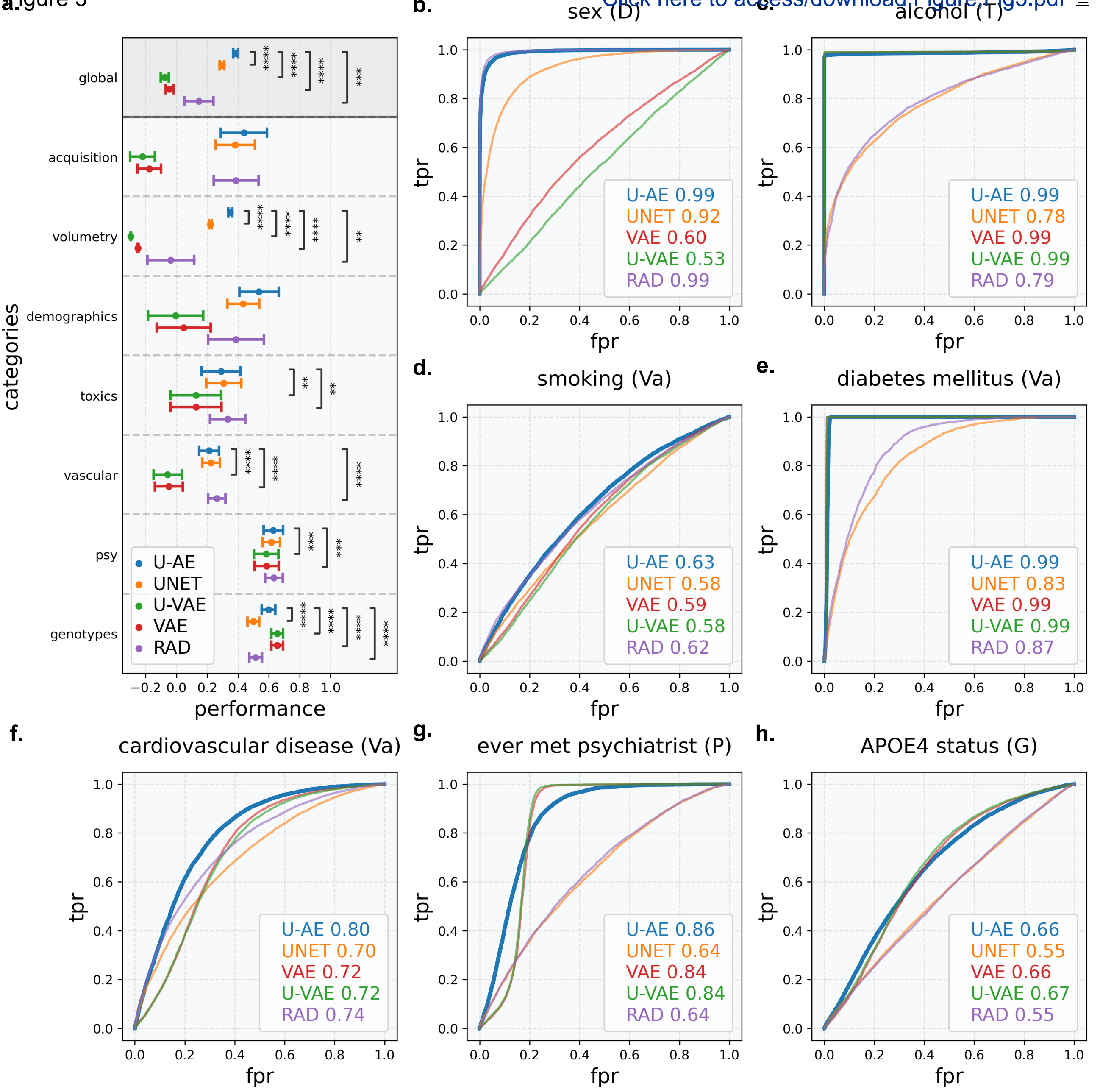

Figure 4

Click here to access/download;Figure;Fig4.pdf

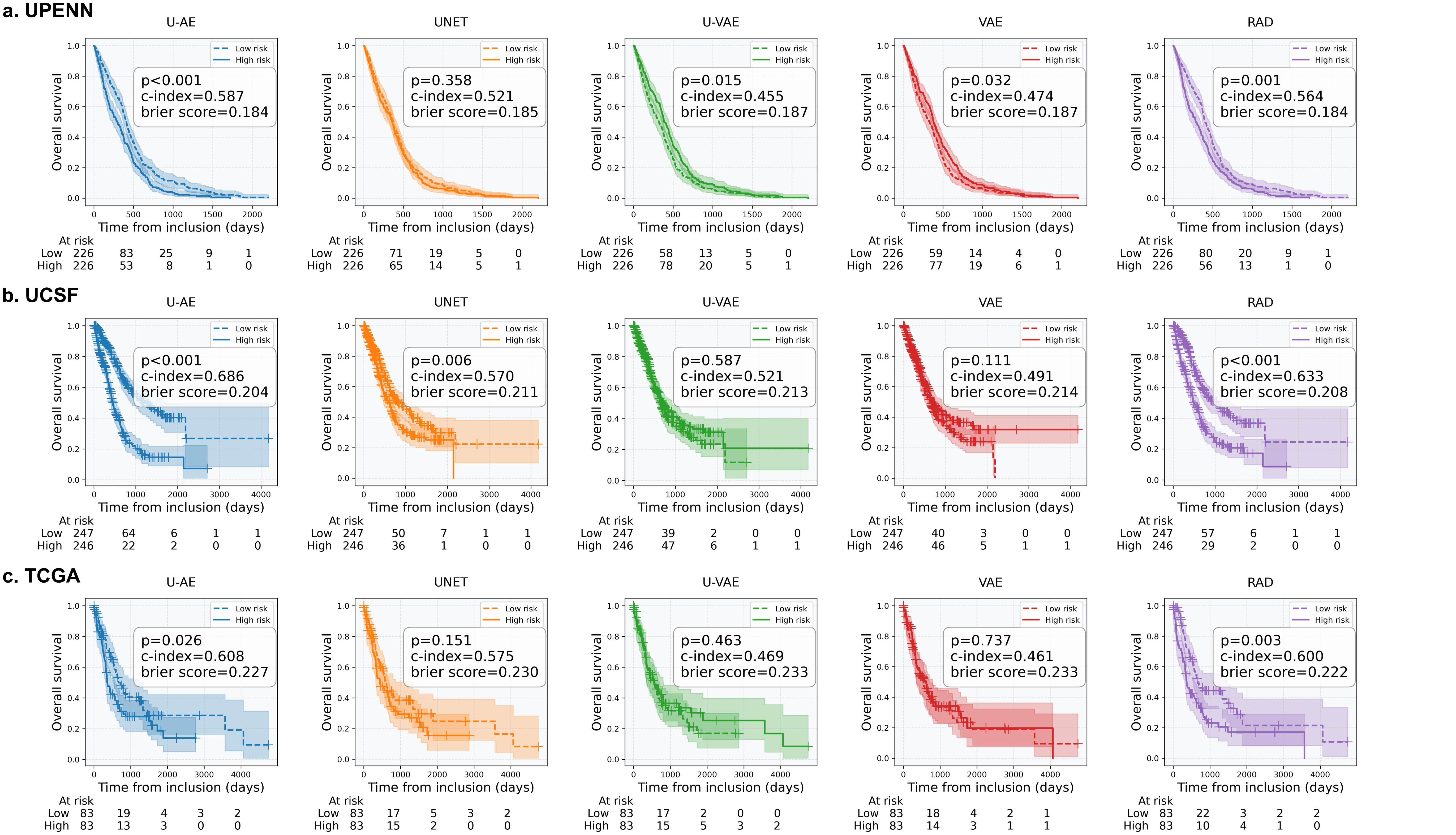

Figure 5

Click here to access/download;Figure;Fig5.pdf

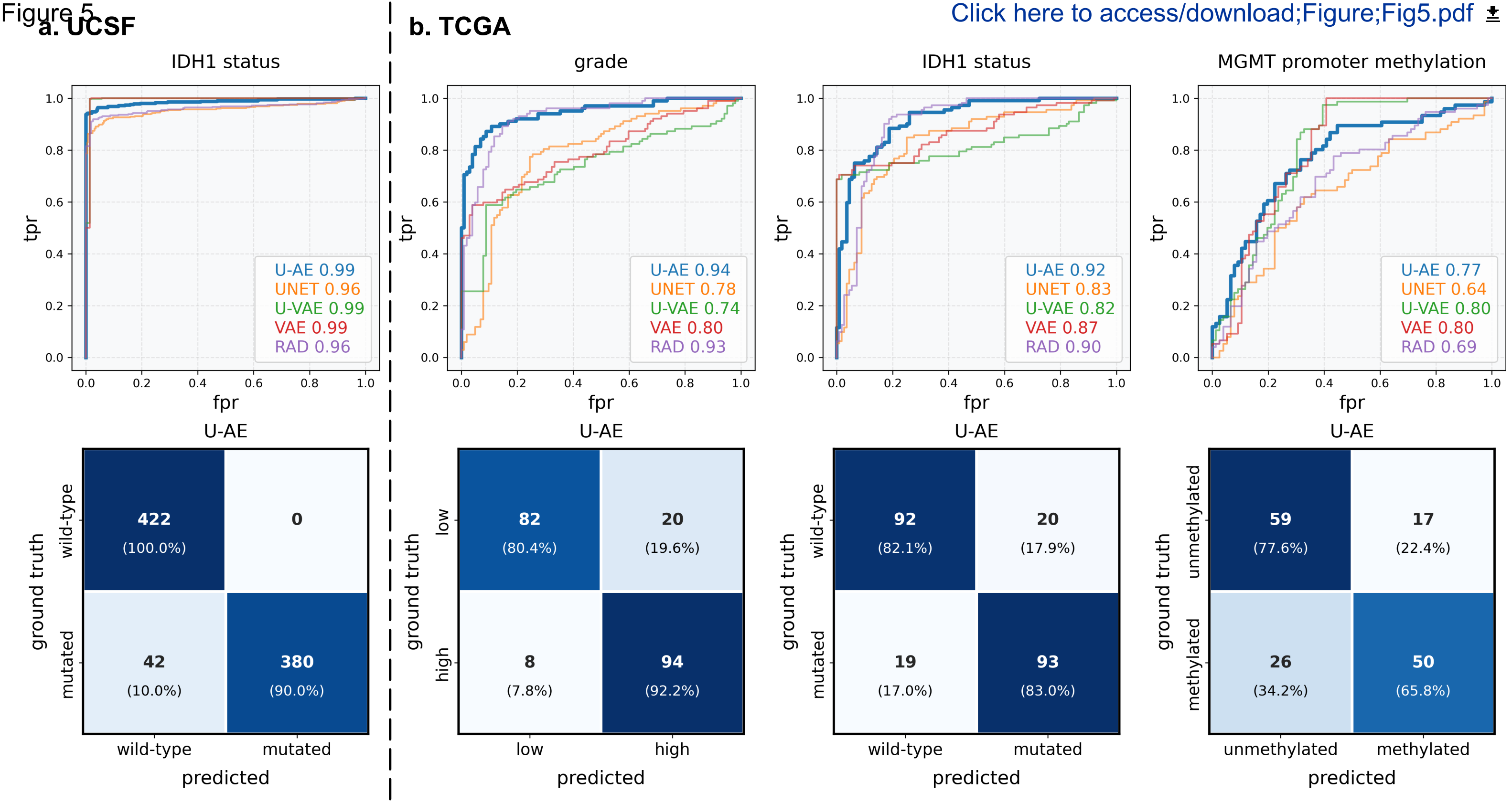

Figure 6

Click here to access/download;Figure;Fig6.pdf

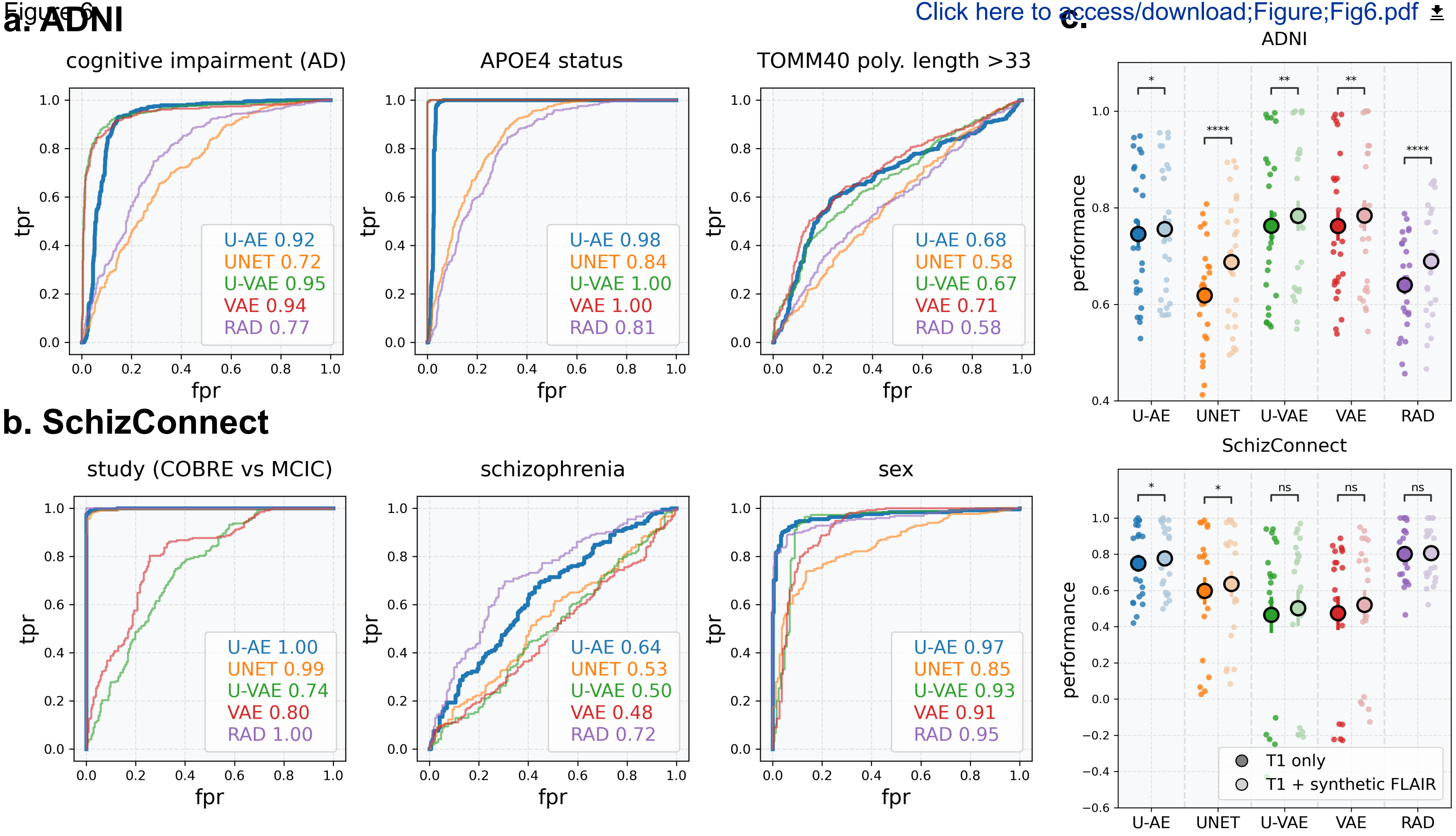

Supplemental figure 1

Click here to access/download
Supplementary Material
Supplemental Fig 1.pdf

Supplemental figure 2

Click here to access/download
Supplementary Material
Supplemental Fig 2.pdf

Supplemental figure 3

Click here to access/download
Supplementary Material
Supplemental Fig 3.pdf

Response

Click here to access/download
Supplementary Material
Gigascience Reponse.docx

Cover letter Click here to access/download;Personal Cover;CoverLetter.pdf

Dr Thomas BARBA
Assistant Professor of Internal Medicine
Department of Internal Medicine
Edouard Herriot Hospital, Lyon University Hospitals
5 place d'Arsonval, 69003 Lyon
Phone: +33472117565

July 18th 2025

Dear Editor-in-Chief,

We are pleased to submit the revised manuscript entitled « *DUNE: a versatile neuroimaging encoder captures brain complexity across three major diseases: cancer, dementia and schizophrenia* » for publication in *Gigascience*. We deeply appreciate the constructive feedback provided by the reviewers and have addressed all comments in this revision.

This work introduces DUNE, a **comprehensive neuroimaging workflow** that transforms multisequence raw brain MRI scans into standardized compact patient-level embeddings through integrated preprocessing and deep feature extraction. The revised manuscript demonstrates the workflow's ability to generalize across multiple neurological conditions while maintaining robust performance on external validation cohorts.

Key improvements in this revision include:

- **Enhanced methodological transparency**: we have completely restructured the manuscript following the standard IMRAD format, moving the methods section before results to improve readability and comprehension.
- **Comprehensive architectural comparison**: following reviewer suggestions, we conducted additional experiments comparing our approach with end-to-end CNN models and enhanced loss functions, further validating our architectural choices and demonstrating the counter-intuitive finding that reconstruction quality does not predict embedding utility for clinical applications.
- **Expanded technical details**: we have significantly enhanced the synthetic data generation methodology description and provided detailed explanations of all preprocessing steps, training algorithms, and evaluation frameworks.
- **Improved reproducibility**: All code necessary to execute the complete DUNE pipeline is now publicly available on GitHub at https://github.com/gevaertlab/DUNE. Additionally, data and code required to reproduce all figures have been deposited on Figshare (https://figshare.com/s/d0591f66088691014bf0) ensuring full computational reproducibility.
- **Corrected numerical inconsistencies**: We have addressed all statistical reporting errors identified by the reproducibility reviewer and standardized our terminology throughout the manuscript.

The clinical relevance and impact of our work remain compelling. DUNE successfully captures diverse clinical parameters across healthy volunteers and patients with gliomas, Alzheimer's disease, and schizophrenia, achieving robust performance comparable to or exceeding task-specific deep learning models. Notably, the embeddings capture subtle molecular features such as IDH1 mutation status (AUROC=0.92) and genetic traits like APOE4 allele status, while maintaining excellent generalization across independent external cohorts.

Our findings advance the field of medical imaging by demonstrating that unsupervised encoder-decoder networks can extract diagnostically meaningful features across multiple disease domains. The workflow's

ability to handle incomplete imaging datasets through synthetic sequence generation further enhances its clinical applicability, particularly relevant given current pressures to optimize MRI protocols.

We believe this revised manuscript significantly strengthens the original contribution and addresses all reviewer concerns while maintaining the innovative aspects that make DUNE a valuable addition to the neuroimaging toolkit. The comprehensive workflow we present offers immediate utility for researchers and clinicians seeking to leverage brain MRI data for diverse clinical applications.

We confirm that this work is original, has not been published elsewhere, and is not under consideration by any other journal.

Thank you for the opportunity to revise and improve our manuscript. We are confident that this enhanced version makes a substantial contribution to the field and aligns perfectly with *Gigascience*'s commitment to advancing computational biology and data-driven medical research.

Sincerely,

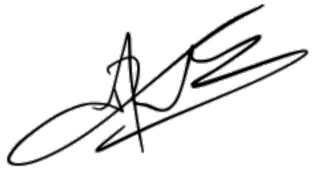

Dr Thomas Barba, MD, PhD
Assistant Professor of Internal Medicine
Department of Internal Medicine Lyon University Hospital, France
thomas.barba@chu-lyon.fr
