# Supplementary material for: DUNE: a versatile neuroimaging encoder captures brain complexity across 3 major diseases: cancer, dementia, and schizophrenia
Source: Gigascience. 2025 Oct 15;14:giaf116. doi: 10.1093/gigascience/giaf116 (PMC12527335; doi:10.1093/gigascience/giaf116)
Supplement: giaf116_Authors_Response_To_Reviewer_Comments_Original_Submission [file giaf116_authors_response_to_reviewer_comments_original_submission.pdf]

Author's Response To Reviewer Comments

See attached document and here:

Reviewer reports

The authors’ responses are marked in blue.
Changes in the manuscript are marked in orange.

Reviewer #1: Reproducibility report for: DUNE: a versatile neuroimaging encoder captures brain complexity across three major diseases: cancer, dementia and schizophrenia
Journal: Gigascience
ID number/DOI: GIGA-D-25-00076
Reviewer(s): Laura Caquelin, Department of Clinical Neuroscience, Karolinska Institutet, Sweden [Wrote the report and reproduced the results]
Gustav Nilsonne, Department of Clinical Neuroscience, Karolinska Institutet, Sweden [Reviewed the final report]
----

1. Summary of the Study
The manuscript introduces DUNE , a neuroimaging-oriented (UNet-based) encoder designed to extract deep features from multisequence brain MRIs, enabling their analysis with machine learning models. It was trained on scans of healthy and glioma-affected brains to generate low-dimensional representations. These embeddings were then used to train models predicting various clinical variables and tested on independent cohorts to assess generalization.

----

2. Scope of reproducibility

According to our assessment the primary objective is: "to test prediction on MRI embeddings accurately predict clinical phenotypes of healthy individuals." (page 12)

- Outcome: the predictive performance of machine learning models trained on different MRI-derived embeddings for clinical phenotype prediction.
- Analysis method outcome: "The predictions from all validation folds were concatenated to generate ROC curves and compute performance metrics: weighted F1-scores for categorical variables, R^2 scores for quantitative variables, and C-indexes and Integrated Brier Scores for survival variables. The predictive performance of models using different embeddings was compared using Wilcoxon signed-rank tests with Bonferroni correction for multiple comparisons." (page 26)
- Main result: "Overall, the predictions were best with U-AE embeddings (0.384±0.27%), which significantly outperformed radiomics (0.144±1.58, $p<0.001$, Fig 3a). The embeddings encoded by other models (UNET, U-VAE, VAE) resulted in weaker predictions. The performances greatly varied between the different subgroups of clinical variables but showed small variation across variables within each category (Fig 3a), which tended to be highly intercorrelated (Supplemental Figure S2)." (page 12)

----

3. Availability of Materials
a. Data
- Data availability: Not open, not yet publicly available, shared on the FTP server
- Data completeness: Complete = all data necessary to reproduce main results are available
- Access Method: Private journal dropbox
- Repository: -

- Data quality: The data files have been shared and appear sufficient for running the analyses. However, no metadata is provided to describe the content, structure, or origin of the files which limits interpretability and reusability.
b. Code
- Code availability: The code for the DUNE tools is publicly available, while the code required to reproduce the main results of the manuscript is private.
- Programming Language(s): Python
- Repository link: https://github.com/gevaertlab/BrainMR_AE_datafusion/ The code to reproduce the results is hosted on a private FTP server.
- License: Apache 2.0
- Repository status: Public for DUNE tools, Private for result reproduction code.
- Documentation: No documentation shared

----

4. Computational environment of reproduction analysis

- Operating system for reproduction: MacOS 14.7.4
- Programming Language(s): Python
- Code implementation approach: Using shared code
- Version environment for reproduction: Python 3.13.3

----

5. Results

5.1 Original study results
- Results 1: Results presented in figure 3A in the manuscript.

5.2 Steps for reproduction

-> Identify the numerical values of inferential statistical results in relation to the conclusions of Figure 3A.
- Issue 1: Numerical p-values are not provided, only indicated with asterisks in Figure 3A.
-- Resolved: The figure presents significance markers but does not report the exact p-values used for model comparisons, same for the means and standard errors across variables.
After request, the authors provided the values in files shared on private FTP servers.
- Issue 2: The raw data necessary to reproduce Figure 3A are not available.
-- Resolved: The dataset containing prediction scores (R^2 or F1-scores) across variables in each category for each model is not shared in the GitHub repository.
After request, the authors provided the raw data, and the script shared on the private FTP servers.
-> Run the Python script provided to reproduce the results of Figure 3A.
- Issue 3: Missing package installations required to run the code.
-- Resolved: There was no README file specifying the required dependencies. As a result, I had to manually identify and install the necessary packages. Some dependencies were sub-modules, so I had to search for the full package to install. For instance, sklearn.metrics is part of the larger scikit-learn package, sksurv.metrics part of scikit-survival package, and statannotations.Annotator part of statannotations package. Also statsmodels was also needed for certain statistical functions.
- Issue 4: Fixed paths that don't work on other computers.
-- Resolved: In the original code, there were fixed file paths (/Users/tom/drive/...) that only worked on one specific computer. This caused problems when running the code on other computers. To fix this, I changed the code to use relative paths, which are based on where the script is located. This way, the code can be run on any computer without needing to change the paths each time.

------------------ Start of script ------------------
```
# Configuration
# [Modification by LC]BEGIN------------
#os.chdir("/Users/tom/drive/2-postdoc/1-projects/brain_autoencoder/MS")
```

```
os.getcwd()
OUTPUT_DIR = "./GigaScience_review"
# [Modification by LC]END------------
FONTSIZE = 16
UKB_FONTSIZE = 16
UKB_FONTWEIGHT = 'regular'
SHADOW = False

# Paths
# [Modification by LC]BEGIN------------
#DATA_PATHS = {
#"METADATA_FILE": "/Users/tom/drive/2-postdoc/1-
projects/brain_autoencoder/MS/GigaScience/variable_metadata.csv",
#"PREDICTIONS_DIR": "/Users/tom/drive/2-postdoc/1-projects/brain_autoencoder/MS/GigaScience",
#}
SCRIPT_DIR = os.path.dirname(os.path.abspath(__file__))

DATA_PATHS = {
"METADATA_FILE": os.path.join(SCRIPT_DIR, "variable_metadata.csv"),
"PREDICTIONS_DIR": os.path.join(SCRIPT_DIR),
}
# [Modification by LC]END------------
```
------------------- End of script -------------------

5.3 Statistical comparison Original vs Reproduced results
- Results: From the python script shared, Figure 3, the category performance_stats and the statistical_comparison_pvalues were obtained.

Comparison between the category_performance_stats and the statistical_comparison_pvalues files from the original results and the reproduced ones was conducted using R (script attached to the review).

- Comments: No particular comments.
- Errors detected: Corresponding to the sentence "Overall, the predictions were best with U-AE embeddings (0.384±0.27%), which significantly outperformed radiomics (0.144±1.58, p<0.001, Fig 3a)." (page 13):
-- The reported value for radiomics (0.144) should be rounded to 0.145 based on the given data (0.144650928571429). No rounding error is present for the U-AE value, which is correctly reported as 0.384 based on the given data (0.384448357142857).
-- There seems to be an inconsistency in the reported standard errors for both the U-AE and radiomics models. The paper reports a standard error of "0.384 ± 0.27%" for U-AE and "0.144 ± 1.58%" for Radiomics. Based on the provided data, the actual standard errors for these models are much higher: 1.63% (0.0162718529517984) for U-AE and 9.47% (0.0947012592300183) for radiomics.
- Statistical Consistency:
-- Graphically, the reproduced Figure 3A appears to correspond to the original one.
-- For the data in 0-category_performance_stats.csv, no differences were observed between the original results and the reproduced values.
-- For the data in 0-statistical_comparison_pvalues.csv, only one minor numerical difference was found: the p-value for category "psy", model "U-AE/RAD", differed slightly between the original (8.983406e-01) and the reproduced (8.967698e-01) results. All other values matched exactly.

We thank the reviewer for carefully identifying these numerical inconsistencies in our reported values. We have corrected these errors in the revised manuscript.
Changes made:
Results, page 9 line 40: Clinical prediction performance varied across embedding types (Fig 3a). U-AE embeddings achieved prediction scores of 0.384±1.63%, compared to radiomics (0.145±9.7%, p<0.001). UNET, U-VAE, and VAE embeddings achieved lower prediction scores across clinical variables.

6. Conclusion
- Summary of the computational reproducibility review
The computational reproducibility review confirmed that the results in the original study related to the main objective defined according to our assessment were reproducible using the data and code provided by the authors. The graphical representation in Figure 3A was successfully reproduced, and no differences were found between the original and reproduced values for the category_performance_stats dataset.
However, a minor numerical difference was detected in the statistical_comparison_pvalues dataset for the "psy" category, U-AE/RAD model, where the p-value slightly differed between the original (8.983406e-01) and the reproduced (8.967698e-01) results.
In addition, there are inconsistencies with rounding and discrepancies in the reported standard errors, which need to be resolved.
In summary, the results were almost fully reproducible using the original code, with only a minor discrepancy.

- Recommendations for authors
We were able to reproduce the study because we had access to the data and code provided on the private journal FTP server. To ensure that the study remains fully reproducible in the future, the following recommendations are strongly advised:

 Make the code and data to reproduce all the paper openly available to the public to allow anyone to reproduce the figures and analysis of the paper.
 Provide a README file or requirements.txt file to list all dependencies and ensure that the code can be run without issues. This is particularly important when using sub-modules and non-standard libraries. Clear instructions should also be included regarding how to set up the environment and run the analysis, we suggest:
o A brief description of each script or analysis pipeline.
o An indication of which figure, table, or result in the manuscript each script corresponds to
o Clear instructions on how to execute the analyses in the correct order, if applicable.
 Metadata: For the datasets used or generated by the scripts, it would be helpful to include accompanying metadata files that explain:
o The definition of each variable name.
o The origin of each dataset (raw, processed, etc).
o Any preprocessing steps applied before analysis.
 It is also recommended to document any software versions used in the analysis, as this can help avoid issues with version mismatches in future reproductions.
These improvements would enhance the reproducibility and transparency of the work.

We thank the reviewer for these recommendations to enhance the reproducibility and transparency of our work. We have addressed the suggested improvements. All code necessary to execute the complete DUNE pipeline is publicly available on our GitHub repository at https://github.com/gevaertlab/DUNE. All data (save for the UKB data, subject to data agreement) and code (jupyter notebook including code and notice) required to reproduce the figures presented in the manuscript have been deposited on Figshare and are publicly accessible at: https://figshare.com/s/d0591f66088691014bf0.

Reviewer #2: This work introduces DUNE, a versatile deep-learning model designed to extract low-dimensional embeddings from brain MRIs. The model's ability to generalize across multiple diseases, including glioma, Alzheimer's, and schizophrenia, is a significant strength, with broad potential clinical applications. However, there are areas where the manuscript can be improved:

1. While the manuscript compares DUNE to radiomics, a traditional feature extraction method, it would benefit from a comparison with more end-to-end deep learning models or architectures, such as Convolutional Neural Networks (CNNs) or Vision Transformers (ViTs). These models might perform similarly or better for the tasks at hand. This comparison would help establish the superiority or uniqueness of DUNE in terms of its capabilities and performance.
We thank the reviewer for this constructive suggestion regarding comparison with end-to-end deep

learning models such as CNNs and Vision Transformers (ViTs). We acknowledge that such comparisons would provide valuable insights into the relative performance of DUNE compared to other deep learning approaches. However, several practical considerations led us to focus on unsupervised feature extraction rather than end-to-end supervised models.
Vision Transformers (ViTs) present significant challenges when applied to 3D medical imaging data. ViTs typically require extensive datasets for training and lack of inductive biases compared to CNNs. For 3D brain MRI analysis, this data requirement become prohibitive, given the limited availability of medical imaging datasets compared to natural image datasets.
Regarding end-to-end supervised models, a fundamental challenge for our application lies in the heterogeneous nature of our training data. Our model development utilized three distinct datasets (UKB, UPENN, and UCSF) representing fundamentally different populations: healthy volunteers (UKB) and glioma patients (UPENN, UCSF). These cohorts have different clinical characteristics and available labels. Establishing common supervised learning targets across these disparate datasets would be challenging and could introduce significant domain-specific biases.
Despite these challenges, we conducted preliminary experiments to evaluate end-to-end CNN performance using sex prediction as a common variable across datasets. We trained a 3D resnet model on the same training dataset used for DUNE development and tested them on an external dataset (SchizConnect). These models were trained for 150 epochs but exhibited significant overfitting. The results on the external test set showed no discriminative capacity, with performance at chance level.

The unsupervised nature of DUNE offers several advantages over end-to-end supervised approaches: (i) it can leverage heterogeneous datasets without requiring common labels, (ii) it generates versatile embeddings applicable to multiple downstream tasks, (iii) it avoids overfitting to specific prediction tasks, and (iv) it provides better generalization across different clinical applications and populations.
Changes made :
Discussion, Page 15 – line 29: For 3D brain MRI analysis specifically, the computational and data requirements of transformer architectures become prohibitive given the high complexity of volumetric data and the relatively modest size of available medical imaging datasets.

2. The manuscript mentions the use of synthetic data to augment the MRI data, with noted improvements in performance. However, more details on the synthetic data generation process are needed to strengthen the manuscript. A more in-depth explanation of how the synthetic data was created and how it contributes to the model's performance would provide greater clarity.

We thank the reviewer for this suggestion. We agree that the initial description of synthetic data generation in our manuscript was insufficient. We have significantly expanded the Methods section to provide details.
Changes made :
Methods, Page 6 line 45 : To address datasets with limited sequence availability, we implemented a synthetic data enhancement strategy. A dedicated UNet-based model was trained to perform bidirectional synthesis between T1 and FLAIR sequences.
The synthetic data generation model utilized a standard 3D U-Net autoencoder architecture (with skip connections). The model consisted of 6 encoder/decoder blocks with progressive feature expansion (4, 8, 16, 32, 64, and 128 channels), processing single-channel inputs (T1 or FLAIR sequences independently). Each convolutional block comprised two sequential 3D convolution layers (kernel=3, stride=1, padding=1) followed by batch normalization, ReLU activation, and dropout (rate=0.1). Max pooling layers (2x2x2) were used for downsampling in the encoder, while transposed convolution layers (kernel=2, stride=2, padding=0) performed upsampling in the decoder.
The model was trained on the UKB dataset using 39,910 sequences (T1 and FLAIR pairs) with an 80/20 train/validation split, to establish bidirectional correspondence between T1 and FLAIR sequences, enabling the model to learn both T1→FLAIR and FLAIR→T1 synthesis. The training objective utilized SSIM loss to optimize structural similarity between synthethic and corresponding real images, with the ADAM optimizer and learning rate of $5x10^{-5}$.

3. Due to the absence of skip connections, the images reconstructed by the U-AE model inevitably suffer from a loss of details. It would be helpful to explore whether constraints can be imposed on the loss function to encourage the network to preserve image details. For instance, incorporating distance function

constraints (as in Sun et al., 2020, 10.1002/hbm.26098) could be an approach to ensure better retention of details for segmented tissue map.
We thank the reviewer for this insightful suggestion regarding the preservation of image details in our U-AE model architecture. Following the reviewer's recommendation to explore distance function constraints as described in Sun et al. (2020), we conducted additional experiments to investigate this trade-off.
We implemented an improved loss function that combines SSIM reconstruction loss with gradient-based constraints to preserve fine-grained details:
$L_{improved} = \alpha \times L_{SSIM} + \beta \times L_{gradient}$
where $L_{gradient}$ penalizes differences in spatial gradients across all three dimensions, encouraging the network to maintain local image details despite the absence of skip connections.

We trained a modified U-AE model ("constraintUAE", α=1, β=0.1) using this enhanced loss function and evaluated its performance on clinical prediction on three independent cohorts (TCGA, SCHIZO, and ADNI datasets).
• The constraintUAE achieved significantly improved SSIM scores (~80%) compared to the standard U-AE :

However, when evaluating the extracted embeddings for clinical phenotype prediction, the constraintUAE consistently underperformed compared to the standard U-AE across all three external validation datasets:

This experiment further illustrates the trade-off between reconstruction quality and embedding informativeness.

Changes made :
Discussion, Page 14 line 25: Surprisingly, this architecture demonstrated the poorest reconstruction capabilities while producing the most clinically relevant embeddings, highlighting a counter-intuitive finding that reconstruction performance does not predict embedding quality for clinical applications. We further validated this principle through additional experiments with enhanced loss functions incorporating gradient-based detail preservation constraints, which confirmed improved reconstruction quality at the cost of reduced clinical prediction performance (data not shown). This trade-off represents an important consideration in autoencoder architectures, where the removal of skip connections forces all information through the bottleneck layer, creating more informative representations despite reduced pixel-level accuracy.
Reviewer #3: The article addresses the problem of feature extraction from MRI brain images, aiming to support the diagnosis of various brain diseases and pathological conditions. Given the nature of the task, supervised classification networks (such as AlexNet, ResNet, etc.) would typically be considered the primary approach, as these architectures are explicitly designed to extract features enabling optimal discrimination between classes - different pathological brain states.
The authors, however, hypothesize that encoder-decoder neural network architectures (e.g., U-Net and its variants) can effectively extract embedding features relevant to diagnosis. They further propose using unsupervised learning methods, based on the assumption that the reconstructed image should closely resemble the input, to train these networks. The feature extraction capabilities of four encoder-decoder-based models are compared, followed by an analysis of the correlation between the extracted features and diagnostic categories or known medical/radiological markers. Demonstrating that unsupervised encoder-decoder networks can extract diagnostically meaningful features appears to be the central contribution of this study. The authors also claim to have introduced a novel methodology for analyzing MRI images, which they name DUNE.
The input data for the study were sourced from six datasets: UK, TCGA, UPENN, ADNI, UCSF, and SchixConnect. However, the descriptions of how the training data were prepared are scattered across various parts of the paper and lack coherence. They appear in the abstract, introduction, Figure 1a, results section, and the Materials and methods section. Multiple, sometimes contradictory numbers of training examples are mentioned: 21,102 individuals, 3,814 MRI scans, 1,322 individuals, 500,000 healthy individuals, etc. In the Results section, it is mentioned that 800 cases were selected from 19,955, yet another statement refers to n=1,600 scans, and Figure 3 caption mentions the use of all 19,955 cases.
The manuscript states: "To prevent overfitting the model to normal healthy brains, only 800 randomly selected cases were finally kept for the model development (n=1,600 T1 and FLAIR scans)." This

justification is questionable, as reducing training set size is generally counterproductive in preventing overfitting; rather, increasing data volume is a solution to this problem. It seems the reduction was motivated more by a desire to balance the dataset than by overfitting concerns.

We thank the reviewer for this observation regarding the clarity and consistency of our dataset descriptions and training methodology. We have clarified this information in the revised manuscript to improve comprehensibility.
To clarify the numerical discrepancies highlighted by the reviewer:
Datasets for autoencoder training (development):
- UKB: 800 cases selected from 19,955 available (1,600 scans: T1 + FLAIR)
- UPENN: 612 cases (1,224 scans: T1Gd + FLAIR)
- UCSF: 495 cases (990 scans: T1Gd + FLAIR)
- Total training dataset: 1,907 cases, 3,814 scans (80% training, 20% validation)

Datasets for clinical evaluation of the embeddings:
- Internal evaluation (cohorts used for model development)
o UKB (full cohort): 19,955 cases (39,910 scans: T1 + FLAIR) - used for clinical prediction tasks
o UPENN: 612 cases (1,224 scans: T1Gd + FLAIR)
o UCSF: 495 cases (990 scans: T1Gd + FLAIR)
- External evaluation (unseen cohorts)
o TCGA: 168 cases (336 scans: T1Gd + FLAIR) - external glioma validation
o ADNI: 818 cases (818 scans: T1 only) - Alzheimer's disease validation
o SchizConnect: 336 cases (336 scans: T1 only) - schizophrenia validation
- Total evaluation datasets: 21,277 cases, 41,400 scans

Summary of key numbers mentioned in manuscript:
- 21,102 individuals: Refers to UKB evaluation cohort (19,955) + other datasets (1,147) used for embedding evaluation
- 3,814 MRI scans: Specific number of scan sequences used for autoencoder training
- 1,322 individuals: Participants in the three independent validation cohorts (TCGA + ADNI + SchizConnect)

The reviewer correctly points out that our initial explanation regarding "overfitting to normal healthy brains" was imprecise. We appreciate this feedback and provide a clearer rationale for this methodological choice.
The reduction from 19,955 to 800 UKB cases was primarily motivated by the need to maintain balanced representation between healthy and pathological brain morphologies in our training dataset. Our initial experiments using the complete UKB cohort (≈20,000 cases) alongside the glioma datasets (≈1,100 cases) resulted in a severe class imbalance that biased the model toward normal brain morphology. This imbalance led to poor-quality embeddings for tumor cases while maintaining good performance on healthy brains. By downsampling the UKB dataset to match the size of the glioma datasets, we achieved improved quality of tumor embeddings while preserving the model's performance on normal brain images. This strategic balancing was crucial for developing a versatile feature extractor capable of handling both healthy and pathological brain morphologies effectively.
Additionally, we have validation results demonstrating that DUNE's performance generalizes well beyond the training cohort, suggesting that our training dataset size was sufficient for learning robust feature representations.
Changes made:
Methods, Page 4 line 8: new Datasets section : Several datasets served for development and validation of DUNE (Fig 1a, Supplemental Table S1). The model development was made using brain MRI scans from the UKB, UPENN and UCSF datasets. The UKB (UK Biobank) dataset provides extensive clinical and radiological data for a large cohort of approximately 500,000 healthy individuals. Brain MRI (T1 and FLAIR sequences), and clinical data of 19,955 individuals (39,910 scans) were downloaded and preprocessed as described below. In addition to these healthy brains were included scans (T1+gadolinium [T1Gd] and FLAIR) from the glioma cohorts UPENN (UPenn-GBM, 612 cases, 1,224 scans) and UCSF (UCSF-PDGM, 495 cases, 990 scans), to make the model equally capable of extracting features from morphologically abnormal brains.
For model evaluation and generalization assessment, three independent validation cohorts were utilized, representing distinct neurological conditions:
1. Glioma validation cohorts: The previously unseen TCGA-GBM and TCGA-LGG datasets (168 cases combined, 336 scans T1Gd and FLAIR) provided external validation for brain tumor applications.

2. Alzheimer's disease cohort: Data from the ADNI database (818 cases; T1 scans only) enabled evaluation of neurodegenerative disease detection capabilities.
3. Schizophrenia cohort: The SchizConnect database, comprising COBRE and MCIC datasets (336 total cases; T1 scans only), allowed assessment of psychiatric disorder classification performance.
Methods, Page 5 line 30 : new Autoencoder training algorithm section : To prevent overfitting the model to normal healthy brains, only 800 randomly selected cases were finally kept from the UKB cohort for the model development, corresponding to 1,600 scans (T1 and FLAIR sequences). This strategic reduction from the full UKB cohort (19,955 individuals) was crucial to maintain balanced representation between healthy and pathological brain morphologies. Combined with glioma scans from UPENN (1,224 scans) and UCSF (990 scans), the final training dataset comprised 3,814 scans (T1,T1Gd, FLAIR; 80% training, 20% validation).

Further confusion arises from inconsistent statements about validation method. The manuscript mentions an 80/20 train-validation split, suggesting a hold-out validation approach. Elsewhere, a "5-fold cross-validation procedure with oversampling" is metioned, indicating a different evaluation strategy. What is more, the use of oversampling contradicts the earlier decision to reduce the dataset size (from 19,955 to 800), raising questions about the methodological consistency. It is also problematic that the parameter n is used with different meanings and values throughout the manuscript, which is inappropriate from a formal scientific perspective. Overall, the description of data preparation for training procedures is imprecise, inconsistent, and confusing. Scattering this critical information across multiple sections hinders comprehension. The authors should consolidate all information regarding training data preparation into a single, clearly structured section. They must ensure consistency and precision in terminology and methodological description, particularly with regard to data volumes and evaluation protocols.

We thank the reviewer for highlighting this particular point. We acknowledge that the scattered presentation of evaluation strategies across different sections created confusion and apparent contradictions. We have now consolidated and clarified our evaluation methodology in the revised manuscript:
Two distinct validation phases with different strategies:
- Autoencoder training validation: We used an 80/20 train-validation split on the development dataset (3,814 scans from UKB, UPENN, and UCSF). This hold-out validation approach was used exclusively for monitoring autoencoder training convergence and preventing overfitting during the unsupervised feature learning phase.
- Clinical prediction evaluation: We used 5-fold cross-validation with oversampling for evaluating the quality of extracted embeddings in downstream clinical prediction tasks. This cross-validation was applied separately to each clinical evaluation dataset (UKB for phenotype prediction, and independent cohorts for disease-specific validation).

The reviewer correctly identified an apparent contradiction between dataset reduction (19,955 → 800 UKB cases) and subsequent oversampling. We clarify that these operations serve different purposes and occur at different stages:
- Dataset reduction (19,955 → 800): Applied during autoencoder training to balance healthy vs. pathological brain morphologies in the development phase
- Oversampling: Applied during clinical prediction evaluation to address class imbalance in specific prediction tasks (e.g., rare genetic variants, disease vs. control classification)

These strategies are complementary rather than contradictory: the initial reduction ensures balanced representation for feature learning, while oversampling addresses prediction-specific class imbalances during evaluation.
We also acknowledge the inappropriate use of parameter "n" with different meanings throughout the manuscript. We have standardized our terminology in the revised version:
- "Participants" or "cases" for individual subjects
- "Scans" or "sequences" for individual MRI acquisitions
- "Samples" only when referring to individual data points in machine learning contexts
- Consistent numerical references with clear context for each usage

Changes made:
Methods, Page 5 line 30 : new Autoencoder training algorithm section
Methods, Page 6 line 7 : new Patient-level embedding generation section
Methods, Page 6 line 19 : new Statistical analysis and embedding evaluation section

The manuscript deviates from the standard IMRAD (Introduction, Methods, Results, and Discussion) structure. Specifically, the Materials and Methods section is positioned after the description of the conducted experiments and the Results section. This significantly hampers readability, as readers are presented with experimental outcomes before being given a clear understanding of the methods used to obtain them. Fragmentary details about the preparation of training and validation datasets appear in the premature Results section. These methodological descriptions should be consolidated into a coherent and precise Materials and Methods section placed before the Results section. Moreover, the Results section includes interpretative statements and conclusions - such as "the UNET model outperformed the other autoencoders" and "Variational autoencoders showed similar results" - that precede the presentation of the corresponding quantitative experimental results. Such conclusions
should be reserved for the Discussion section and must be explicitly supported by quantitative results presented beforehand in the Results. The current structure and order of the content make the article difficult to follow and give it a disorganized feel.
We thank the reviewer for this important feedback regarding manuscript structure and organization. We have completely reorganized the manuscript to follow the standard IMRAD structure. The Materials and Methods section has been moved to precede the Results section, ensuring that readers have a clear understanding of our methodology before encountering experimental outcomes.
We have consolidated all methodological descriptions that were previously scattered across multiple sections into a comprehensive Materials and Methods section. This includes:
- Complete dataset descriptions with clear numerical references
- Detailed preprocessing pipeline
- Autoencoder architecture specifications
- Training algorithms and hyperparameters
- Statistical analysis and evaluation frameworks
- Validation strategies for both autoencoder training and clinical prediction

The reviewer correctly identified that our Results section contained interpretative statements and conclusions that should be reserved for the Discussion. We have revised the Results section to present quantitative findings objectively, moving all interpretative statements and comparative conclusions to the Discussion section where they are properly supported by the previously presented quantitative results.

Additionally, some of the terminology used throughout the manuscript is vague, inconsistently applied, or insufficiently defined. The authors frequently use the term embeddings in reference to features extracted by neural networks. While a partial explanation is offered in the Feature Extraction subsection, the manuscript alternates between the terms features and embeddings, which may confuse readers. Furthermore, the terms radiomic, genetic and diagnostic embeddings/features are introduced without adequate explanation.
We thank the reviewer for highlighting these important terminological inconsistencies. We acknowledge that our inconsistent use of terminology, particularly the alternation between "features" and "embeddings," created confusion and reduced manuscript clarity. We have systematically revised the manuscript to establish clear and consistent definitions for all technical terms. The following standardized terminology is now used throughout:
- "Features": Low-level numerical characteristics extracted from individual MRI sequences at the autoencoder bottleneck layer (e.g., 1,000-5,000 values per sequence)
- "Embeddings": High-level patient-specific representations created by concatenating features from multiple MRI sequences to generate comprehensive patient signatures (e.g., combined T1 + FLAIR features)
- "Radiomics": Traditional quantitative imaging features extracted using established mathematical algorithms (texture, shape, intensity distributions)

The authors also use the expressions high-dimensional and low-dimensional embeddings, which are not precisely defined and appear to be used in a loosely conceptual sense. It seems they are referring to types of data commonly described in machine learning as unstructured, poorly-structured, or well-structured. This imprecise terminology introduces ambiguity into the manuscript. For instance, the very first sentence of the abstract states: "Magnetic resonance images (MRI) of the brain exhibit high dimensionality that pose significant challenges for computational analysis." This phrase is problematic: MRI data can be 2D, 3D, or 4D (e.g., including temporal dynamics). In this meaning of the term dimensionality, the statement is

misleading and may be misunderstood. As currently written, it confuses dimensionality in the mathematical sense with data structure complexity.
We thank the reviewer for this observation regarding our imprecise use of dimensionality terminology. We agree that our terminology inappropriately conflated mathematical dimensionality with data complexity, creating confusion and ambiguity. The reviewer correctly identified the misleading opening sentence of our abstract as a prime example of this confusion. We have revised this and similar statements throughout the manuscript to distinguish clearly between spatial dimensionality and data complexity.
Changes made:
Abstract, page 2 line 9: "Magnetic resonance images (MRI) of the brain contain complex data that pose significant challenges for computational analysis."
Introduction, page 3 line 16: "MRI datasets are characterized by their computational complexity, stemming from the large number of voxels per image (≥5 million voxels per sequence) and the multisequence nature of clinical protocols (T1, FLAIR, contrast injection sequences, etc.). This high-dimensional space, combined with the subtle morphological patterns that encode clinical information, necessitates sophisticated computational approaches for effective analysis."
Throughout the manuscript: We have standardized our terminology to use:
- "High-dimensional data" when referring to the mathematical concept of many variables/features (e.g., millions of voxels)
- "Complex data" or "computationally complex" when referring to the challenging nature of processing and analyzing neuroimaging data
- "Low-dimensional embeddings" when referring to the reduced feature space (1,000-5,000 features) generated by the autoencoders

The authors state that they "introduce DUNE, a neuroimaging-oriented encoder designed to extract deep features from multisequence brain MRIs." However, the manuscript primarily presents comparisons between existing and modified versions of well-known encoder-decoder neural network architectures, such as U-Net. The preprocessing methods applied to the MRI data are described only superficially and without sufficient justification for their use. Therefore, it remains unclear what exactly DUNE is, how it differs from the established architectures presented, and what is its novel contribution.

We thank the reviewer for this important critique regarding the clarity of DUNE's contribution and novelty. We acknowledge that our presentation created confusion about what DUNE actually represents. DUNE is not a single novel neural network architecture, but rather a comprehensive workflow that integrates three core components to transform raw brain MRI scans into standardized, clinically-relevant patient-level embeddings: (1) a standardized preprocessing pipeline using ANTs that ensures consistent image quality and spatial alignment across different acquisition protocols and scanner types, (2) feature extraction using the U-AE architecture (UNet without skip connections) identified through systematic benchmarking as optimal for clinical embedding generation, and (3) multi-sequence feature aggregation through sequential processing and concatenation, providing operational flexibility for incomplete imaging datasets.
DUNE's primary novel contributions are: the counter-intuitive finding that reconstruction quality does not predict embedding quality for clinical applications (U-AE demonstrated poorest reconstruction but best clinical performance), the demonstration of robust performance across multiple disease areas and institutions, and the discovery that synthetic sequence generation can enhance embedding quality when sequences are missing. Through comprehensive validation across healthy volunteers, glioma patients, Alzheimer's disease, and schizophrenia cohorts, we demonstrate that this integrated workflow generates embeddings with superior clinical relevance compared to traditional radiomics approaches and individual autoencoder architectures evaluated in isolation.
Changes made:
Abstract, page 2 line 22: "We introduce DUNE, a neuroimaging-oriented workflow that transforms raw brain MRI scans into standardized compact patient-level embeddings through integrated preprocessing and deep feature extraction"
Introduction, page 3 line 35: “In this study, we introduce DUNE (Deep feature extraction by UNet-based Neuroimaging-oriented autoEncoder), a comprehensive neuroimaging workflow that transforms multisequence raw brain MRI scans into standardized compact patient-level embeddings. The workflow integrates standardized preprocessing, unsupervised feature extraction using a UNet-based autoencoder, and multi-sequence feature aggregation to generate comprehensive patient-level embeddings”.
Results section, page 4 line 3:

new "Study overview" subsection: “DUNE represents a workflow that transforms multisequence brain MRI data into standardized compact patient-level embeddings through integrated preprocessing, feature extraction and agregation, addressing MRI acquisition variability across institutions. An overview of the workflow for development and evaluation is presented in Fig 1a.”
Methods section, page 6 line 7: new "Patient-level embedding generation" subsection

The analysis may be confounded by the way pathological categories are mapped to different source datasets. Since certain brain disorders are primarily represented within specific databases, the observed embeddings may inadvertently capture dataset-specific characteristics - such as imaging protocols, scanner types, or demographic biases - rather than pathologically meaningful features. The manuscript does not address this potential source of bias.
We thank the reviewer for raising this important concern about potential confounding between pathological categories and dataset-specific characteristics. This is indeed a critical consideration in multi-institutional neuroimaging studies.
We acknowledge that certain brain disorders are primarily represented within specific databases in our study design. However, our methodological approach tends to mitigate this potential source of bias. First, the preprocessing pipeline using ANTs was specifically designed to harmonize images across different acquisition protocols and scanner types, effectively reducing technical variability that could confound pathological signals.
Second, multiple lines of evidence suggest that DUNE embeddings capture biological rather than technical characteristics. Our embeddings successfully predict fundamental biological features (sex, IDH1 or APOE4 status) across different datasets, suggesting that the model captures fundamental biological patterns rather than dataset-specific artifacts. Moreover, DUNE demonstrates robust performance on external validation cohorts acquired using different protocols than training datasets (TCGA, ADNI, SchizConnect). If embeddings were primarily capturing scanner or protocol differences, we would expect degraded performance on these cross-institutional predictions, which we do not observe.
Furthermore, the foundation model paradigm in machine learning increasingly relies on training with heterogeneous, multi-institutional data without extensive harmonization to remove technical variations. This approach enhances model robustness by learning representations that generalize across diverse acquisition conditions. Our validation demonstrates that DUNE embeddings maintain clinical relevance across independent cohorts with different imaging protocols, supporting their biological rather than technical basis.
Changes made:
Discussion, Page 16 line 42: Another potential limitation is the risk for dataset-specific confounding, as certain diseases are primarily represented within specific institutional cohorts, potentially leading to embeddings capturing technical rather than pathological characteristics. However, we implemented strategies to mitigate this risk, and multiple lines of evidence suggest that DUNE embeddings capture biological rather than technical features. First, we employed the ANTs-based preprocessing pipeline, which harmonizes images across different acquisition protocols and scanner types, thus reducing technical variability. Additionally, DUNE successfully predicts fundamental biological features (for example sex, IDH1 or APOE4 status) across different datasets, and demonstrates robust performance on external validation cohorts acquired using different protocols than training datasets (TCGA, ADNI, SchizConnect). If embeddings primarily encoded scanner-specific artifacts, we would expect degraded performance on these cross-institutional predictions, which we do not observe.

While the authors claim to introduce a novel tool/methodology - DUNE - for extracting deep features from multisequence brain MRIs, the manuscript does not clearly distinguish this work from previously published studies, including their own earlier preprint available on medRxiv (https://www.medrxiv.org/content/10.1101/2025.02.24.25322787v1).

We thank the reviewer for bringing attention to the medRxiv preprint. We clarify that the medRxiv preprint is the same work as the current manuscript submitted to GigaScience, not a separate previous publication. The medRxiv preprint represents an earlier version of this manuscript that was posted to enable early sharing of our findings with the scientific community during the peer review process.

At present, the article is not suitable for publication. The authors need to revise the manuscript thoroughly: restructuring it according to standard scientific conventions, improving logical flow and clarity, and using precise language when describing the data, their properties, and the applied methods. Furthermore, the claimed contributions - particularly the nature and novelty of DUNE - must be clearly defined and substantiated.
Despite the concerns outlined above, the manuscript addresses an important and timely problem in the field of medical diagnosis and machine learning. The idea of leveraging unsupervised encoder-decoder networks to extract diagnostically relevant features from multisequence MRI is promising, and with appropriate clarification, restructuring, and methodological precision, this work has the potential to make a meaningful contribution. I encourage the authors to revise the manuscript thoroughly, with particular attention to methodological transparency, structural organization, and clearer articulation of the novel aspects. I look forward to seeing a more refined version of this study in a future submission.

Editorial Manager®

Cookie Preference Center

We use cookies which are necessary to make our site work. We may also use additional cookies to analyze, improve and personalize our content and your digital experience. For more information, see our Cookie Policy.

You may choose not to allow some types of cookies. However, blocking some types may impact your experience of our site and the services we are able to offer. See the different category headings below to find out more or change your settings.

Allow all

Manage Consent Preferences

Strictly Necessary Cookies

Always active

These cookies are necessary for the website to function and cannot be switched off in our systems. They are usually only set in response to actions made by you which amount to a request for services, such as setting your privacy preferences, logging in or filling in forms. You can set your browser to block or alert you about these cookies, but some parts of the site will not then work.

Functional Cookies

These cookies enable the website to provide enhanced functionality and personalisation. They may be set by us or by third party providers whose services we have added to our pages. If you do not allow these cookies then some or all of these services may not function properly.

Performance Cookies

These cookies allow us to count visits and traffic sources so we can measure and improve the performance of our site. They help us to know which pages are the most and least popular and see how visitors move around the site.

Targeting Cookies

These cookies may be set through our site by our advertising partners. They may be used by those companies to build a profile of your interests and show you relevant adverts on other sites. If you do not allow these cookies, you will experience less targeted advertising.

Cookie List

Clear

Apply Cancel

Consent Leg.Interest

Confirm my choices

Powered by onetrus
