# Supplementary material for: DUNE: a versatile neuroimaging encoder captures brain complexity across 3 major diseases: cancer, dementia, and schizophrenia
Source: Gigascience. 2025 Oct 15;14:giaf116. doi: 10.1093/gigascience/giaf116 (PMC12527335; doi:10.1093/gigascience/giaf116)
Supplement: giaf116_Reviewer_1_Report_Original_Submission — Laura Caquelin -- 4/14/2025 [file giaf116_reviewer_1_report_original_submission.pdf]

Reviewer Report

Title: DUNE: a versatile neuroimaging encoder captures brain complexity across three major diseases: cancer, dementia and schizophrenia

Version: Original Submission **Date:** 4/14/2025

Reviewer name: Laura Caquelin

Reviewer Comments to Author:

Reproducibility report for: DUNE: a versatile neuroimaging encoder captures brain complexity across three major diseases: cancer, dementia and schizophrenia
Journal: Gigascience
ID number/DOI: GIGA-D-25-00076
Reviewer(s): Laura Caquelin, Department of Clinical Neuroscience, Karolinska Institutet, Sweden [Wrote the report and reproduced the results]
Gustav Nilsonne, Department of Clinical Neuroscience, Karolinska Institutet, Sweden [Reviewed the final report]

---

1. Summary of the Study
The manuscript introduces DUNE , a neuroimaging-oriented (UNet-based) encoder designed to extract deep features from multisequence brain MRIs, enabling their analysis with machine learning models. It was trained on scans of healthy and glioma-affected brains to generate low-dimensional representations. These embeddings were then used to train models predicting various clinical variables and tested on independent cohorts to assess generalization.

---

2. Scope of reproducibility
According to our assessment the primary objective is: "to test prediction on MRI embeddings accurately predict clinical phenotypes of healthy individuals." (page 12)
- Outcome: the predictive performance of machine learning models trained on different MRI-derived embeddings for clinical phenotype prediction.
- Analysis method outcome: "The predictions from all validation folds were concatenated to generate ROC curves and compute performance metrics: weighted F1-scores for categorical variables, RÂ^2 scores for quantitative variables, and C-indexes and Integrated Brier Scores for survival variables. The predictive performance of models using different embeddings was compared using Wilcoxon signed-rank tests with Bonferroni correction for multiple comparisons." (page 26)
- Main result: "Overall, the predictions were best with U-AE embeddings (0.384Â±0.27%), which significantly outperformed radiomics (0.144Â±1.58, p<0.001, Fig 3a). The embeddings encoded by other models (UNET, U-VAE, VAE) resulted in weaker predictions. The performances greatly varied between the different subgroups of clinical variables but showed small variation across variables within each category (Fig 3a), which tended to be highly intercorrelated (Supplemental Figure S2)." (page 12)

---

3. Availability of Materials
a. Data
- Data availability: Not open, not yet publicly available, shared on the FTP server
- Data completeness: Complete = all data necessary to reproduce main results are available
- Access Method: Private journal dropbox
- Repository: -
- Data quality: The data files have been shared and appear sufficient for running the analyses. However, no metadata is provided to describe the content, structure, or origin of the files which limits interpretability and reusability.
b. Code
- Code availability: The code for the DUNE tools is publicly available, while the code required to reproduce the main results of the manuscript is private.
- Programming Language(s): Python
- Repository link: https://github.com/gevaertlab/BrainMR_AE_datafusion/ The code to reproduce the results is hosted on a private FTP server.
- License: Apache 2.0
- Repository status: Public for DUNE tools, Private for result reproduction code.
- Documentation: No documentation shared
----
4. Computational environment of reproduction analysis
- Operating system for reproduction: MacOS 14.7.4
- Programming Language(s): Python
- Code implementation approach: Using shared code
- Version environment for reproduction: Python 3.13.3
----
5. Results
5.1 Original study results
- Results 1: Results presented in figure 3A in the manuscript.
5.2 Steps for reproduction
-> Identify the numerical values of inferential statistical results in relation to the conclusions of Figure 3A.
- Issue 1: Numerical p-values are not provided, only indicated with asterisks in Figure 3A.
-- Resolved: The figure presents significance markers but does not report the exact p-values used for model comparisons, same for the means and standard errors across variables.
After request, the authors provided the values in files shared on private FTP servers.
- Issue 2: The raw data necessary to reproduce Figure 3A are not available.
-- Resolved: The dataset containing prediction scores (RÂ² or F1-scores) across variables in each category for each model is not shared in the GitHub repository.
After request, the authors provided the raw data, and the script shared on the private FTP servers.
-> Run the Python script provided to reproduce the results of Figure 3A.
- Issue 3: Missing package installations required to run the code.
-- Resolved: There was no README file specifying the required dependencies. As a result, I had to

manually identify and install the necessary packages. Some dependencies were sub-modules, so I had to search for the full package to install. For instance, sklearn.metrics is part of the larger scikit-learn package, sksurv.metrics part of scikit-survival package, and statannotations.Annotator part of statannotations package. Also statsmodels was also needed for certain statistical functions.
- Issue 4: Fixed paths that don't work on other computers.
-- Resolved: In the original code, there were fixed file paths (/Users/tom/drive/...) that only worked on one specific computer. This caused problems when running the code on other computers. To fix this, I changed the code to use relative paths, which are based on where the script is located. This way, the code can be run on any computer without needing to change the paths each time.
------------------ Start of script ------------------

```
# Configuration
# [Modification by LC]BEGIN------------
#os.chdir("/Users/tom/drive/2-postdoc/1-projects/brain_autoencoder/MS")
os.getcwd()
OUTPUT_DIR = "./GigaScience_review"
# [Modification by LC]END------------
FONTSIZE = 16
UKB_FONTSIZE = 16
UKB_FONTWEIGHT = 'regular'
SHADOW = False
# Paths
# [Modification by LC]BEGIN------------
#DATA_PATHS = {
#"METADATA_FILE": "/Users/tom/drive/2-postdoc/1-
projects/brain_autoencoder/MS/GigaScience/variable_metadata.csv",
#"PREDICTIONS_DIR": "/Users/tom/drive/2-postdoc/1-projects/brain_autoencoder/MS/GigaScience",
#}
SCRIPT_DIR = os.path.dirname(os.path.abspath(__file__))
DATA_PATHS = {
"METADATA_FILE": os.path.join(SCRIPT_DIR, "variable_metadata.csv"),
"PREDICTIONS_DIR": os.path.join(SCRIPT_DIR),
}
# [Modification by LC]END------------
```

------------------- End of script -------------------
5.3 Statistical comparison Original vs Reproduced results
- Results: From the python script shared, Figure 3, the category performance_stats and the statistical_comparison_pvalues were obtained.
Comparison between the category_performance_stats and the statistical_comparison_pvalues files from the original results and the reproduced ones was conducted using R (script attached to the review).
- Comments: No particular comments.
- Errors detected: Corresponding to the sentence "Overall, the predictions were best with U-AE embeddings (0.384Â±0.27%), which significantly outperformed radiomics (0.144Â±1.58, p<0.001, Fig

3a)." (page 13):
-- The reported value for radiomics (0.144) should be rounded to 0.145 based on the given data (0.144650928571429). No rounding error is present for the U-AE value, which is correctly reported as 0.384 based on the given data (0.384448357142857).
-- There seems to be an inconsistency in the reported standard errors for both the U-AE and radiomics models. The paper reports a standard error of "0.384 Â± 0.27%" for U-AE and "0.144 Â± 1.58%" for Radiomics. Based on the provided data, the actual standard errors for these models are much higher: 1.63% (0.0162718529517984) for U-AE and 9.47% (0.0947012592300183) for radiomics.
- Statistical Consistency:
-- Graphically, the reproduced Figure 3A appears to correspond to the original one.
-- For the data in 0-category_performance_stats.csv, no differences were observed between the original results and the reproduced values.
-- For the data in 0-statistical_comparison_pvalues.csv, only one minor numerical difference was found: the p-value for category "psy", model "U-AE/RAD", differed slightly between the original (8.983406e-01) and the reproduced (8.967698e-01) results. All other values matched exactly.
----
6. Conclusion
- Summary of the computational reproducibility review
The computational reproducibility review confirmed that the results in the original study related to the main objective defined according to our assessment were reproducible using the data and code provided by the authors. The graphical representation in Figure 3A was successfully reproduced, and no differences were found between the original and reproduced values for the category_performance_stats dataset.
However, a minor numerical difference was detected in the statistical_comparison_pvalues dataset for the "psy" category, U-AE/RAD model, where the p-value slightly differed between the original (8.983406e-01) and the reproduced (8.967698e-01) results.
In addition, there are inconsistencies with rounding and discrepancies in the reported standard errors, which need to be resolved.
In summary, the results were almost fully reproducible using the original code, with only a minor discrepancy.
- Recommendations for authors
We were able to reproduce the study because we had access to the data and code provided on the private journal FTP server. To ensure that the study remains fully reproducible in the future, the following recommendations are strongly advised:
-- Make the code and data to reproduce all the paper openly available to the public to allow anyone to reproduce the figures and analysis of the paper.
-- Provide a README file or requirements.txt file to list all dependencies and ensure that the code can be run without issues. This is particularly important when using sub-modules and non-standard libraries. Clear instructions should also be included regarding how to set up the environment and run the analysis, we suggest:
--- A brief description of each script or analysis pipeline.
--- An indication of which figure, table, or result in the manuscript each script corresponds to.

--- Clear instructions on how to execute the analyses in the correct order, if applicable.
-- Metadata: For the datasets used or generated by the scripts, it would be helpful to include accompanying metadata files that explain:
--- The definition of each variable name.
--- The origin of each dataset (raw, processed, etc).
--- Any preprocessing steps applied before analysis.
-- It is also recommended to document any software versions used in the analysis, as this can help avoid issues with version mismatches in future reproductions.
These improvements would enhance the reproducibility and transparency of the work.

Methods

Are the methods appropriate to the aims of the study, are they well described, and are necessary controls included? Choose an item.

Conclusions

Are the conclusions adequately supported by the data shown? Choose an item.

Reporting Standards

Does the manuscript adhere to the journal's guidelines on [minimum standards of reporting?] Choose an item.

Choose an item.

Statistics

Are you able to assess all statistics in the manuscript, including the appropriateness of statistical tests used? Choose an item.

Quality of Written English

Please indicate the quality of language in the manuscript: Choose an item.

Declaration of Competing Interests

Please complete a declaration of competing interests, considering the following questions:

- Have you in the past five years received reimbursements, fees, funding, or salary from an organisation that may in any way gain or lose financially from the publication of this manuscript, either now or in the future?
- Do you hold any stocks or shares in an organisation that may in any way gain or lose financially from the publication of this manuscript, either now or in the future?

- Do you hold or are you currently applying for any patents relating to the content of the manuscript?
- Have you received reimbursements, fees, funding, or salary from an organization that holds or has applied for patents relating to the content of the manuscript?
- Do you have any other financial competing interests?
- Do you have any non-financial competing interests in relation to this paper?

If you can answer no to all of the above, write 'I declare that I have no competing interests' below. If your reply is yes to any, please give details below.

1. No 2. No 3. No 4. No 5. No 6. No

I agree to the open peer review policy of the journal. I understand that my name will be included on my report to the authors and, if the manuscript is accepted for publication, my named report including any attachments I upload will be posted on the website along with the authors' responses. I agree for my report to be made available under an Open Access Creative Commons CC-BY license (http://creativecommons.org/licenses/by/4.0/). I understand that any comments which I do not wish to be included in my named report can be included as confidential comments to the editors, which will not be published.

Choose an item.

To further support our reviewers, we have joined with Publons, where you can gain additional credit to further highlight your hard work (see: https://publons.com/journal/530/gigascience). On publication of this paper, your review will be automatically added to Publons, you can then choose whether or not to claim your Publons credit. I understand this statement.

Yes Choose an item.
