# Supplementary material for: DUNE: a versatile neuroimaging encoder captures brain complexity across 3 major diseases: cancer, dementia, and schizophrenia
Source: Gigascience. 2025 Oct 15;14:giaf116. doi: 10.1093/gigascience/giaf116 (PMC12527335; doi:10.1093/gigascience/giaf116)
Supplement: giaf116_Reviewer_2_Report_Original_Submission — Huaiqiang Sun -- 4/22/2025 [file giaf116_reviewer_2_report_original_submission.pdf]

Reviewer Report

Title: DUNE: a versatile neuroimaging encoder captures brain complexity across three major diseases: cancer, dementia and schizophrenia

Version: Original Submission **Date:** 4/22/2025

Reviewer name: Huaiqiang Sun

Reviewer Comments to Author:

This work introduces DUNE, a versatile deep-learning model designed to extract low-dimensional embeddings from brain MRIs. The model's ability to generalize across multiple diseases, including glioma, Alzheimer's, and schizophrenia, is a significant strength, with broad potential clinical applications. However, there are areas where the manuscript can be improved:

1. While the manuscript compares DUNE to radiomics, a traditional feature extraction method, it would benefit from a comparison with more end-to-end deep learning models or architectures, such as Convolutional Neural Networks (CNNs) or Vision Transformers (ViTs). These models might perform similarly or better for the tasks at hand. This comparison would help establish the superiority or uniqueness of DUNE in terms of its capabilities and performance.
2. The manuscript mentions the use of synthetic data to augment the MRI data, with noted improvements in performance. However, more details on the synthetic data generation process are needed to strengthen the manuscript. A more in-depth explanation of how the synthetic data was created and how it contributes to the model's performance would provide greater clarity.
3. Due to the absence of skip connections, the images reconstructed by the U-AE model inevitably suffer from a loss of details. It would be helpful to explore whether constraints can be imposed on the loss function to encourage the network to preserve image details. For instance, incorporating distance function constraints (as in Sun et al., 2020, 10.1002/hbm.26098) could be an approach to ensure better retention of details for segmented tissue map.

Methods

Are the methods appropriate to the aims of the study, are they well described, and are necessary controls included? Choose an item.

Conclusions

Are the conclusions adequately supported by the data shown? Choose an item.

Reporting Standards

Does the manuscript adhere to the journal’s guidelines on minimum standards of reporting? Choose an item.

Choose an item.

Statistics

Are you able to assess all statistics in the manuscript, including the appropriateness of statistical tests used? Choose an item.

Quality of Written English

Please indicate the quality of language in the manuscript: Choose an item.

Declaration of Competing Interests

Please complete a declaration of competing interests, considering the following questions:

- Have you in the past five years received reimbursements, fees, funding, or salary from an organisation that may in any way gain or lose financially from the publication of this manuscript, either now or in the future?
- Do you hold any stocks or shares in an organisation that may in any way gain or lose financially from the publication of this manuscript, either now or in the future?
- Do you hold or are you currently applying for any patents relating to the content of the manuscript?
- Have you received reimbursements, fees, funding, or salary from an organization that holds or has applied for patents relating to the content of the manuscript?
- Do you have any other financial competing interests?
- Do you have any non-financial competing interests in relation to this paper?

If you can answer no to all of the above, write 'I declare that I have no competing interests' below. If your reply is yes to any, please give details below.

I declare that I have no competing interests

I agree to the open peer review policy of the journal. I understand that my name will be included on my report to the authors and, if the manuscript is accepted for publication, my named report including any attachments I upload will be posted on the website along with the authors' responses. I agree for my report to be made available under an Open Access Creative Commons CC-BY license (http://creativecommons.org/licenses/by/4.0/). I understand that any comments which I do not wish to be included in my named report can be included as confidential comments to the editors, which will not be published.

Choose an item.

To further support our reviewers, we have joined with Publons, where you can gain additional credit to further highlight your hard work (see: https://publons.com/journal/530/gigascience). On publication of this paper, your review will be automatically added to Publons, you can then choose whether or not to claim your Publons credit. I understand this statement.

Yes Choose an item.
