# Supplementary material for: DUNE: a versatile neuroimaging encoder captures brain complexity across 3 major diseases: cancer, dementia, and schizophrenia
Source: Gigascience. 2025 Oct 15;14:giaf116. doi: 10.1093/gigascience/giaf116 (PMC12527335; doi:10.1093/gigascience/giaf116)
Supplement: giaf116_Reviewer_3_Report_Original_Submission — Piotr M. Szczypinski -- 5/6/2025 [file giaf116_reviewer_3_report_original_submission.pdf]

Reviewer Report

Title: DUNE: a versatile neuroimaging encoder captures brain complexity across three major diseases: cancer, dementia and schizophrenia

Version: Original Submission **Date:** 5/6/2025

Reviewer name: Piotr M. Szczypinski

Reviewer Comments to Author:

The article addresses the problem of feature extraction from MRI brain images, aiming to support the diagnosis of various brain diseases and pathological conditions. Given the nature of the task, supervised classification networks (such as AlexNet, ResNet, etc.) would typically be considered the primary approach, as these architectures are explicitly designed to extract features enabling optimal discrimination between classes - different pathological brain states.
The authors, however, hypothesize that encoder-decoder neural network architectures (e.g., U-Net and its variants) can effectively extract embedding features relevant to diagnosis. They further propose using unsupervised learning methods, based on the assumption that the reconstructed image should closely resemble the input, to train these networks. The feature extraction capabilities of four encoder-decoder-based models are compared, followed by an analysis of the correlation between the extracted features and diagnostic categories or known medical/radiological markers. Demonstrating that unsupervised encoder-decoder networks can extract diagnostically meaningful features appears to be the central contribution of this study. The authors also claim to have introduced a novel methodology for analyzing MRI images, which they name DUNE.
The input data for the study were sourced from six datasets: UK, TCGA, UPENN, ADNI, UCSF, and SchixConnect. However, the descriptions of how the training data were prepared are scattered across various parts of the paper and lack coherence. They appear in the abstract, introduction, Figure 1a, results section, and the Materials and methods section. Multiple, sometimes contradictory numbers of training examples are mentioned: 21,102 individuals, 3,814 MRI scans, 1,322 individuals, 500,000 healthy individuals, etc. In the Results section, it is mentioned that 800 cases were selected from 19,955, yet another statement refers to n=1,600 scans, and Figure 3 caption mentions the use of all 19,955 cases.
The manuscript states: "To prevent overfitting the model to normal healthy brains, only 800 randomly selected cases were finally kept for the model development (n=1,600 T1 and FLAIR scans)." This justification is questionable, as reducing training set size is generally counterproductive in preventing overfitting; rather, increasing data volume is a solution to this problem. It seems the reduction was motivated more by a desire to balance the dataset than by overfitting concerns.
Further confusion arises from inconsistent statements about validation method. The manuscript mentions an 80/20 train-validation split, suggesting a hold-out validation approach. Elsewhere, a "5-fold cross-validation procedure with oversampling" is metioned, indicating a different evaluation strategy. What is more, the use of oversampling contradicts the earlier decision to reduce the dataset size (from 19,955 to 800), raising questions about the methodological consistency. It is also problematic that the

parameter n is used with different meanings and values throughout the manuscript, which is inappropriate from a formal scientific perspective. Overall, the description of data preparation for training procedures is imprecise, inconsistent, and confusing. Scattering this critical information across multiple sections hinders comprehension. The authors should consolidate all information regarding training data preparation into a single, clearly structured section. They must ensure consistency and precision in terminology and methodological description, particularly with regard to data volumes and evaluation protocols.

The manuscript deviates from the standard IMRAD (Introduction, Methods, Results, and Discussion) structure. Specifically, the Materials and Methods section is positioned after the description of the conducted experiments and the Results section. This significantly hampers readability, as readers are presented with experimental outcomes before being given a clear understanding of the methods used to obtain them. Fragmentary details about the preparation of training and validation datasets appear in the premature Results section. These methodological descriptions should be consolidated into a coherent and precise Materials and Methods section placed before the Results section. Moreover, the Results section includes interpretative statements and conclusions - such as "the UNET model outperformed the other autoencoders" and "Variational autoencoders showed similar results" - that precede the presentation of the corresponding quantitative experimental results. Such conclusions should be reserved for the Discussion section and must be explicitly supported by quantitative results presented beforehand in the Results. The current structure and order of the content make the article difficult to follow and give it a disorganized feel.

Additionally, some of the terminology used throughout the manuscript is vague, inconsistently applied, or insufficiently defined. The authors frequently use the term embeddings in reference to features extracted by neural networks. While a partial explanation is offered in the Feature Extraction subsection, the manuscript alternates between the terms features and embeddings, which may confuse readers. Furthermore, the terms radiomic, genetic and diagnostic embeddings/features are introduced without adequate explanation.

The authors also use the expressions high-dimensional and low-dimensional embeddings, which are not precisely defined and appear to be used in a loosely conceptual sense. It seems they are referring to types of data commonly described in machine learning as unstructured, poorly-structured, or well-structured. This imprecise terminology introduces ambiguity into the manuscript. For instance, the very first sentence of the abstract states: "Magnetic resonance images (MRI) of the brain exhibit high dimensionality that pose significant challenges for computational analysis." This phrase is problematic: MRI data can be 2D, 3D, or 4D (e.g., including temporal dynamics). In this meaning of the term dimensionality, the statement is misleading and may be misunderstood. As currently written, it confuses dimensionality in the mathematical sense with data structure complexity.

The authors state that they "introduce DUNE, a neuroimaging-oriented encoder designed to extract deep features from multisequence brain MRIs." However, the manuscript primarily presents comparisons between existing and modified versions of well-known encoder-decoder neural network architectures, such as U-Net. The preprocessing methods applied to the MRI data are described only superficially and without sufficient justification for their use. Therefore, it remains unclear what exactly DUNE is, how it differs from the established architectures presented, and what is its novel contribution.

The analysis may be confounded by the way pathological categories are mapped to different source

datasets. Since certain brain disorders are primarily represented within specific databases, the observed embeddings may inadvertently capture dataset-specific characteristics - such as imaging protocols, scanner types, or demographic biases - rather than pathologically meaningful features. The manuscript does not address this potential source of bias.

While the authors claim to introduce a novel tool/methodology - DUNE - for extracting deep features from multisequence brain MRIs, the manuscript does not clearly distinguish this work from previously published studies, including their own earlier preprint available on medRxiv (https://www.medrxiv.org/content/10.1101/2025.02.24.25322787v1).

At present, the article is not suitable for publication. The authors need to revise the manuscript thoroughly: restructuring it according to standard scientific conventions, improving logical flow and clarity, and using precise language when describing the data, their properties, and the applied methods. Furthermore, the claimed contributions - particularly the nature and novelty of DUNE - must be clearly defined and substantiated.

Despite the concerns outlined above, the manuscript addresses an important and timely problem in the field of medical diagnosis and machine learning. The idea of leveraging unsupervised encoder-decoder networks to extract diagnostically relevant features from multisequence MRI is promising, and with appropriate clarification, restructuring, and methodological precision, this work has the potential to make a meaningful contribution. I encourage the authors to revise the manuscript thoroughly, with particular attention to methodological transparency, structural organization, and clearer articulation of the novel aspects. I look forward to seeing a more refined version of this study in a future submission.

Methods

Are the methods appropriate to the aims of the study, are they well described, and are necessary controls included? Choose an item.

Conclusions

Are the conclusions adequately supported by the data shown? Choose an item.

Reporting Standards

Does the manuscript adhere to the journal's guidelines on minimum standards of reporting? Choose an item.

Choose an item.

Statistics

Are you able to assess all statistics in the manuscript, including the appropriateness of statistical tests used? Choose an item.

Quality of Written English

Please indicate the quality of language in the manuscript: Choose an item.

Declaration of Competing Interests

Please complete a declaration of competing interests, considering the following questions:

- Have you in the past five years received reimbursements, fees, funding, or salary from an organisation that may in any way gain or lose financially from the publication of this manuscript, either now or in the future?
- Do you hold any stocks or shares in an organisation that may in any way gain or lose financially from the publication of this manuscript, either now or in the future?
- Do you hold or are you currently applying for any patents relating to the content of the manuscript?
- Have you received reimbursements, fees, funding, or salary from an organization that holds or has applied for patents relating to the content of the manuscript?
- Do you have any other financial competing interests?
- Do you have any non-financial competing interests in relation to this paper?

If you can answer no to all of the above, write 'I declare that I have no competing interests' below. If your reply is yes to any, please give details below.

1. No 2. No 3. No 4. No 5. No 6. No

I agree to the open peer review policy of the journal. I understand that my name will be included on my report to the authors and, if the manuscript is accepted for publication, my named report including any attachments I upload will be posted on the website along with the authors' responses. I agree for my report to be made available under an Open Access Creative Commons CC-BY license (http://creativecommons.org/licenses/by/4.0/). I understand that any comments which I do not wish to be included in my named report can be included as confidential comments to the editors, which will not be published.

Choose an item.

To further support our reviewers, we have joined with Publons, where you can gain additional credit to further highlight your hard work (see: https://publons.com/journal/530/gigascience). On publication of this paper, your review will be automatically added to Publons, you can then choose whether or not to claim your Publons credit. I understand this statement.

Yes Choose an item.
